# Upper Atmosphere Dynamics and Drivers of Volatiles Loss from Terrestrial-Type (Exo)Planets

**DOI:** 10.1007/s11214-026-01283-w

**Published:** 2026-03-18

**Authors:** Daria Kubyshkina, M. J. Way, Iannis Dandouras, Helmut Lammer, Antonino Francesco Lanza, Manasvi Lingam, Rumi Nakamura, Moa Persson, Manuel Scherf, Kanako Seki

**Affiliations:** 1 Space Research Institute, Austrian Academy of Sciences, Schmiedlstrasse 6, Graz, 8042 Austria; 2 Space Research and Planetology, Physics Institute, University of Bern, Geselschaftsstrasse 6, Bern, CH-3012 Switzerland; 3 NASA Goddard Institute for Space Studies, 2880 Broadway, New York, 10025 NY USA; 4 Theoretical Astrophysics, Department of Physics and Astronomy, Uppsala University, Uppsala, 75310 Sweden; 5 Institut de Recherche en Astrophysique et Planétologie, Université de Toulouse/CNRS/CNES, Toulouse, France; 6 Center for Space Research and Technology, Academy of Athens, Athens, Greece; 7 INAF-Osservatorio Astrofisico di Catania, via S. Sofia, 78, Catania, 95123 Italy; 8 International Space Science Institute, Hallerstrasse 6, Bern, Switzerland; 9 Swedish Institute of Space Physics, Uppsala, Sweden; 10 Research Center for Advanced Science and Technology (RCAST), The University of Tokyo, Komaba 4-6-1, Meguro-ku, 153-8904 Tokyo Japan; 11 Florida Institute of Technology, Melbourne, USA

**Keywords:** Upper atmosphere, Atmospheric escape, Ion escape, Terrestrial planets

## Abstract

Volatile loss from exoplanetary atmospheres and its possible implications for the longevity of habitable surface conditions is a topic of vigorous debate currently. The vast majority of the habitable zone terrestrial-like exoplanets known to date orbit low-mass M- and K-dwarf stars and are subject to the conditions drastically different to those of terrestrial planets in the Solar System. In particular, they orbit far closer to their host stars than similar planets around G-dwarfs similar to the Sun. Therefore they receive higher X-ray and UV fluxes, even though luminosities of M- and K-dwarfs are lower than those of heavier stars. Furthermore, due to their slower evolution, M-dwarfs retain high activity on the gigayear timescales. The combination of these two effects has led to claims that most terrestrial planets orbiting M-dwarfs may have their atmospheres stripped from the higher X-ray and UV fluxes of their host stars. Opposing this are researchers who point out that volatile inventories for terrestrial exoplanets are ill-constrained, and hence, they may be able to “weather the storm” of these higher X-ray and UV fluxes. In this article, we focus on exploring volatile loss in the upper atmospheres of terrestrial planets in our solar system and applications to those in exoplanetary systems around stars of different types.

## Introduction

The escape of volatile elements is one of the key processes in the atmospheric evolution of terrestrial planets orbiting in the classical habitable zone Kasting et al. (1993), Kopparapu et al. (2013) or closer to their host stars. In the early stages of planetary evolution (∼100-500 Myr), escape can occur through catastrophic impactor events (e.g. Schlichting et al. 2015; Kegerreis et al. 2020) or extreme hydrodynamic outflow (e.g. Owen and Wu 2016; Lammer et al. 2008a, 2020b). Depending on the properties of the host star, planetary mass, and orbit, these can fully erode or significantly deplete the primordial (hydrogen-dominated) atmosphere and delay the formation of steam atmospheres degassed from the solidifying magma ocean and volcanically degassed secondary atmospheres. Following the early phase of extreme escape, atmospheres of low-mass planets, even if otherwise stable, can remain vulnerable to the loss of volatiles over gigayear timescales. This is accomplished via various processes ranging from hydrodynamic outflow powered by stellar high-energy irradiation (X-ray and extreme ultraviolet, EUV, together with XUV; e.g. King and Wheatley 2021) to the erosion by stellar winds (e.g. Kislyakova et al. 2014; Modi et al. 2023). Besides further depleting the bulk of atmospheric species, it can alter the atmospheric composition through the fractionation of lighter and heavier species. This in turn may affect a wide range of processes including global atmospheric dynamics.

The majority of atmospheric escape processes are initiated in upper atmospheres, which are commonly defined as the atmospheric region above the mesopause (the boundary between the mesosphere and thermosphere, where atmospheric temperature reaches its minimum). Here the atmospheric species are no longer affected by turbulent mixing, and their distribution becomes stratified. As this region is dominated by molecular diffusion, it follows that each species, $i$, has its own scale height, $H_{i}$. In the lower atmosphere this can be described by a single scale height, $H$, as eddy diffusion controls the atmospheric mixing. Lower and upper atmosphere can thus be defined as the homo- and hetero-sphere, respectively. The upper atmosphere includes the thermosphere, where most of the high-energy stellar radiation is absorbed, and most photoionization occurs. It also includes the exosphere, where the atmosphere becomes rarefied and transitions into the collisionless regime. For different planets, however, this general definition can imply rather different local conditions, and qualitatively (but not quantitatively) similar upper atmosphere features can lead to drastically different implications for atmospheric dynamics.

**Fig. 1:**
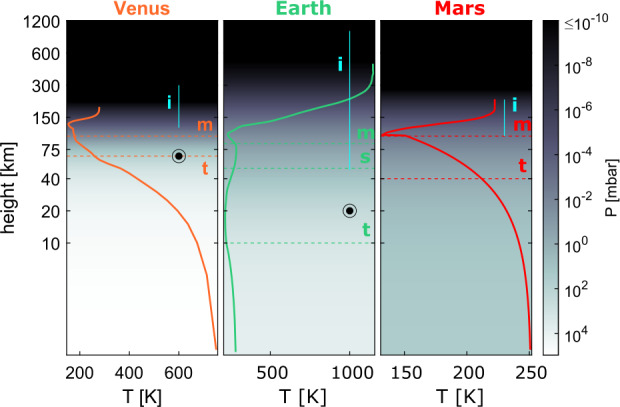
Height scales of atmospheres of Solar System’s terrestrial planets. The solid lines show the temperature profiles against altitude, while the background colour reflects the atmospheric pressure. Horizontal dashed lines denote the average positions of atmospheric boundaries: tropopause (“t”), stratopause (“s”), and mesopause (“m”). Vertical cyan lines depict the extension of ionospheres (“i”) under typical conditions. The “⊙” symbol denotes the 100 mbar level

In Fig. 1, we illustrate the height and pressure scales of Venus, Earth and Mars.^1^ Due to stratification, the lighter atmospheric species are ionised at higher altitudes compared to their heavier counterparts; thus, the heating in the thermosphere is most commonly attributed to photoionisation of atmospheric hydrogen. The peak temperatures in this region depend strongly upon stellar input, planetary mass, and composition and size of the atmosphere defining the atmospheric scale height. Thus, the nitrogen-oxygen dominated atmosphere of Earth is more susceptible to heating compared to the CO_2_ dominated atmospheres of Mars and Venus. This results in a much more extended exosphere and ionosphere. At the same time, the sizes and thermal structures of the atmospheres of Mars and Venus are drastically different despite their similar compositions due to the differences in mass and stellar irradiation.

Even though thermal escape processes are generally associated with photoionisation of H and photodissociation of H_2_, which occur at high altitudes and typically within a narrow altitude range (around $\sim 0.01-10$ mbar pressures), photochemistry is effective over a wider range of altitudes. It becomes particularly relevant if one considers not just the reactions directly involving stellar photons (i.e. photoionisation, -dissociation, and -excitation of various atoms and molecules) but also the cascade of chemical reactions involving their products (e.g. secondary photoionisation, charge exchange, or recombination). This leads to the further redistribution of the absorbed energy, which can drastically change the predictions of atmospheric escape models (e.g. Shematovich et al. 2014; García Muñoz 2023; Kawamura et al. 2024). Therefore, an accurate assessment of atmospheric escape initiated in the upper atmosphere can require consideration of deeper atmospheric layers (at least down to a few bars).

Historically, atmospheric escape mechanisms have been divided into thermal (engaged through bulk atmospheric heating) and non-thermal (driven by the energizing of atmospheric particles through other channels). It is also common to assume that thermal processes dominate atmospheric evolution at early ages (<1 Gyr) while non-thermal processes only matter for the older planets and do not contribute significantly to bulk atmosphere depletion. The real picture is more complicated. Both types of escape processes can occur simultaneously and be powered by the same energy sources, which complicates the evaluation of their relative contributions. For example, it has been shown that the atmospheres of some planets orbiting low-mass stars (hence, on short period orbits even in the habitable zone) may be eliminated by non-thermal erosion from stellar winds (Kislyakova et al. 2014; Modi et al. 2023), even ignoring the contribution from atmospheric heating, which is also expected to last longer in M-dwarf than G-dwarf systems. To help navigate the diversity of atmospheric escape processes, we review the most relevant ones in Sect. 2 and summarise their main drivers (energy sources) and target planets in the Solar System and beyond in Table 1 and Table 2.

**Table 1:** Thermal escape processes

Thermal escape type	Main driver	Major species	Driving process	Affected by mag. field?	Relevant planets and ages
Boil-off	Stellar $L_{\mathrm{bol}}$	bulk atmosphere	Parker-wind-like expansion	no	Close-in & younger than ∼100 Myr^*a*^
Core-Powered Mass Loss	Planet’s luminosity^*b*^	bulk atmosphere	Parker-wind-like expansion	no	Younger than ∼20 Myr^*a*^ or affected by tidal/magnetic heating
XUV-driven hydro. escape	XUV	H, He/bulk^*c*^	Photoionisation of H (XUV heating)	yes	Ages between ∼1-5 Gyr^*a*^
Enhanced Jeans escape	XUV	light species	Photochemistry heating	yes	Ages between ∼1-5 Gyr^*a*^
Classic Jeans escape	XUV	light species	Photochemistry heating	yes	Older than ∼3 Gyr^*a*^

^*a*^Values given here are indicative and actual ages of transition between different thermal escape regimes depend on the type of host star and planetary parameters. For details, see Fig. 22 and Sect. 8.

^*b*^Post-accretion cooling luminosity or heating of planetary interior/lower atmospheric layers through tidal or magnetic interactions with the host star. Approximation by Gupta and Schlichting (2019) generalizes this escape type to bolometric heating; in this case, physics is the same as for boil-off.

^*c*^Under strong irradiation (hence, high escape), the flow of light species (mostly hydrogen) can drag the heavier species along causing bulk outflow; otherwise, only lighter species escape.

**Table 2:** Summary of non-thermal escape processes from terrestrial planets and Titan

Non-thermal escape type	Main drivers	Major species	Driving processes	Planetary magnetisation	Relevant SS bodies
Photochemical escape	XUV	suprathermal (hot) H, D, C, N, O	dissociative recombination, photolysis	both	Mars (H, D, C, N, O), Venus (H, D), Titan (N)
ENA production (charge exchange)	SW, XUV	H, O, C/H	charge exchange	both	Earth/Venus
Ion pick-up	SW	O^+^, C^+^	ionisation	unmagnetized	Venus, Mars
Plasma induced erosion^*a*^	mag. plasma^*b*^	$\mathrm{N}_{2}^{+}$, $\mathrm{CH}_{4}^{+}$, $\mathrm{H}_{2}^{+}$	ionisation	unmagnetized	Titan
Atmospheric sputtering	SW/mag. plasma, XUV	H, He, O, N, CO_2_, Ne, Ar	pickup ion precipitation	unmagnetized	Venus, Mars, Titan
Cold ion outflow (Plasma instabilities)	SW	bulk ionosphere	plasma processes (KHI^*d*^, interchange, magnetic reconnection, etc.)	unmagnetized	Venus, Mars
Polar wind	MF^*c*^	H^+^, He^+^	ambipolar electric field	magnetised	Earth
Plasmaspheric drainage plume	SW	H^+^, He^+^, O^+^, C^+^	SW electric field variations	magnetized	Earth
Auroral outflows	SW, XUV	O^+^, $\mathrm{O}_{2}^{+}$	plasma heating/acceleration	magnetised	Earth

^*a*^The same escape mechanism as ion-pickup but driven by magnetospheric plasma. ^*b*^The interaction between the corotating magnetospheric plasma of Saturn with Titan’s upper atmosphere. ^*c*^Here, an intrinsic magnetic field of a planet. *d* Kelvin-Helmholtz instability.

The timescales on which different escape processes perform, as well as their efficiency, depend crucially on the properties of a specific planet and its host star. These dependencies have been extensively studied for planets in the Solar System, both observationally and theoretically. Still, some of them remain poorly understood despite decades of observations with numerous ground and space-based facilities. Meanwhile, for exoplanets only the strongest atmospheric escape of light species (mainly H and He) may be detected observationally, and the outcomes of such observations remain inconclusive from a theoretical point of view due to a stellar noise and many degeneracies in theoretical models (e.g. Orell-Miquel et al. 2024). Otherwise, the absence of atmospheres around rocky exoplanets (revealed by many recent JWST observations) can put upper limits to the atmospheric evaporation timescales. Moreover, the exoplanets known to date (even if we only consider terrestrial-type planets) occupy a region of parameter space completely different from the planets of the Solar System, both in their masses, sizes and environments surrounding their host stars.

Our perspective on understanding atmospheric escape processes from exoplanets and their effect on planetary atmospheric evolution depends crucially on our understanding of these processes and their sources within the Solar System. Therefore, in Sect. 3 we discuss insights into atmospheric escape processes from observations of Mars (Sect. 3.1), Venus (Sect. 3.2), and Earth (Sect. 3.3). In the following sections, we discuss the main parameters defining the efficiency of different escape processes, how they perform in the Solar System, and what implications they may have for exoplanets. We start by discussing the relevant parameters of various host stars – the main energy sources in their systems – in Sect. 4. We further discuss the role of the planetary mass and size in Sect. 5, the role of atmospheric composition in Sect. 6, and the controversial effects of planetary magnetic field in Sect. 7. We summarise the key points one has to consider when studying atmospheric evolution of exoplanets in Sect. 8. For the convenience of the reader, we summarise the commonly used notations and abbreviations in Table 3 in the Appendix.

## Diversity of Atmospheric Escape Processes

### Thermal Escape

By far the strongest atmospheric mass loss is caused by so-called thermal escape processes. Unified by their main cause, which is the heating of the planetary atmosphere by external or internal sources, they span a wide range of physical regimes, evolutionary timescales, and associated mass loss rates. Among them, one can highlight kinetic mass loss processes (considering the escape of individual particles; enhanced and classic Jeans escape regimes in Table 1) and hydrodynamic atmospheric escape (considering the bulk outflow of atmospheres; boil-off, core-powered, and XUV-driven hydrodynamic escape in Table 1). The former typically describes the escape of the atmospheres of weakly or moderately irradiated terrestrial-like planets on billion-year timescales. The latter describes extreme outflows from young planets and planets orbiting very close to their host stars.

#### Kinetic Jeans Escape

Jeans escape is the longest studied type of kinetic (i.e., considering loss of individual particles) thermal escape (e.g. Jeans 1955; Chamberlain 1963; Öpik 1963; Mihalas and Mihalas 1984). It considers atmospheres in hydrostatic equilibrium and assumes that atmospheric species follow a Maxwell–Boltzmann distribution. Therefore, volatile atmospheric species escape from the region around the exobase, where the atmosphere transitions to collisionless. Moreover, Jeans’ model implies that the peak of the Maxwell–Boltzmann distribution lies well below the escape velocity needed for a particle to leave the planet’s gravitation well $v_{\mathrm{esc}} = \sqrt{\frac{2GM_{\mathrm{pl}}}{r}}$ (here, $M_{\mathrm{pl}}$ denotes the planet’s mass and $r$ the radial distance from the planet’s centre). Thus the atmosphere experiences no significant bulk motion and only the most energetic particles escape.

To treat this problem accurately, one has to solve the Boltzmann equation (e.g. Volkov et al. 2011). This requires heavy numerical computations, therefore the task is often simplified to an isothermal Maxwellian atmosphere (Mihalas and Mihalas 1984); in this case, the problem can be addressed (semi-)analytically and the distribution of atmospheric species can be written as

1 $$ f(\vec{x}, \vec{v}) = n\left ( \frac{1}{\pi v_{\mathrm{th}}^{2}}\right ) ^{3/2} \exp \left ( -\frac{v^{2}}{v_{\mathrm{th}}^{2}} \right ) \,. $$

Here, $v_{\mathrm{th}} = \sqrt{\frac{2k_{b}T}{\mu}}$ describes the thermal velocity of particles of the mass $\mu $ and $n$ denotes the numerical density of said particles. If the velocities of the atmospheric species $v$ exceed the escape velocity at the exobase, they can escape, and the outflow flux can be described as

2 $$\begin{aligned} \Phi = n\left ( \frac{v_{\mathrm{th}}^{2}}{4\pi}\right )^{1/2}(1 + \lambda _{\mathrm{exo}})\exp (-\lambda _{\mathrm{exo}})\,, \end{aligned}$$

3 $$\begin{aligned} \lambda _{\mathrm{exo}} = \frac{v_{\mathrm{esc}}^{2}}{v_{\mathrm{th}}^{2}} = \frac{GM_{\mathrm{pl}}\mu}{k_{b}T_{\mathrm{exo}}r_{\mathrm{exo}}}\,. \end{aligned}$$

The parameter $\lambda _{\mathrm{exo}}$ is commonly referred to as Jeans parameter and represents the ratio of the planet’s escape velocity to the thermal velocity of escaping particles, squared, as calculated at exobase. This definition can also be formulated as the ratio of the planet’s gravitational energy to the bulk thermal energy of its atmosphere. This parameter is widely used in studies of planetary atmospheres for the basic classification of planets’ escape regimes (see Sect. 2.1.3). We note, that both $T_{\mathrm{exo}}$ and $r_{\mathrm{exo}}$ can be very different from the surface/photosphere parameters. $T_{\mathrm{exo}}$ can reach ∼ 1000–10,000 K and $r_{\mathrm{exo}}$ can stretch up to a few or even a few tens of planetary radii (e.g. Van Looveren et al. 2024).

The simplified equations presented above have limitations (see Gronoff et al. 2020, and the references therein). In particular, the atmospheric particle distribution is not exactly Maxwellian even for weakly-irradiated atmospheres. The particles with highest energies constantly escape from the atmosphere (Chamberlain and Smith 1971). Furthermore, atmospheres in general can not be considered isothermal (e.g. Merryfield and Shizgal 1994; Bauer 1971; Johnstone et al. 2018) and do not consist of the uniform species as implied by Eq. (1)–(3). For the mixed atmospheres, this formulation can only provide a crude approximation if particle weight ($\mu $) is substituted by the mean species weight. Finally, particles escape not only from the exobase but from wider range of altitudes around it. Therefore, in the most general case, the analytic approach must be applied with caution.

#### Hydrodynamic Escape

As the atmosphere’s thermal energy budget increases (through the heating by the host star or the high internal energy of the planet), the peak of the Maxwellian distribution shifts to higher velocities and, eventually the atmospheric particles’ mean energy exceeds the bounding of planet’s gravity and particles’ mutual collisions. In this case, deep atmospheric layers get involved into atmospheric escape and it transforms into continuous bulk outflow (e.g. Watson et al. 1981; Volkov et al. 2011). This pushes the exobase to higher (and depleted) altitudes such that they are no longer considered part of the planetary atmosphere.

In this case, the classic Jeans-like prescription breaks, and the atmosphere can be considered in a fluid-like approximation known as hydrodynamic atmospheric escape. For a while, it was used to study the early evolution of terrestrial-like planets in the Solar System (see e.g. Dayhoff et al. 1967; Sekiya et al. 1980; Watson et al. 1981; Tian et al. 2005; Erkaev et al. 2016; Lammer et al. 2020a) and the hydrogen-dominated atmospheres of hot giant exoplanets (see, e.g. Lammer et al. 2003; Lecavelier des Etangs et al. 2004; Baraffe et al. 2004; Yelle 2004; Erkaev et al. 2007, 2015; García Muñoz 2007; García Muñoz and Schneider 2019; García Muñoz 2023; Penz et al. 2008; Cecchi-Pestellini et al. 2009; Murray-Clay et al. 2009; Owen and Jackson 2012; Kubyshkina et al. 2018b, 2024; Caldiroli et al. 2021; Schulik and Booth 2023).

In the early stages of evolution, while the energy budget is high, thermal escape processes are expected to be more effective than the of non-thermal ones, with about an order of magnitude difference in the mass loss rates (e.g. Kislyakova et al. 2014; Modi et al. 2023). In turn, the mass loss rates in the hydrodynamic regime can be orders of magnitude higher than typical Jeans escape values. Hydrodynamic escape can thus be seen as the main driver of bulk atmospheric losses in early planetary evolution (e.g. Lopez et al. 2012; Chen and Rogers 2016; Kubyshkina et al. 2019, 2022; Pezzotti et al. 2021; Gu and Chen 2023). It is expected to have contributed largely to shaping the mass-radius and radius-period distributions of low-mass ($\leq 100$ M_⊕_) exoplanets (e.g. Fulton et al. 2017; Fulton and Petigura 2018; Owen and Wu 2017; Gupta and Schlichting 2019; Mordasini 2020; Affolter et al. 2023).

One can describe (radially symmetric) hydrodynamic atmospheres using a set of conservation equations for the mass, momentum, and energy depending on radial distance $r$ and time $t$

4 $$\begin{aligned} \frac{\partial \rho}{\partial t} + \frac{\partial (\rho v r^{2})}{r^{2}\partial r} =& 0\,, \end{aligned}$$

5 $$\begin{aligned} \frac{\partial \rho v}{\partial t} + \frac{\partial [r^{2}(\rho v^{2}+P)]}{r^{2}\partial r} =& - \frac{\partial U}{\partial r} + \frac{2P}{r}\,, \end{aligned}$$

6 $$\begin{aligned} \frac{\partial E}{\partial t} + \frac{\partial [vr^{2}(E + P)]}{r^{2}\partial r} =& Q + \frac{\partial}{r^{2}\partial r}(r^{2}\chi \frac{\partial T}{\partial r}) - \frac{\partial (\rho U)}{r^{2}\partial r}\,. \end{aligned}$$

Equations (4)-(6) operate on atmospheric density $\rho $, bulk flow $\rho v$, and the total energy $E$ (kinetic + thermal). Other parameters are mean (electron) temperature $T$, thermal pressure $P$, thermal conductivity of the neutral gas, and the gravitational potential $U$ including planet’s and host star’s gravity and the effects of the orbital motion (e.g. Erkaev et al. 2007). The second term on the right-hand of Eq. (6) accounts for the thermal conductivity of the neutral gas.

The parameter $Q\,=\,H\,-\,C$ in Eq. (6) is the sum of all heating and cooling rates given by (photo-)chemical processes included in the specific model. In hydrogen-dominated atmospheres, heating is mainly controlled by the photoionisation of hydrogen molecules (${ \mathrm {H} + h\nu \rightarrow \mathrm {H}^{+} + e^{*}}$) occurring at higher altitudes than for heavier species, and photodissociation of hydrogen atoms. The most typical cooling process in such atmospheres is the collisional excitation of hydrogen atoms (Ly$\alpha $-cooling, ${\mathrm {H} + e^{*} \rightarrow \mathrm {H}^{*} + e}$). For particularly hot planets (typically, hot Jupiters), the heating rate can also be affected by metal line heating (e.g. Fossati et al. 2021; García Muñoz 2023). Further contributions to cooling can be provided by processes such as the free-free interaction of hydrogenic ions (Bremsstrahlung cooling), production of ${\mathrm {H}^{-}}$, recombination of ${\mathrm {H}_{2}^{+}}$, ${\mathrm {H}_{2}}$ line, and ${\mathrm {H}_{3}^{+}}$-cooling (e.g. Kubyshkina et al. 2024).

For secondary atmospheres, contributions to heating can also be provided by the photoionisation of heavier atmospheric species and photodissociation of various molecules. The cooling processes also become more diverse (e.g. water molecule and ${\mathrm {CO}_{2}}$-cooling). Accounting for all the endothermic and exothermic chemical reactions between atmospheric elements requires solving an extensive chemical network along with liquid dynamics equations Eqs. (4)–(6), and modelling realistic atmospheres requires large computational resources. Therefore, most hydrodynamic models employing chemistry beyond that of a hydrogen-helium mixture are restricted to 1D geometry. However, the interaction of hydrodynamic outflow with stellar winds is an essentially 3D problem. Solving the two aspects of the problem within one model is non-trivial. The same concerns apply to deeper atmospheric layers (down to $P\sim $ 1 bar), where a broader range of photochemistry processes and a possible condensation need to be accounted for.

#### Classification of Thermal Escape Processes

Direct modelling of (exo)planetary atmospheres can be a challenging task, both numerically and from the theoretical point of view, as the applicability of a specific model can often be only verified a-posteriori. Therefore, it is common to preface the detailed modelling with a diagnosis based on the basic properties of the planet (such as mass, radius, and irradiation level), allowing one to define the most likely atmospheric escape regime.

A few parameters have been suggested for such analysis. The aforementioned Jeans escape parameter at the exobase level $\lambda _{\mathrm{exo}}$ (Eq. (3)) is historically applied to evaluate terrestrial-like planets and the planets of the Solar System. It was shown that for $\lambda _{\mathrm{exo}} \lesssim 6$, the escape transforms from a Jeans-like to a hydrodynamic regime with a smooth transition from subsonic to supersonic flow (Volkov et al. 2011; Erkaev et al. 2015). Here the retention of the atmosphere on Gyr timescales becomes unlikely, at least under the conditions evaluated. When $\lambda _{\mathrm{exo}}$ becomes ≲ 2-3, there is no stationary hydrodynamic transition from a subsonic to a supersonic flow. In such a case, a fast non-stationary atmospheric expansion results in extreme thermal atmospheric escape rates. This is commonly referred to as a “blow-off” of the upper atmosphere, which is expected to erode on short timescales. Distinct from boil-off and core-powered mass loss, “blow-off” does not consider any specific energy sources but relies solely on the parameters of a planet. However, one should note that the position of the exobase $r_{\mathrm{exo}}$ and the value of $\lambda _{\mathrm{exo}}$ depend on a range of parameters including the molecular weight of the atmospheric species, the planet’s mass, the temperature at the exobase, and atmospheric inflation. The latter two depend on the planet’s internal thermal energy budget (see Sect. 2.3), the planet’s mass, the XUV flux of the host star, and, crucially, on atmospheric composition, as detailed in Sect. 6.

For the present-day terrestrial-like planets in the solar system, the exobase height can be, on astronomical scales, considered $r_{\mathrm{exo}}\,\sim \,R_{\mathrm{pl}}$ or even $r_{\mathrm{exo}}\,=\,R_{\mathrm{pl}}$ if the atmosphere consists of the exosphere only (as at Mercury). Exoplanets, especially young ones, span a much wider range of parameter space. Thus, defining the position of the exobase for any given planet can be non-trivial (in particular, for planets with thick hydrogen-helium-dominated atmospheres). Therefore, $\lambda _{\mathrm{exo}}$ is often generalized to the parameter $\Lambda $ calculated at the planetary photosphere, where the atmosphere becomes optically thin. Namely, $r_{\mathrm{exo}}$ in Eq. (3) is substituted by planetary radius $R_{\mathrm{pl}}$ (Fossati et al. 2017). For compact secondary atmospheres, parameters $\lambda _{\mathrm{exo}}$ and $\Lambda $ are nearly equivalent. However, for close-in sub-Neptune-like planets, hot Jupiters, and highly irradiated secondary atmospheres, $r_{\mathrm{exo}}$ and $R_{\mathrm{pl}}$ (hence, $\lambda _{\mathrm{exo}}$ and $\Lambda $) can differ by order of magnitude. Hydrodynamic simulations show that the transition from blow-off to XUV-driven mass loss mechanism occurs when the gravitational parameter $\Lambda $ drops below 10–30 (e.g. Fossati et al. 2017; Kubyshkina et al. 2018b; Owen and Schlichting 2024). Planets are expected to transition from hydrodynamic to Jeans-like escape at $\Lambda $ values well above 30; the specific value depends strongly on the orbital separation and temperature of the planets (hence, also the type of the stellar host, e.g. Reza et al. 2025).

Due to the limitations of modern observational techniques, most low-mass planets detected to date orbit low-mass M-type stars (especially, low-mass planets in the habitable zone). For such planets, tidal interactions with their host stars become crucial (see Sect. 4.4). Furthermore, such interactions are crucial for planets in very short orbits, where stellar gravity can largely contribute to driving the hydrodynamic outflow or even solely power the outflow (e.g. Koskinen et al. 2022). To account for these effects in the classification of exoplanets, a recent study by Guo (2024) introduces the upgraded Jeans parameter $\lambda ^{*}\,=\,\Lambda \times K$, where parameter $K\,<\,1$ accounts for the correction of the planet’s gravitational potential according to stellar gravity and orbital motion (Erkaev et al. 2007). Guo (2024) predicts that for $\lambda ^{*}\,<\,3$, the hydrodynamic outflow is driven by the tidal forces or the internal thermal energy of a planet (which sources are hard to disentangle for planets on close orbits), while for $\lambda ^{*}>6$ the hydrodynamic escape is XUV-driven.

### Non-Thermal Escape

In the upper atmosphere, collisions become less frequent and the suprathermal component in the velocity distribution can also contribute to the atmospheric escape. There are two major drivers of the non-thermal atmospheric escape, i.e., stellar X-rays and UV radiation (XUV) and stellar wind (SW). The non-thermal escape mechanisms are roughly categorized into two types: the collisional non-thermal escape and the stellar wind-induced escape. As shown in Table 2, which summarizes the non-thermal escape from terrestrial planets, the photochemical escape and charge exchange (production of energetic neutral atoms, ENA, with $v\,>\,v_{\mathrm{esc}}$) are the major collisional non-thermal escape. The SW-induced escape includes the ion pickup, atmospheric sputtering, and ionospheric outflows for unmagnetized planets, as well as the polar wind, auroral outflows, and plasmaspheric drainage plumes (plasma elements detached from the plasmasphere and propagating outwards) for magnetized planets. Plasma-induced erosion is an analogous mechanism to SW-induced ion pick up escape, but is driven by the magnetospheric plasma within Saturn’s magnetosphere. The relative importance of the non-thermal escape mechanisms depends on the planetary conditions such as atmospheric composition and intrinsic magnetic field (MF).

On Earth-like or heavier planets, where the gravitational escape velocity is considerable (11.2 km/s for the Earth), if irradiation is low or moderate, thermal escape in the form of neutral atoms concerns essentially hydrogen. Heavier species, such as oxygen, carbon, and nitrogen, must be accelerated to reach escape velocities by non-thermal escape processes. Thus, atmospheric escape of these heavier species, which often constitute the major part of secondary planetary atmospheres of terrestrial planets, occurs mainly in the form of ion escape. Neutral species in the upper atmosphere can be ionized by the stellar XUV radiation, charge exchange interactions, or electron impact. There are various processes to supply planetary ions to the magnetospheres (e.g. Seki et al. 2015, and references therein).

**Fig. 2:**
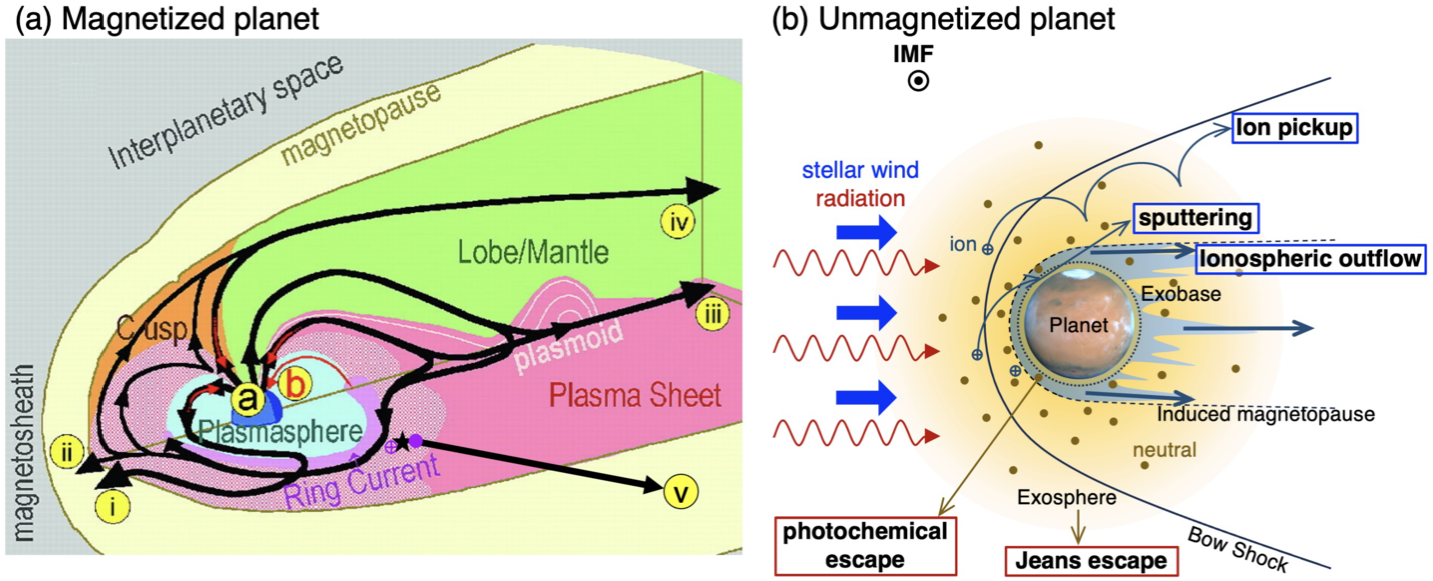
Schematic illustrations of (a) ion escape routes from a magnetized planet (modified from Seki et al. 2001) and (b) atmospheric escape mechanisms from unmagnetized planets. In panel (a),  and  show outflow and return flow from/to the ionosphere, respectively. The escape routes , , and  in the panel (a) result from polar wind and auroral outflows, while routes  and  correspond to the plasmaspheric drainage plume and ENA production by charge exchange between the ring current ions and geocorona

In the case of magnetized planets, such as Earth, the ion escape occurs primarily from polar ionosphere, corresponding to latitudes higher than the subauroral regions as indicated with label  in Fig. 2 (a) (Seki et al. 2001). Once the planetary ions outflow from the polar ionosphere, they can undergo various acceleration and transport in the magnetosphere (solid black arrows), and a significant part of them will eventually escape to interplanetary space (routes , , and ). However, even if the ions have energies above the planet’s escape energy $E_{\mathrm{esc}}\,\sim \,v_{\mathrm{esc}}^{2}$, some of them return to the planetary atmosphere due to the combination of the magnetospheric convection and various plasma processes such as pitch angle scattering by wave-particle interactions as indicated by red arrows and label. Two other mechanisms enable the trapped ions in the planetary magnetic fields to escape: The plasmaspheric drainage plume (route  in Fig. 2 (a)) and ENA production by the charge exchange (route ).

In the case of unmagnetized planets, the stellar wind can interact directly with their upper atmospheres as shown in Fig. 2 (b). In addition to the thermal Jeans escape introduced in Sect. 2.1.1, photochemical escape can be an important mechanism to cause the neutral atmospheric escape. The relative importance of the neutral escape compared to the ion escape largely depends on the stellar XUV radiation and the planetary mass. As the planet’s mass increases, the relative importance of the ion escape to neutral escape generally becomes high due to the large escape energy. Three major ion escape mechanisms from the unmagnetized planet are ion pickup, atmospheric sputtering, and ionospheric outflows. Strictly speaking, the ionospheric outflow includes various plasma processes causing ion acceleration/heating. The basic characteristics of each escape mechanism are outlined in the following subsections.

#### Collisional Non-Thermal Escape

Photochemical escape refers to production of energetic atoms by photochemical reactions such as the dissociative recombination of molecular ions. For low-mass planets, such as Mars, the dissociative recombination itself can make atoms with $E\,>\,E_{\mathrm{esc}}$. For example, the dissociative recombination of $\mathrm{O}_{2}^{+}$

7 $$ {O}_{2}^{+} + e^{-} \rightarrow {\mathrm{O^{*} }+\mathrm{ O^{*}}} $$

is an important process for oxygen escape from Mars (e.g. Lillis et al. 2017); at more massive planets, such as Venus, however, the excess energy by the reaction alone is not sufficient to reach the escape energy. The resultant hot atoms of heavy species, such as O, can indirectly cause the escape of lighter species (H and D) by elastic energy transfer collisions (e.g. McElroy et al. 1982)

8 $$ {\mathrm{O^{*} }+\mathrm{ H }\rightarrow \mathrm{O }+\mathrm{ H^{*}}} $$

In the upper atmosphere, many photochemical reactions should be considered for the theoretical treatment of non-thermal escape (e.g. Fox and Hać 2009). When the atmospheric composition or host stellar conditions change, the relative importance of each reaction will also change (e.g. Nakamura et al. 2023). Thus the efficiency of photochemical escape depends mainly on the planetary mass, atmospheric composition, and stellar XUV radiation as shown in Table 2.

Charge exchange or ENA production generally refers to collisional processes of neutral exospheric species with energetic ions to produce translationally energetic and incompletely thermalized atoms. If the resultant ENAs have energies greater than $E_{\mathrm{esc}}$, they can cause atmospheric escape. If the planet has a global intrinsic field strong enough to form a substantial magnetosphere like Earth, magnetospheric dynamics facilitate various plasma processes accelerating planetary ions. During disturbed periods, such as large magnetic storms at Earth, planetary ions (e.g. O^+^) contribute significantly to the formation of the ring current – the high-energy ions trapped by the intrinsic dipole magnetic field (e.g. Yue et al. 2019, and references therein). The charge exchange between the ring current O^+^ ions and geocorona form ENA by untrapping planetary ions from the magnetic field, which then contribute to the escape of heavy species (oxygen) from the atmosphere (e.g. Keika et al. 2006; Ilie et al. 2013, and references therein). Thus, the ENA production escape efficiency depends both on the stellar XUV radiation, which affects the corona, and stellar wind, which determines the ring current formation as shown in Table 2.

It should be noted that the charge exchange process also indirectly affects atmospheric escape from unmagnetized planets by contributing to upper atmospheric heating through precipitation of stellar wind origin ENA (mainly H and He) (e.g. Halekas 2017). In some cases, it has been pointed out that the precipitation of ENA can cause non-thermal hydrogen escape from low-mass planets like Mars (e.g. Gregory et al. 2023).

#### Stellar Wind Induced Escape from Unmagnetized Planets

The ion pickup process is caused by ionization of the neutral corona. Photochemical reactions, such as dissociative recombination discussed above, contribute not only to the direct escape of neutrals but also to the formation of a hot neutral corona around the planet. The composition of the hot corona can include H, O, C, and N, depending on the planetary mass, atmospheric composition, and stellar XUV conditions.^2^ Once the neutral atoms of the corona are ionised by stellar photon, electron impact, or charge exchange, they can be rapidly accelerated to energies larger than $E_{\mathrm{esc}}$ by the SW-induced electric field. This escape mechanism is referred as the “ion pickup” (e.g. Luhmann et al. 2006; Kislyakova et al. 2014; Curry et al. 2015). Since the number of pickup ions increases with decreasing distance from the planet, it can cause the deceleration of the stellar wind and is sometimes referred to as “mass loading” of the stellar wind. Phenomenologically, pickup ions contribute to a part of polar plumes, where planetary ion flow is primarily accelerated in the direction of the stellar wind electric field (e.g. Dong et al. 2015; Sakakura et al. 2022). Since the trajectory of the ions depends on the location of the ionization, the distribution and efficiency of the ion pickup escape have been studied based on a global simulation of interaction between the stellar wind and the planetary atmosphere either with a hybrid simulation (e.g. Jarvinen et al. 2018) or a combination of a global magnetohydrodynamic (MHD) simulation and a statistical trajectory tracings of test particles (e.g. Curry et al. 2015). The ion pickup escape efficiency depends both on the filling of hot neutral corona influenced by the stellar XUV and the stellar wind conditions, especially on its electric field (e.g. Masunaga et al. 2017) as shown in Table 2.

Atmospheric sputtering is caused by precipitation of the pickup ions into the atmosphere. A significant part of the pickup ions accelerated by the stellar wind electric field can re-enter the atmosphere, where they can sputter neutrals from the region near the exobase (e.g. Luhmann and Kozyra 1991; Curry et al. 2015). The spatial distributions of precipitating pickup ions have a hemispheric asymmetry in terms of the stellar wind electric field direction (e.g. Hara et al. 2013, 2017). The spatial distribution may also depend on crustal magnetic fields (Hara et al. 2018) in a case like Mars. To estimate the escape rates due to atmospheric sputtering caused by pickup ion precipitation, it is necessary to have a description of not only the atmosphere target gas and the exospheric source of the precipitating pickup ions, but also of the stellar wind properties that determine the precipitating pickup ion fluxes and energies. Since atmospheric sputtering efficiently facilitates the loss of neutral atmospheric constituents close to the exobase, it can cause the escape of various species such as H, He, C, O, CO_2_, Ne, and Ar, contributing to mass fractionation of light noble gases (Jakosky et al. 1994). While some models predict a strong increase of atmospheric sputtering loss with increasing stellar XUV flux, observations of heavy ion precipitation did not show an expected XUV dependence (Martinez et al. 2019a) and further studies are needed for clarification.

The cold ion or ionospheric outflows from unmagnetized planets is the general term for phenomena in which planetary-origin plasma is transported from the ionosphere into the induced magnetotail due to some bulk acceleration processes. These processes are mainly related to the momentum transfer from the SW to the upper ionosphere. It leads to energization and, thus, an outward flow of ionospheric ions throughout the planetary tail (Lundin et al. 2007). From spacecraft observations of ions in the tail region of Venus, it is expected that polarized electric fields are also related to the energization process of the ions (Hartle and Grebowsky 1990; Lammer et al. 2006).

Plasma instabilities can also contribute to the escape of cold ionospheric ions. The observation of wave-like structures and plasma clouds or bubbles are indications that Kelvin-Helmholtz (KH; e.g. Wolff et al. 1980; Penz et al. 2004) and interchange (Arshukova et al. 2004) plasma instabilities could be relevant to ion loss processes, especially around unmagnetized planets like Venus and Mars. These instabilities are generated due to disturbances of the interface between the solar wind and the ionopause layer, where the velocity shear between the two separated plasma layers and the curvature of the magnetospheric field can lead to a so-called detachment of ionospheric clouds or bubbles from the ionosphere.

Other energization processes include flux ropes formed by magnetic reconnection, magnetic tension force in the MHD regime, magnetic tension force with unmagnetized ions, ponderomotive force, and combinations of some heating and magnetic mirror effects (e.g. Inui et al. 2019, and references therein). These processes are sometimes referred to as cool ion outflow, momentum transfer, cold ion escape or tailward escape (e.g. Fränz et al. 2015; Dong et al. 2017; Inui et al. 2018). As summarized in Table 2 in Inui et al. (2019), each process has different characteristics such as mass dependence and the direction of acceleration against local magnetic fields, which are often useful to identify the acting process based on observations. However, the relative contribution of each ion energization process is far from understood and upcoming multipoint observations with continuous solar wind monitoring at Mars will be essential for a quantitative understanding.

#### Stellar Wind Induced Escape from Magnetized Planets

In this section, we focus on the effects of the dipolar magnetic field, which is expected to be the most relevant for (exo)planets. Dipole potential declines with distance slower than higher harmonics, hence, it is expected to cause largest-scale effects and therefore dominate the magnetic field effects even if the planetary magnetic field is not strictly dipolar (e.g. Andreeva and Tsyganenko 2016). If the planet has no dipolar magnetic field, the contribution from the higher harmonics/local magnetisation (as e.g. crustal field of Mars) can become non-negligible, but is still expected to have weaker effect on the escape processes. We discuss such cases in more detail in Sect. 7.1.

The most basic escape mechanism associated with magnetised planets is the polar wind, which is caused by the ambipolar electric field in open magnetic field regions. The polar wind is an ambipolar outflow of thermal plasma from the high-latitude ionosphere of planets with a significant global dipole intrinsic magnetic field like Earth (e.g. Yau et al. 2007, and references therein). In the original concept of the polar wind, it consists primarily of electrons and light (H^+^ and He^+^) ions, and Axford (1968) coined the term “polar wind” to describe the supersonic nature of the thermal plasma expansion and outflow, in analogy to the solar wind plasma from the solar corona into interplanetary space. However, observations at Earth indicate that the polar wind also consists of O^+^ ions in addition to H^+^, He^+^, and electrons, which often have small velocities compared to the escape velocity. Various mechanisms accelerate the polar wind ions further at higher altitudes and help them to escape (e.g. Delcourt et al. 1993). There have been many efforts to model the polar wind theoretically (e.g. Lemaire et al. 2007; Tam et al. 2007; Glocer et al. 2012, and references therein). As the polar wind is accelerated by the ambipolar electric field, in general, its velocity correlates with the local electron temperature. The escape rate due to the polar wind depends less on the stellar wind conditions than the auroral outflows described below. The dependence on the stellar XUV through the photoelectron effects is also small, since the escape flux is regulated by a net production of ions (e.g. Kitamura et al. 2015).

Auroral outflows are also prominent atmospheric escape, when a planet has a global intrinsic magnetic field like Earth. High-energy particle precipitations from space are primarily concentrated on the auroral oval (including the cusp, Fig. 2 (a)), a ring-shaped region around the magnetic poles where auroras frequently occur, caused by interactions between the solar wind and Earth’s magnetosphere (e.g. Newell et al. 2009). Both the particle kinetic energy and electromagnetic energy inputs are enhanced in the auroral oval and significant ion escape occurs through various plasma processes (see details, e.g. Gronoff et al. 2020, and references therein).

There are many acceleration and heating mechanisms of the ionospheric ions (e.g. Yau and Andre 1997; Yau et al. 2021, and references therein). To efficiently cause the outflow of heavy ions such as O^+^ and $\mathrm{O}_{2}^{+}$, upward transport of heavy ions from the low- to high-altitude ionosphere is important. The upward transport is often referred to as ion upflows, and both electron precipitations (e.g. Ogawa et al. 2008) and enhanced Joule heating by a strong electric field (e.g. Takada et al. 2021) drive the upflows.

The ions need to be further accelerated or heated at high altitudes to escape, outflowing to the magnetosphere (e.g. Yau and Andre 1997; Yau et al. 2021, and references therein). Morphologically, there are two types of auroral outflows: ion beams accelerated by the parallel potential drop along the magnetic field (e.g. Hull et al. 2003) and conics caused by perpendicular plasma heating and following acceleration by the magnetic mirror force (e.g. Miyake et al. 1996). Wave-particle interactions with various waves including broadband extremely low frequency (BBELF), electromagnetic ion cyclotron (EMIC), electrostatic ion cyclotron (ESIC), lower hybrid (LH), and kinetic Alfven waves can contribute to ion heating. Since the effects of the stellar XUV are important for the source ionosphere, the efficiency of the ion escape by the auroral outflows depends both on the stellar wind conditions and XUV radiation.

**Fig. 3:**
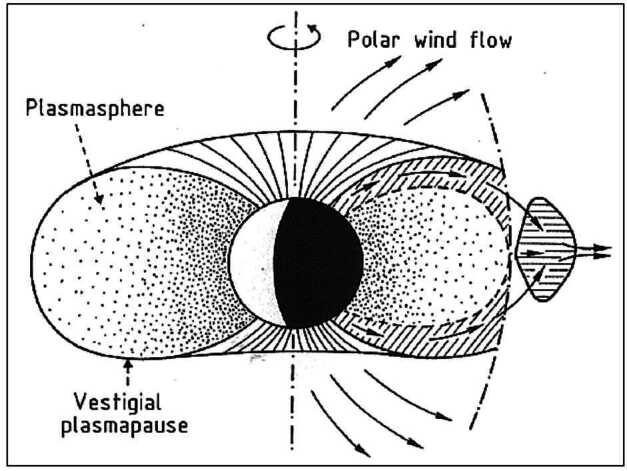
Plasma element being detached from the plasmasphere through the enhanced magnetospheric convection. From Lemaire (2001)

Another path for the cold ion escape is through the plasmaspheric drainage plumes. As shown in Fig. 2 (a), the plasmasphere is the region where the cold planetary plasma is trapped by the global planetary magnetic field and its size is determined by the balance between the corotation and stellar wind induced electric fields. Thus variations of the SW-induced electric field, which depends on the stellar wind velocity and interplanetary magnetic field, can cause the outflow of the plasmaspheric plasma (see Fig. 3), which has important effects on the inner magnetospheric dynamics (e.g. Glocer et al. 2020; Yamakawa et al. 2023). The stream of the cold planetary plasma from Earth’s inner magnetosphere to the dayside magnetopause especially during geomagnetic storms is often referred to as plasmaspheric drainage plumes and is expected to contribute to ion escape from a magnetized planet (e.g. Borovsky and Denton 2008, and references therein). The efficiency of ion escape by plasmaspheric drainage plumes depends both on the strength of the planetary magnetic field and the stellar wind conditions.

### Contribution from the Internal Energy Sources

The formulation of many escape processes discussed above assumes that the upper atmosphere’s thermal energy budget is only supplied by external sources (XUV, SW). Thus, the contribution from the heat sources within the planet’s interior or lower atmosphere has not been considered. However, such heating sources can affect atmospheric mass loss in multiple ways. This “internal” heating can be sustained by a few mechanisms, such as accretion of solids and gravitational contraction during the formation stage (e.g. Ginzburg et al. 2018), decay of the radioactive elements in the planetary core (e.g. Kamland Collaboration et al. 2011; Mordasini et al. 2012), stellar bolometric heating from (e.g. Owen and Wu 2016; Gupta and Schlichting 2019), or tidal and magnetic interactions of a close-in planet with its host star (see details in Sect. 4).

Suppose that the heating is strong and the planetary gravity is low. In that case, the planet’s internal thermal energy can power a hydrodynamic outflow on its own, without any contribution from photochemical heating (boil-off or core-powered mass loss). Such a regime is expected to be short-lived, as it is associated with extreme atmospheric mass loss rates that can overcome the XUV-driven escape by orders of magnitude. This leads to rapid cooling and contraction of the planet (e.g Owen and Wu 2016; Kubyshkina et al. 2020), and quick cessation of the process. Such escape mechanisms can, therefore, lead to the total evaporation of the atmosphere on a short timescale (∼1–100 Myr) or a swift reduction in its size and thermal budget. This mechanism is mainly relevant for primordial hydrogen-dominated atmospheres.

Furthermore, while core-powered mass-loss dominates the escape, the classic XUV-driven escape can be suppressed. The XUV heating, normally occurs in a narrow range of altitudes near the planetary photosphere ($R_{ \mathrm{pl}}$), and its specific position and extension (reduced to a single radius $R_{\mathrm {eff}}$ in common approximations, e.g. Watson et al. 1981; Erkaev et al. 2007) is defined by atmospheric opacity and the local pressure gradient. The heat sources located below the photosphere lead to atmospheric inflation, the pressure gradient becomes shallow, and the atmosphere remains dense ($\rho >\sim 10^{12}$ cm^−3^ Kubyshkina et al. 2018b; Owen and Schlichting 2024) and opaque up to altitudes order of a few planetary radii (which, for very short orbits, can be about as far as the planet’s Roche lobe). Thus, high-energy stellar photons cannot penetrate the deeper atmospheric layers, making XUV heating ineffective.

As the internal heat and the inflation decrease, the contribution from XUV heating increases. Eventually, it overcomes the core-powered mass loss, which soon becomes negligible. Still, the remaining heat can be sufficient for inflating the atmosphere to some extent. In such a case, the XUV heating occurs in an otherwise hydrostatic atmosphere. Yet at higher altitudes it increases the interaction surface $\pi R_{\mathrm{eff}}^{2}$ with stellar photons. The XUV heating is roughly proportional to $R_{\mathrm{eff}}$ to the power of 3 (Watson et al. 1981) or higher (Kubyshkina et al. 2018a), and in the case of inflated atmospheres can be considered as enhanced XUV heating (e.g. Owen and Schlichting 2024).

Atmospheric inflation is further relevant for both non-hydrodynamic thermal and some of non-thermal escape processes, as atmospheric inflation also forces the exobase ($r_{\mathrm{exo}}$) to higher altitudes. For the Jeans-like escape, increasing $r_{\mathrm{exo}}$ leads to the decrease of $\lambda _{\mathrm{exo}}$ and hence, increase of the mass loss (see Eq. (2)). For the non-thermal escape processes, the expansion of the exosphere implies that the interaction surface with stellar XUV and SW increases; it is particularly relevant for photochemical escape, ENA production by SW, and ion pick-up.

## Solar System Observables in the Context of Atmospheric Loss Mechanisms

**Fig. 4:**
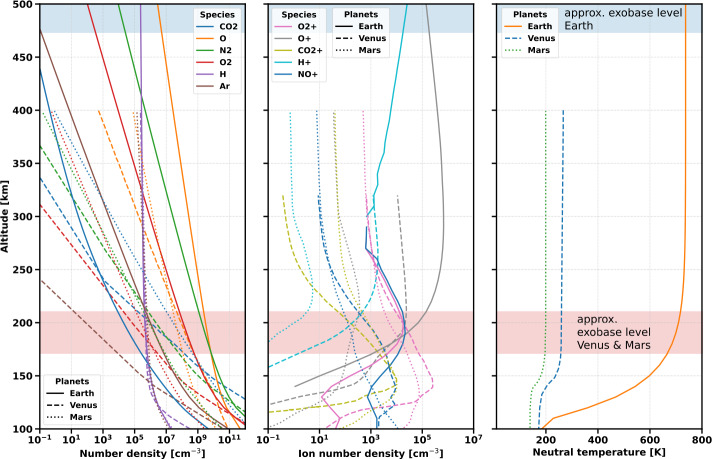
Upper atmospheric profiles for neutrals (left panel), ions (middle panel), and the neutral temperature (right panel) for Earth (solid), Venus (dashed), and Mars (dotted). The profiles for the Earth are from the reference atmosphere model NRLMSIS-2.0 (Picone et al. 2002) and the reference ionosphere model IRI (Bilitza 2001), both for solar minimum (December 02, 2015), except for CO_2_, which was modelled with the 1D upper atmosphere model Kompot (Johnstone et al. 2018) and taken from Scherf et al. (2025). For Venus and Mars, all profiles are based on solar minimum conditions and were taken from Fox and Sung (2001) and Fox and Hać (2009), respectively. We note that for the solar maximum, temperatures and atmospheric densities can be slightly higher for all three planets. The solar activity-dependent exobase levels for Venus, Mars, and Earth are schematically highlighted in the figure

Three of the four terrestrial planets in the solar system, namely, Venus, Earth, and Mars, host a collision-dominated atmosphere. Their surface pressure and atmospheric composition, however, vary significantly. Whereas Venus hosts a CO_2_-dominated atmosphere with almost 100 bar surface pressure, Earth’s atmosphere is dominated by N_2_ and O_2_ with only minor amounts of CO_2_ and has a surface pressure almost two orders of magnitude lower ($\sim 1\text{ bar}$). The Martian atmosphere, dominated by CO_2_, has a surface pressure more than two orders of magnitude lower than that of Earth (i.e., ∼5 mbar). The difference in atmospheric composition between Earth, on the one hand, and Venus and Mars, on the other, implies a substantial difference in their upper atmospheric structures. Figure 4 compares the thermospheric profiles of some neutral (left panel) and ion species (middle panel) on Earth (solid), Venus (dashed), and Mars (dotted), and their neutral temperature profiles.

Earth’s upper atmosphere is relatively hot, reaching temperatures of up to ∼700-1200 K (depending on solar activity, e.g. Picone et al. 2002) in the thermosphere, where the incident XUV surface flux from the Sun is absorbed. Its exobase level can be as high as roughly 500 to 1000 km and both temperature and exobase level depend on the solar activity. The upper atmospheres of Venus and Mars, on the other hand, are compact and cold. Besides the higher molecular weight of their atmospheres compared to the Earth’s, the infrared-coolant CO_2_ efficiently re-emits the incident XUV surface flux back into space, which cools their thermosphere temperatures to about 200 to 250 K and reduces their exobase altitudes to roughly 200 km (see, e.g., Fig. 10 in Way et al. 2023). These are notable differences since Venus experiences twice as much irradiation from the Sun as the Earth, and Mars has a mass that is only a tenth of the Earth’s. Atmospheric composition, therefore, matters for investigating and understanding atmospheric escape processes.

Finally, we note that among the three terrestrial planets with collisional atmospheres, the Earth is the only planet that hosts an intrinsic magnetic field, whereas Venus and Mars do not. This significantly alters the dominant processes responsible for atmospheric escape on these planets (see also Table 2 for a summary on the various non-thermal loss channels). For Venus, atmospheric escape is dominated by ion outflow and ion pickup escape for O and C, and by photochemical escape for H and D. Sputtering of various species constitutes a minor loss channel, whereas thermal escape is negligible for all of the species. In the case of the Earth, ion outflows originating from high-latitude regions such as the polar wind and auroral outflows of its magnetosphere are the dominant process for most of the species (H^+^, He^+^, N^+^, C^+^, O^+^, O^2+^), with H also being lost photochemically. For Mars, O, N, and C are predominantly lost through photochemical processes and via ion escape. Thermal escape, however, is the major loss channel for hydrogen. In addition, Mars is the only planet for which sputtering can be an important driver of escape for various species ranging from the light H to the heavy Ar isotopes. For Titan, photochemical escape and ion escape induced via Saturn’s magnetospheric plasma are at present the most important loss channels for N, H, and CH_4_. Summarizing the above, the major loss channels for Mars, Venus, and Titan are photochemical and ion escape, whereas it is polar outflow for the Earth. For details on the predominant loss channels for each planet, we also refer the interested reader to Steinmeyer et al. (2026, this collection). In the following subsections, we discuss how atmospheric structure, composition, and the presence or absence of an intrinsic magnetic field influence atmospheric loss mechanisms and their observable features.

### Mars: Small, Cool, and Weakly Magnetised Planet with CO_2_ Rich Atmosphere

Presently, Mars has no global intrinsic magnetic field, and the solar wind interacts directly with the upper atmosphere. As introduced in Sect. 2.2, in addition to the thermal (Jeans) escape, there are four major atmospheric escape mechanisms, i.e., photochemical escape, ion pickup, atmospheric sputtering, and cold ionospheric outflows (Fig. 2 (b) and Table 2). In the past decade, there have been significant advances in observations of atmospheric escape from Mars based on Mars Express and Mars Atmosphere and Volatile Evolution (MAVEN) spacecraft observations (e.g. Jakosky et al. 2018; Nilsson et al. 2023, and references therein). Below, we summarize how each mechanism is identified by observations.

The mass loss rate by Jeans escape can be estimated from observations of the density and temperature of neutral species at the exobase. In-situ observations of the altitude profile of neutral densities have been used to determine the exobase height (e.g. Jakosky et al. 2017).

Concerning photochemical escape, the direct measurement of the suprathermal component of the neutral atmosphere at high altitudes is often challenging, although important for estimating the photochemical escape rate and the exosphere (especially neutral corona). Therefore, exosphere observations have been primarily conducted by using optical instruments, such as UV spectrographs (e.g. Chaffin et al. 2018; Chirakkil et al. 2024). Lillis et al. (2017) observationally estimated the oxygen photochemical escape rate by combining in-situ density observations of electrons, ions, and neutrals. They found a power law exponent of 2.6 for the EUV dependence of the photochemical escape rate. However, such an exponential dependence was not confirmed by other studies (Zhao and Tian 2015; Zhao et al. 2017; Amerstorfer et al. 2017; Dong et al. 2018; Scherf and Lammer 2021) for larger EUV fluxes, as two competing mechanisms influence the photochemical escape rates. Whereas an increase in the EUV flux tends to increase the photochemical production of suprathermal O (and C) atoms, the accompanied EUV-induced expansion of the upper atmosphere above the main photochemical production zone leads to more collisions, and hence to larger thermalization rates (Amerstorfer et al. 2017; Lichtenegger et al. 2022). Although photochemical losses still increase toward larger EUV fluxes, this effect reduces photochemical escape rates compared to the exponential assumption by orders of magnitude for EUV fluxes ≳10 times the Sun’s present-day flux (see also Fig. 4a in Lichtenegger et al. 2022).

**Fig. 5:**
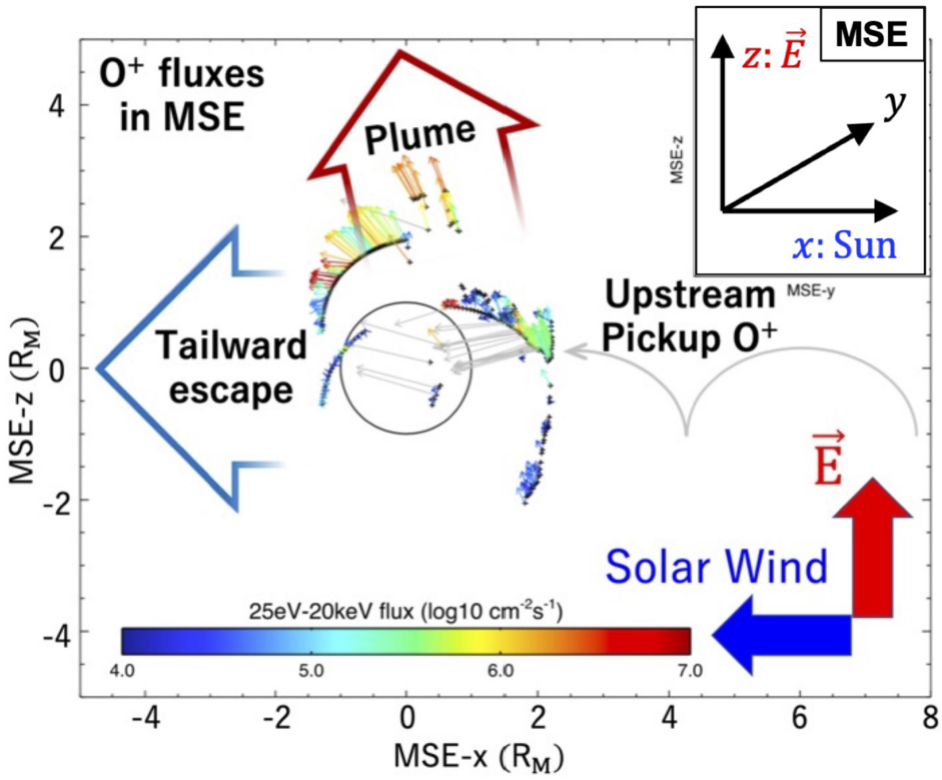
Two major ion escape channels from Mars observed by MAVEN, i.e., polar plume (red empty arrow) and tailward escape (blue empty arrow), in MSE (Mars-centered Solar Electric) coordinates (modified from Dong et al. 2015)

**Fig. 6:**
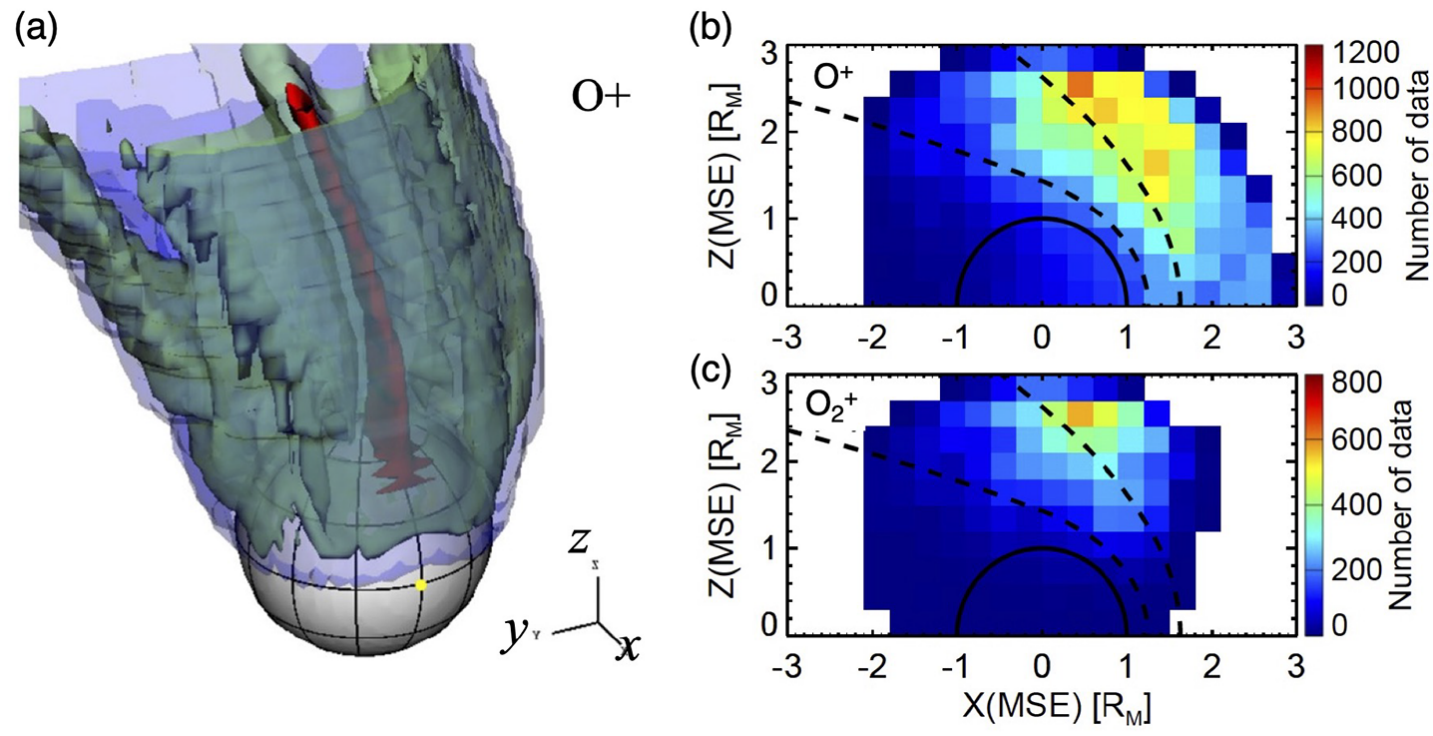
(a) Spatial distribution of the O^+^ flux of polar plumes originating from the ionosphere. (b) Observed spatial distributions of O^+^ and (c) $\mathrm{O}_{2}^{+}$ polar plumes in the MSE coordinates (modified from Sakakura et al. 2022)

Significant part of the escape from Mars occurs through ion pickup. Observationally, there are two major escape channels of ions, i.e., polar plumes and tailward flows. The polar plume is a permanent plasma structure in the Mars-centered Solar Electric (MSE) coordinates, with the x-axis pointing to the Sun, the z-axis in the direction of the upstream solar wind convection electric field, and the y-axis completing a right-handed system (Dong et al. 2015). As shown with the red empty arrow in Fig. 5 the polar plume is the planetary ion flow with a significant velocity component along the solar wind electric field direction. The O^+^ plume includes a significant contribution from the ion pickup from the oxygen corona. Since the solar wind electric field can penetrate the ionosphere, acceleration by the electric field can occur down to the penetration altitude. The polar plume also includes the bulk outflow of ionospheric ions toward the electric field direction as shown in Fig. 6 (a). It should be noted that polar plumes of molecular ions such as $\mathrm{O}_{2}^{+}$ and $\mathrm{CO}_{2}^{+}$ have also been observed and their spatial distributions are more confined than that of O^+^ ions, since they originate only from the ionosphere (no source in the corona) as shown in Fig. 6 (b) and Fig. 6 (c) (Sakakura et al. 2022).

Since the spatial distribution of the pickup ions strongly depends on the location in the MSE coordinates, the ambiguity in the MSE coordinate determination and limited spatial coverage make it difficult to derive an accurate total escape rate via ion pickup. One of the solutions is to estimate the rate from the source corona observations. The total escape rate due to ion pickup originating from the corona has also been estimated from a corona retrieval based on observations of the ring-shaped distributions formed by pickup ions (e.g. Masunaga et al. 2024, and references therein).

Escape by atmospheric sputtering has been estimated based on observations of high-energy ion precipitation onto the atmosphere. Using the observed energy distributions of the precipitating ions, the exospheric structure induced by the precipitation needs to be modelled to estimate the expected escape rate (e.g. Leblanc et al. 2018, and references therein). For present-day Mars, the contribution of atmospheric sputtering is negligible for heavy ions. However, it could have been significant in ancient times, when the solar wind flux and XUV radiation were much stronger than the present day values (e.g. Luhmann and Kozyra 1991).

**Fig. 7:**
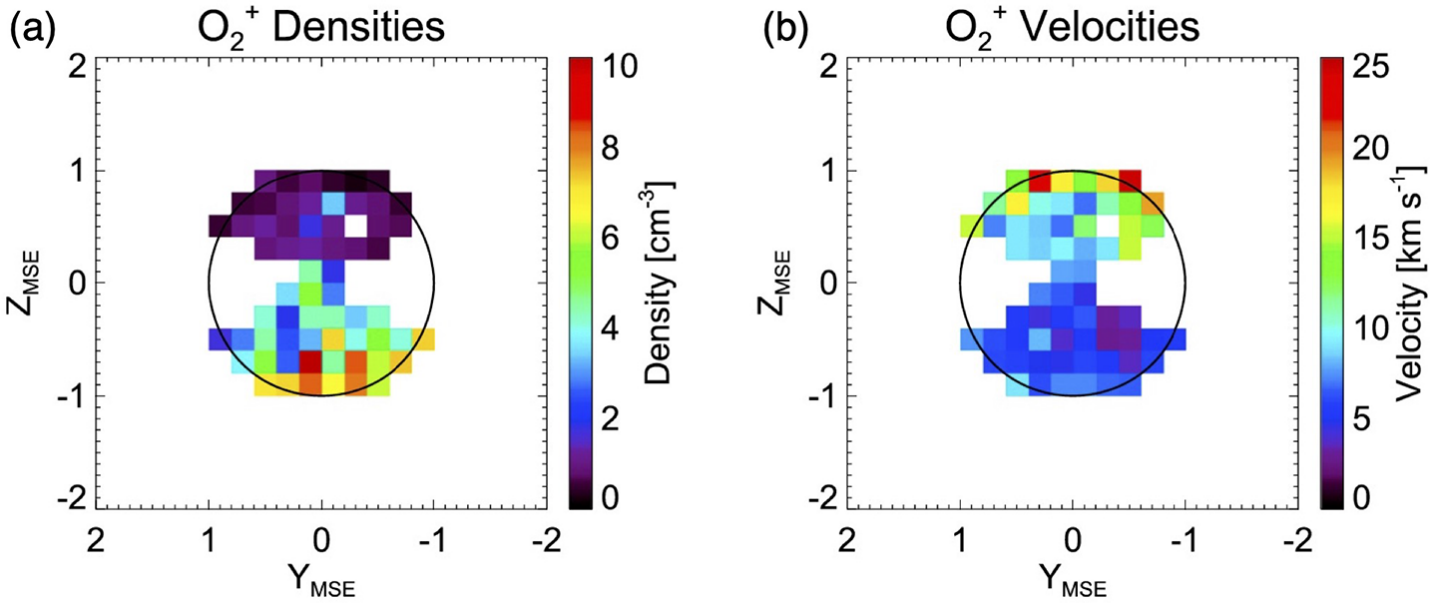
Spatial distribution of $\mathrm{O}_{2}^{+}$ density (a) and velocity (b) in the Martian induced magnetotail observed by MAVEN. Flux of polar plumes originated from the ionosphere in MSE coordinates (Sakakura et al. 2022)

**Fig. 8:**
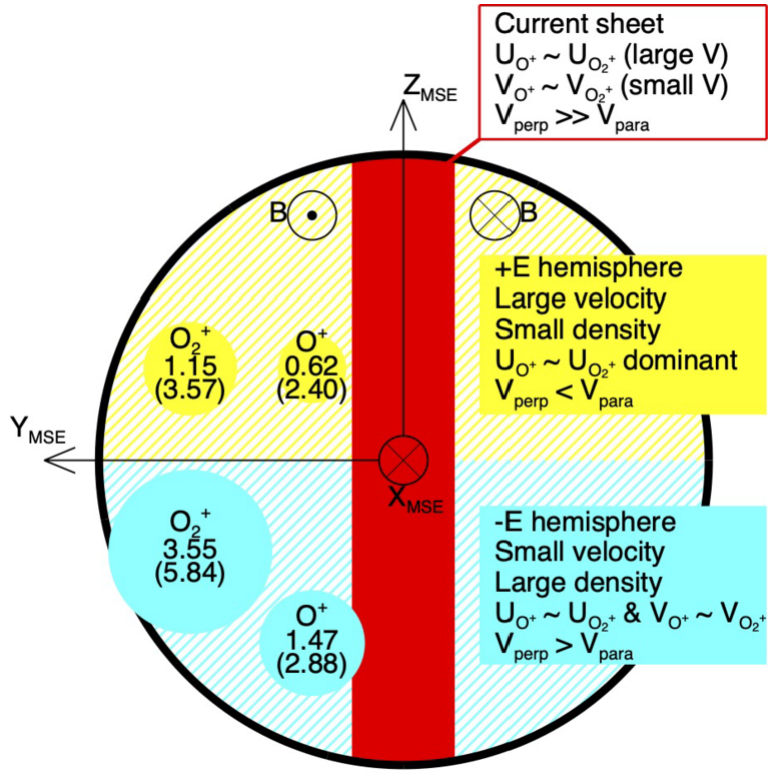
Summary of plasma characteristics observed in the magnetotail, which are important to identify relevant plasma processes to the ion acceleration and heating adopted from Inui et al. (2019)

The cold ionospheric outflow is also an important contributor to the ion escape. The Martian induced magnetotail is filled with planetary cold ions often flowing in the anti-sunward direction (e.g. Nilsson et al. 2012; Inui et al. 2019). This tailward escape, shown with a blue empty arrow in Fig. 5, observationally includes the most of ionospheric outflows. As introduced in Sect. 2.2, the cold ionospheric outflows can be caused by various plasma processes. In-situ observations of planetary ions are essential to identify the specific acceleration and heating processes causing their escape. Observations have revealed systematic structures in the ionospheric outflows in the magnetotail: evolution of heavy ion distribution functions with distance from Mars show the gradual continuous acceleration of the ions flowing tailward in the current sheet (Nilsson et al. 2012). As shown in Fig. 7, the velocity (density) is higher in the +E (-E) hemisphere than the -E (+E) in the MSE coordinates. As summarized in Fig. 8, observations suggest that major acceleration/heating mechanisms causing the tailward ion flows differ depending on the location of the induced magnetotail. Detailed classification can be found in the literature (e.g. Inui et al. 2019, and references therein). In addition to the identification of the escape mechanisms, estimation of the total escape rate based on observations has been intensely studied (e.g. Jakosky et al. 2018; Ramstad and Barabash 2021).

### Venus: Hot Earth-Size Planet with Induced Magnetosphere and CO_2_ Rich Atmosphere

Currently, Venus has a thick CO_2_-dominated atmosphere and lacks a strong intrinsic magnetic dipole field. Therefore, the interaction with the solar wind creates an induced magnetosphere, similar to that of Mars as described above. The interaction with the solar wind is the main cause of atmospheric escape from Venus today (e.g., Lammer et al. 2006; Gillmann et al. 2022), along with the photochemical escape of hydrogen (with escape rates $>10^{25}\text{ s}^{-1}$ (Lammer et al. 2006)). Starting at present-day Venus, we can first elaborate on the presence of the atmospheric escape channels described in the previous sections. A full review of the escape processes at Venus and its potential consequences for the Venusian atmospheric evolution is provided by (Gillmann et al. 2022).

The thermal escape at Venus today is minuscule, as a consequence of two combined factors. Firstly, the temperature of its exosphere is only around 270 K (Limaye et al. 2017), cooled by the infrared emissions from CO_2_, which stands in stark contrast to its extremely hot surface temperature of ∼735 K. Secondly, the escape velocity at the exobase is around 10 km s^−1^, which is significantly higher than at Mars. This leads to only a tiny portion of the assumed Maxwellian distribution at the exobase being above the escape velocity that can escape the atmosphere. Therefore, thermal escape can only account for a very small percentage of the total escape rate today (e.g., Lammer et al. 2006). Hydrodynamic escape is not currently taking place at Venus but is considered to have been an important factor during the runaway greenhouse phase earlier in Venus’ history (Ingersoll 1969).

Therefore, non-thermal escape processes are the most important channels for the atmospheric escape at Venus today. One should note that the processes that dominate at Venus are different from those at Mars. This may in part be explained by the difference in mass between the two planets, hence, in their escape velocities.

At Venus, photochemical escape is only important for lighter species, such as hydrogen, and can be considered negligible for heavier species, such as O and O_2_ (Lammer et al. 2006). Sputtering, similarly to Mars, has never been directly measured at Venus and can only be estimated through modelling and indirect measurements of related properties. Estimates of the sputtering rate at Venus contribute less than 30% to total atmospheric escape (Luhmann and Kozyra 1991; Lammer et al. 2006). Consequently, except for H, the main escape occurs in the form of ion escape caused by the direct interaction between the solar wind and the Venusian ionosphere. Here several processes are important, as elaborated upon in Sect. 2.2.2.

Similarly to Mars, an ion pickup plume forms in the direction of the solar wind motional electric field, which carries with it an estimated 30% of the total escaping ions from Venus (Masunaga et al. 2019). Due to the larger mass of Venus compared to Mars, and the higher interplanetary magnetic field strength around the orbit of Venus, the ion pickup plume is less spectacular in its shape than Mars’. The smaller ion gyroradius in relation to the planetary mass causes the pickup plume to form closer to Venus than Mars, where it follows along the stream of the shocked solar wind plasma as it is diverted around the induced magnetosphere (c.f. Figure 6 & 9 in Jarvinen et al. 2016).

Besides the loss of suprathermal H, the largest channel of ion escape today occurs in the induced magnetotail of Venus, where a plethora of escape mechanisms contribute to the total escape, ranging from ion cloud formations (e.g. Brace et al. 1982), plasma sheet acceleration (e.g. Barabash et al. 2007), electric field acceleration (Collinson et al. 2016), etc. (see Sect. 2.2.2). The estimated total ion escape rates from Venus today lie in the range of $(3\mathrm{-}6)\times 10 ^{24}\text{ s}^{-1}$ (Futaana et al. 2017). However, it has been shown to vary depending on solar cycle and upstream conditions (e.g. Persson et al. 2018, 2020; Masunaga et al. 2019), including extreme conditions such as Interplanetary Coronal Mass Ejections (ICMEs) and Corotation Interaction Regions (CIRs) (e.g. Luhmann et al. 2007; Edberg et al. 2011; McEnulty et al. 2010).

As indicated above, the presence of these different escape channels at Venus is dependent on the current conditions at Venus. Other work (e.g. Way and Del Genio 2020) has shown that Venus’ atmospheric composition and density could have been drastically different at various times in its past. If we neglect the post-accretion magma ocean atmosphere, and assume a path to temperate conditions like that of early Earth, the early Venusian atmosphere was likely N_2_–CO_2_ dominated, as it is also suggested for the late-Hadean/early-Archean Earth (e.g. Charnay et al. 2017). In that case, the atmosphere had a substantially lower surface pressure, presumably around a few 100 mbar to several bar, compared to the current 92 bar surface pressure, with CO_2_ partial pressures (PPs) ranging from roughly 100% (like today) to less than 20% in an otherwise N_2_ dominated atmosphere. In such scenarios, smaller CO_2_ PPs alongside higher N_2_ PPs would influence exospheric composition, temperature, and height with corresponding consequences for escape (see Sect. 6.1).

Note that a lower CO_2_ PP in the Venusian atmosphere would decrease the radiative cooling, which consequently increases the exospheric temperature and causes an expansion of the upper atmosphere. How much the exospheric temperatures, and subsequent exospheric expansion are affected depends on the interplay between the chemical heating/cooling and the radiative cooling in the atmosphere (for more details see Gronoff et al. 2020). Johnstone et al. (2018) showed that increasing the CO_2_ mixing ratio of Earth’s atmosphere decreases the exobase altitude and temperature from today’s value. They found that a mixing ratio of around 30%, which is approximately a factor of three lower than the PP in Venus’ today atmosphere today, causes a decrease of the exobase temperature to around 300 K and an altitude of less than 200 km, which is not far from the current exobase at Venus of around 270 K and 180 km. However, when decreased to less than 10% Johnstone et al. (2018) showed that both the temperature and the altitude of the exobase increased by a factor of two from a Venusian-like mixing ratio atmosphere.

In such scenarios, we may expect several things to take effect. An increase in exospheric temperatures would lead to an increase in Jeans escape (Johnstone et al. 2018), which in combination with a higher XUV flux from the Sun at about 4.4-4.5 Gyrs ago (Tu et al. 2015) could result in hydrodynamic escape that would cause a significant portion of the atmosphere to be lost (e.g. Lammer et al. 2006; Tian et al. 2008a; Johnstone et al. 2021b). An expansion of the atmosphere would also lead to a larger interaction area between Venus and the Sun, affecting the size and structure of the induced magnetosphere. A larger interaction area leads to an increase of energy and momentum transfer to the atmosphere and higher ion escape rates (Persson et al. 2021; Gronoff et al. 2020). However, the induced magnetosphere boundary does not linearly increase in size with an increased exobase altitude as it is controlled by the pressure balance between the ionospheric thermal pressure and the solar wind dynamic pressure (Futaana et al. 2017). One should also account for the different properties of the wind at young ages, it is expected to be denser and hotter (e.g. Vidotto 2021). An increase in the size of the induced magnetosphere that is smaller in relation to the inflation of the exosphere would lead to an increased portion of the expanded atmosphere ending up outside of the induced magnetosphere boundary, which could also lead to a significant increase in atmospheric loss due to an increase in charge exchange, ion pickup, and sputtering (e.g. Gronoff et al. 2020; Kulikov et al. 2006). A change in the atmospheric composition may also affect the conductivity of the ionosphere if it is combined with a change of the ionospheric composition and structure, which will have further effects on the structure of the induced magnetosphere (Cravens et al. 1980).

Additionally, the configuration of the Interplanetary Magnetic Field (IMF) plays an important role for unmagnetized planets such as Venus and Mars. For example, it was recently shown that an IMF that is aligned with the solar wind flow will cause the induced magnetospheres to degenerate, and will affect the total ion escape from the planet (e.g. Fowler et al. 2022; Zhang et al. 2024). Conditions for the flow-aligned IMF may have been more common during the earlier stages of our solar system when the Sun had a faster rotation rate (e.g. Tu et al. 2015). This may be more common for exoplanets that lie closer to their host stars than Venus in which the aberrated solar wind is more inclined to be aligned with the nominal Parker Spiral angle of the star’s magnetic field (Zhang et al. 2024).

Another important factor for Venus is the question of an intrinsic magnetic field. It is not known if Venus ever had an intrinsic magnetic field (O’Rourke et al. 2018). If it did it would have changed the interaction with the solar wind significantly and made it more Earth-like (which is further described in Sect. 3.3 below).

All these scenarios must also take into consideration the increased solar XUV radiation levels in the past (e.g. Tu et al. 2015). In combination with any atmospheric composition changes, this would lead to additional heating effects on the Venusian upper atmosphere and its atmospheric escape (e.g. Johnstone et al. 2021b). Therefore, it is fair to say that it is not straightforward to extrapolate escape rates observed in the present day backwards in time to understand the past Venusian atmosphere and its atmospheric escape.

### Earth: Moderate HZ Planet with Intrinsic Magnetosphere and N_2_–O_2_ Dominated Atmosphere

**Fig. 9:**
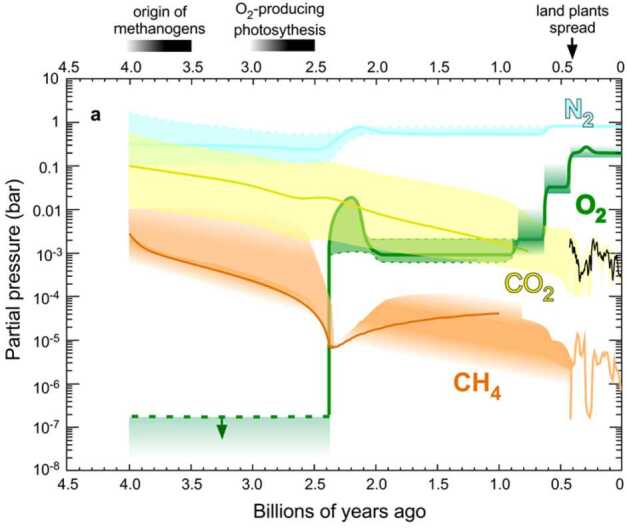
A synopsis of the evolution of the terrestrial atmosphere during the last 4 billion years. From Catling and Zahnle (2020)

The present composition of the terrestrial atmosphere is quite different from that of Mars and Venus, though the initial atmospheric compositions of these three planets are believed to have been similar (Lammer et al. 2018, 2021). The long-term development of the Earth’s atmosphere is shaped by its interactions with the planet’s interior, surface, living organisms (hence, it is highly connected to the development of habitable conditions), incoming material from space such as meteoroids, and the loss of atmospheric particles into space (Yamauchi and Wahlund 2007; Lammer et al. 2008a, 2020b; Avice and Marty 2020; Gronoff et al. 2020). The present atmospheric composition is thus quite different from that of the primordial Earth (Stüeken et al. 2020; Catling and Zahnle 2020), compare Fig. 9.

Given the value of the gravitational escape velocity from our planet (11.2 km s^−1^), present-day (kinetic) thermal escape by means of neutral atoms or molecules is mostly limited to hydrogen. All other heavier elements, as for example oxygen and nitrogen, have to go through a series of acceleration mechanisms in order to attain escape velocities. These elements, which presently represent over 99% of the mass of the terrestrial atmosphere, need thus to be first ionised (see Sect. 2.2).

Although the magnetosphere of the Earth deflects most of the solar wind flow away from the upper atmosphere operating as a shield, at the same time it increases the cross-sectional area between the solar wind and the Earth by a factor of more than 200. Hence the terrestrial magnetic field increases the amount of solar wind kinetic energy that is intercepted by the magnetosphere and which can then be channelled down to the high-latitude ionosphere, a portion of it ending up in driving ion outflow and acceleration (Li et al. 2017; Gunell et al. 2018; Maggiolo et al. 2022). At the equatorial latitudes the trapped cold plasma population of the plasmasphere (Fig. 3) constitutes another source of plasma transport to the outer magnetosphere, which can then eventually escape to the interplanetary medium (Lemaire 2001; Dandouras 2013; Borovsky et al. 2014).

**Fig. 10:**
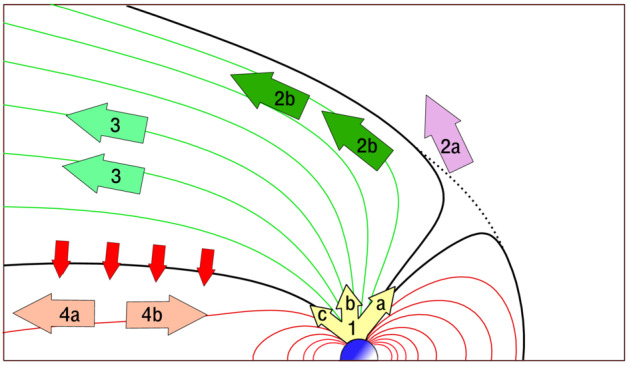
Schematic of the terrestrial magnetosphere (meridian cut, Sun is on the right) showing the three main source regions of outflowing ions from the ionosphere (yellow arrows) and their pathways into the magnetosphere (purple, green, red and beige arrows). 1a, 1b and 1c relate to the cusp, polar wind and nightside auroral zone outflow respectively (see text). The red and green curved lines represent in their turn the closed and open magnetic field lines. Upwelling ions can either directly escape through the cusp (2a), or along the plasma mantle (2b). Polar wind ions get into the magnetotail lobes (3), where magnetospheric convection (red arrows) can bring them into the plasma sheet. There, they can be either injected tailward (4a) or Earthward (4b). From Slapak and Nilsson (2018)

The ions upwelling from the high-latitude ionosphere can originate either from (see Fig. 10)

- The polar cap, which is connected to the open magnetic field lines and where ions are extracted from the ionosphere by the ambipolar electric field and then form the polar wind (e.g. Schunk 2000), or
- The funnel-shaped polar cusp and cleft, where the ions are accelerated upwards following multiple step processes (André et al. 1990), or
- The auroral zone, where energy is provided by the energetic electron precipitation originating from the magnetosphere (Yau and Andre 1997).

**Fig. 11:**
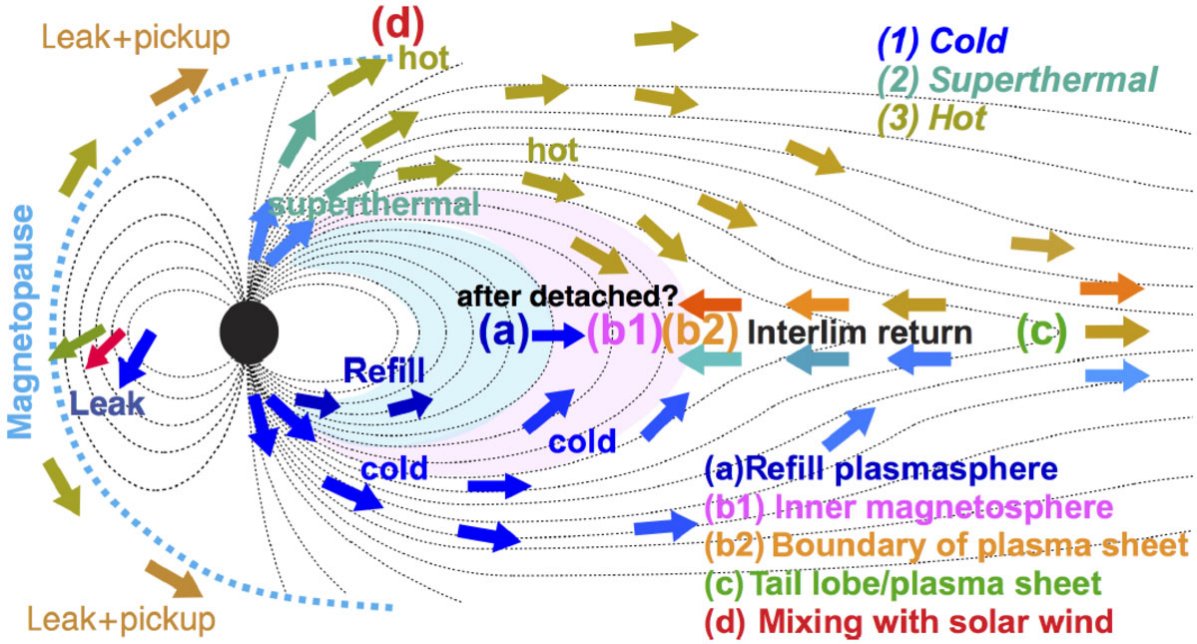
Simplified diagram showing the various transport routes in the magnetospheres for the ions originating from the terrestrial ionosphere. From Yamauchi (2019)

Depending on the original location of the outflowing ions in the ionosphere, the type of ion species, the magnetospheric convection, and the conditions of interplanetary magnetic field, these ions, as shown schematically in Fig. 11, can have very different trajectories in the magnetosphere (Yamauchi 2019). The ions outflowing from the high-latitude ionosphere generally follow tailward bended trajectories. They can then get out of the magnetosphere through the cusp, or can move along the plasma mantle, or can get transported anti-Earthward along the open field lines of the magnetotail lobes, or from the lobes they can eventually get into the plasma sheet. There, depending on the local magnetic configuration and the state of the magnetospheric convection, they can be re-injected into the inner magnetosphere, or can directly escape tailwards (Ebihara et al. 2006; Haaland et al. 2012; Chappell 2015; Yamauchi 2019; Dandouras 2021). We note that, even for the ions injected into the inner magnetosphere, a large number will finally escape in the form of energetic neutral atoms, following charge-exchange collisions with the neutral hydrogen of the exosphere.

Earth’s ionosphere and magnetosphere have been studied in-situ or by remote sensing techniques by several missions, providing information on the ionospheric source, the transport and acceleration through the magnetosphere, and eventual leakage of these particles to the interplanetary medium (e.g. Chappell 2015; Dandouras et al. 2020). Some of the most prominent results are

**Fig. 12:**
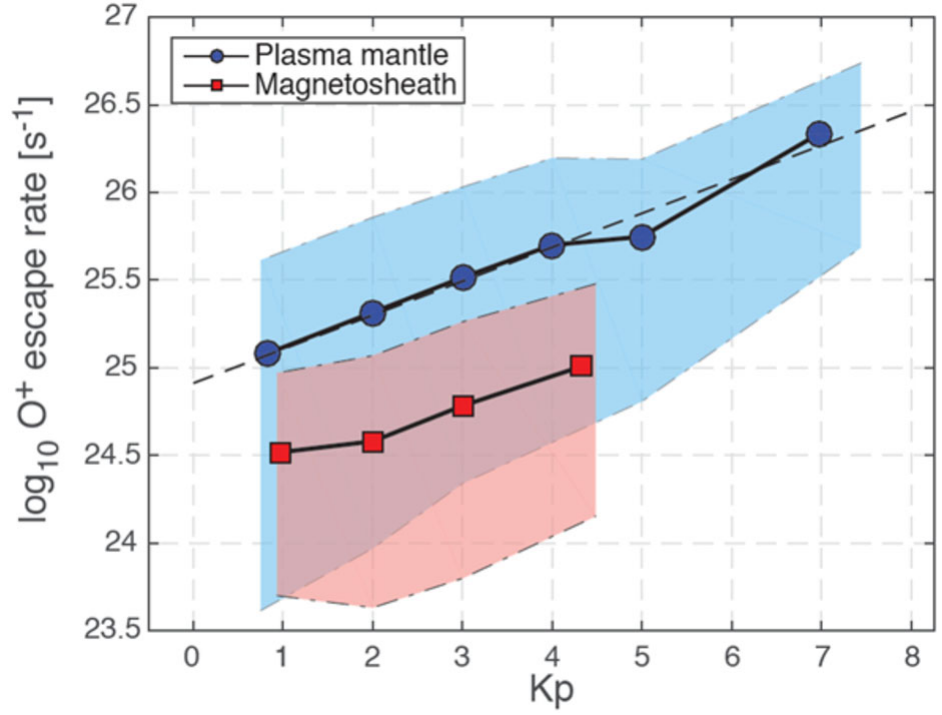
Observed escape rates of ${O^{+}}$ (ions s^−1^) through the plasma mantle (blue circles) and through the dayside magnetosheath (red squares) as a function of the geomagnetic activity index Kp (which quantifies disturbances in the horizontal component of Earth’s magnetic field with an integer in the range 0–9, with 1 being calm and 5 or more indicating a geomagnetic storm). From (Slapak et al. 2017, and Corrigendum)
- The total loss of heavy ions during the last four billion years, considering the sensitivity of the outflow on the geomagnetic and solar activity conditions and the highly active Sun in its early stages (Güdel 2020), could be roughly equivalent to 40% of the current atmospheric oxygen content (Slapak et al. 2017; Kislyakova et al. 2020);
- Plasmaspheric outflows, in the form of plasmaspheric plumes (plasma elements detached from the plasmasphere and propagating outwards) and plasmaspheric wind (continuous slow outwards plasma transport), constitute the main ion outflow mechanisms from the equatorial latitudes (Dandouras 2013; Borovsky et al. 2014). Similar outflows should also exist around other planets, quickly rotating and having an ionised atmosphere and an intrinsic magnetic field.

- Low-energy (few eV) ions coming from the polar caps fill the lobes of the magnetotail almost continuously. This upflow is sensitive to the Solar EUV intensity and can show a twofold growth at the solar cycle maximum (Engwall et al. 2009; André et al. 2015);
- The cusps, where energy supplied from the solar wind is concentrated due to the funnel-shaped local magnetic field geometry, are the main source outflowing high-energy ions (mostly ${O^{+}}$ and ${N^{+}}$, but also some molecular ions ${N_{2}^{+}}$, ${NO^{+}}$, ${O_{2}^{+}}$; see Kistler et al. 2010; Kronberg et al. 2014; Yamauchi et al. 2024);
- The energetic (∼100 eV to several keV) heavy-ion outflow is closely tied to the solar wind parameters. A growth in the outflow by a factor of up to ∼100 has been observed as a function of the dynamic pressure of the solar wind (Schillings et al. 2019);
- The energetic heavy-ion outflow is also highly dependent on the geomagnetic activity, as expressed by the Kp activity index, and follows a logarithmic trend (Fig. 12). At peak activity levels, the escape rate of heavy ions is almost 100 times as high as in normal conditions (Slapak et al. 2017);

However, there are issues concerning the outflow and escape of terrestrial ions that have to be addressed. The most important of them concerns the accurate composition of the escaping ion populations and the way it responds to the external conditions, as the solar and geomagnetic activity conditions (Dandouras 2021; Yamauchi et al. 2024). Most of the ion mass spectrometers flown in the magnetosphere and operating in the few keV energy range do not have enough resolution to separate the nitrogen ions from the oxygen ions (Rème et al. 2001; Young et al. 2016).

## Role of Stellar Input

Different physical processes ruled by the host star contribute to the energy input to a planetary atmosphere according to the planetary orbit, the stellar parameters, and the atmosphere’s chemical composition.

### Stellar Magnetic Activity and High-Energy Radiation

In late-type stars with an internal structure similar to our Sun, we observe non-radiatively heated outer stellar atmospheres showing spatial and temporal variability owing to the complex geometry and evolution of stellar magnetic fields (e.g., Schrijver et al. 2019). The X-ray and EUV (XUV) radiation comes from the corona, with temperatures $T$ of the order of $10^{6}\text{ K}$, and from the thin transition region between the chromosphere ($T \sim 10^{4}\text{ K}$) and the corona itself. The coronal plasma is mainly responsible for the emission below $\sim 45\text{ nm}$, dominated by He II, Mg X, and highly ionized Fe lines in the Sun. In contrast, at longer wavelengths, the spectrum is dominated by the He I continuum and the hydrogen Lyman continuum up to the hydrogen ionization threshold at 91.2 nm. Beyond that limit, the transition region lines emitted by O III, O VI, Si IV, N V are the most prominent features over a very low continuum, up to the 121.5 nm of the strong Ly-$\alpha $ line (Linsky 2019; Linsky and Redfield 2024). Lower energy chromospheric lines, for example, C II at 133.4 nm (France et al. 2018), are usually not as important as the XUV spectrum.

The part of the XUV spectrum most relevant for atmospheric escape depends mainly on the planetary atmosphere’s chemical composition. In a primordial atmosphere dominated by hydrogen and helium, the radiation below 91.2 nm is responsible for photoionisation heating of H and He (see Sect. 2.1.3). On the other hand, in the case of secondary atmospheres containing molecules such as CO_2_, H_2_O, N_2_, CO, and O_2_, photons with wavelengths longer than $\sim 80\text{ nm}$ are the most relevant for their photodissociation. The strong chromospheric Ly-$\alpha $ line is especially important because it photodissociates water and methane (cf. Fig. 10 of Linsky and Redfield 2024).

The XUV spectrum of late-type stars depends on their magnetic fields, produced by the stellar hydromagnetic dynamo. X-ray emission is well correlated with the Rossby number $R{o}$, that is, the ratio of the stellar rotation period $P_{rot}$ to the convective turnover time $\tau _{c}$. This characterizes internal stellar convection and is estimated by means of stellar models (e.g. Spada et al. 2013), $R{o} \equiv P_{rot}/\tau _{c}$. The best correlation between X-ray emission and stellar rotation is obtained by considering the ratio of the stellar X-ray luminosity to its bolometric luminosity, $R_{X} \equiv L_{X}/L_{\mathrm{bol}}$. This can be expressed as a broken power law with the exponent decreasing below a critical value of $Ro^{sat}$, that indicates saturation of the stellar coronal emission in the most rapid rotators (e.g. Wright et al. 2011; Johnstone et al. 2021a)

9 $$ R_{\mathrm{X}} = \textstyle\begin{cases} R_{\mathrm{X}}^{\mathrm{sat}}, &\text{if } R{\mathrm{o}}\leq R{\mathrm{o}}^{ \mathrm{sat}}, \\ CR{\mathrm{o}}^{\beta},& \text{if } R{\mathrm{o}} > R{\mathrm{o}}^{\mathrm{sat}}, \end{cases} $$

where parameters C, $\beta $, $R{o}^{sat}$, and $R_{X}^{sat}$ can be defined empirically.

The greatest advantage of the $R_{X}-R{o}$ empirical correlation is that it is valid for stars of all spectral types between F and M, independently of the stellar intrinsic luminosity, due to the $L_{\mathrm{bol}}$ normalization (e.g. Wright et al. 2011; Jackson et al. 2012). Similarly, the Rossby number threshold for $R_{X}$ saturation is independent of the spectral type and the rotation period. Main-sequence stars with a mass smaller than $\sim 0.35$ M_⊙_ are considered fully convective. The dependence of $R_{X}$ on the Rossby number is generally regarded as similar to that established for more massive stars (cf. Sect. 3.1 of Johnstone et al. 2021a), although some very-low-mass stars, such as Trappist-1 ($M \sim 0.09$ M_⊙_), show a significantly lower XUV luminosity than predicted by that relationship (Wheatley et al. 2017). However, the present-day XUV surface flux ($F_{\mathrm{XUV}}$) in the habitable zone (HZ) of Trappist-1 is still roughly two orders of magnitude higher than the present-day flux at Earth (e.g., Wheatley et al. 2017; Birky et al. 2021).

The intrinsic variability of the X-ray emission on timescales from minutes to decades produces a remarkable dispersion around the mean $R_{X}-R{o}$ correlation. Nevertheless, it can still be used to estimate the total flux received by a planet over evolutionary timescales (Johnstone et al. 2021a), as they are much longer than those characteristic of stellar variability. The estimate of the total flux received in the EUV bandpass is uncertain within at least a factor of two (France et al. 2018) due to strong absorption by the interstellar medium between the ionization threshold of hydrogen at 91.2 nm and $\sim 35\text{ nm}$. Therefore, all parametrizations of the stellar EUV flux as a function of the Rossby number or of the stellar age (e.g., Sanz-Forcada et al. 2011; Johnstone et al. 2021a; King et al. 2018) are based on indirect methods (France et al. 2018; Linsky 2019; Linsky and Redfield 2024). Similarly, the Ly-$\alpha $ flux is strongly absorbed by interstellar hydrogen, thus empirical correlations are based on a reconstruction of the line profile starting from its extended wings. These limitations must be considered when simulating the evolution of planetary atmospheres.

A cumulative effect by stellar flares is included in the $R_{X}-R{o}$ correlation because they are a kind of short-term variability (Johnstone et al. 2021a). However, the temporary intensification of the short-wavelength continuum and spectral line radiation by up to two orders of magnitude can be relevant for planetary atmospheric escape because it increases the exobase radius remarkably, thus enhancing atmospheric mass loss rates (do Amaral et al. 2022). Moreover, flares are often accompanied by slower propagating CMEs (though the latter are not necessarily directed in a way that allows interaction with the planet) which can interact with already “excited” atmospheres (e.g. Hazra et al. 2025).

### Co-Evolution of Stellar Rotation and High-Energy Radiation

Late-type stars are born with a wide range of rotation rates: $\Omega $ between $\sim 1$ and $\sim 50$ $\Omega _{\odot}$. Their rotation stays constant during the pre-main-sequence phase, as long as they are dynamically locked to their circumstellar accretion discs. This phase is short – the mean disc lifetime is of the order of a few Myr (e.g. Mamajek 2009) – and is followed by a phase of contraction, while the star moves towards the zero-age main sequence (ZAMS). This leads to a rapid acceleration of the rotation owing to the reduction of the moment of inertia, making $\Omega $ maximal on the ZAMS. Afterwards, the effect of the stellar magnetized wind leads to a steady decrease of the stellar angular velocity, until all the stars of spectral types F, G, and early K converge towards the same evolutionary sequence where $\Omega $ is roughly proportional to $t^{-1/2}$. Where $t$ is the age of the star, a dependence called the Skumanich law (after Skumanich 1972). Such a convergence of the rotational evolution tracks takes about 0.6 Gyr for F and G stars, and longer for later spectral types. It takes Gyrs for M dwarfs, which exhibit a wide range of rotation periods even in clusters of 2.5 Gyr of age or older.

**Fig. 13:**
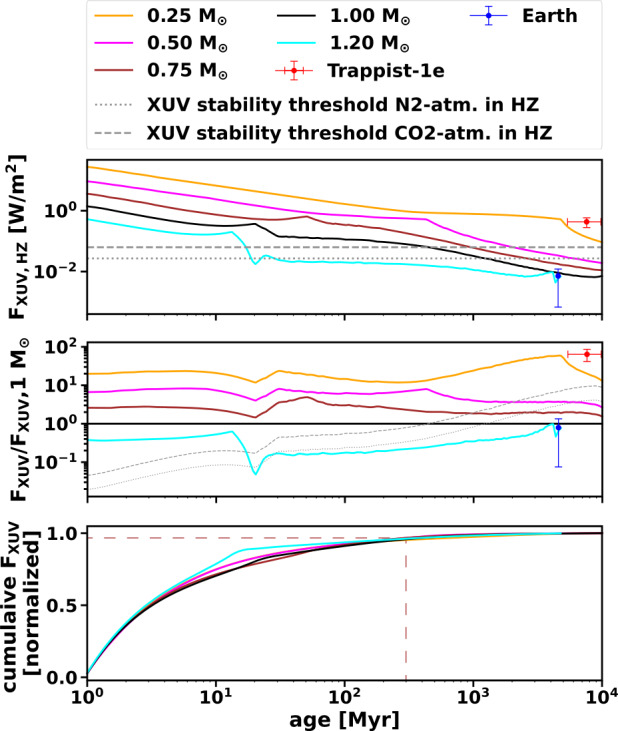
The $F_{\mathrm{XUV}}$ evolution of 0.25-1.2 $M_{\odot}$ stars according to the model of Johnstone et al. (2021a) (solid lines). The upper panel shows the $F_{\mathrm{XUV}}$ evolution of moderate rotators of the given masses in the middle of their respective HZ. The middle panel shows the same, but normalized to a moderately rotating Sun-like star (the black line in both panels). The lower panel shows the cumulative flux of moderate rotators normalized to 1 and highlights an age of 300 Myr (dashed lines) at which >95% of the cumulative flux is already emitted for all evolutionary tracks. The red dot with error bars shows $F_{\mathrm{XUV}}$ at Trappist-1 e (from Wheatley et al. 2017) (whose host star Trappist-1 has a mass of ∼0.09$M_{\odot}$) and the blue dot shows $F_{\mathrm{XUV}}$ for the Earth (based on Tu et al. 2015). The dashed and dotted grey lines illustrate the thermal stability threshold of secondary CO_2_-dominated (99% CO_2_, 1% N_2_) and N_2_-dominated (90% N_2_, 10% CO_2_) atmospheres in the HZ according to Van Looveren et al. (2024); see also Scherf et al. (2024)

Since the convective turnover time $\tau _{c}$ stays almost constant during the main-sequence evolution, the XUV fluxes of those stars steadily decrease with age and the decrease speeds up after a star leaves the saturation regime. It turns out that, even though most of the cumulative XUV flux received by their planets is likely to be contributed after the saturated phase during the first 0.3-0.5 Gyr on the main sequence, as illustrated in the bottom panel of Fig. 13 (see Johnstone et al. 2021a, for details), the XUV flux becomes the dominant contributor to atmospheric evaporation starting from a few tens of Myr of evolution and then remains dominant on Gyr timescales (King and Wheatley 2021). Stars having short-lived discs on the pre-main sequence can reach the ZAMS with a rotation rate exceeding 50 $\Omega _{\odot}$ and are characterized by a much higher level of XUV radiation than slower ZAMS rotators. As a consequence, their cumulative XUV radiation is higher than for slower rotators, especially during the first Gyr of evolution (cf. Johnstone et al. 2021a, Fig. 18). This may lead to the complete evaporation of the primordial atmospheres of close-in Neptune-sized planets (Tu et al. 2015).

There is an indication that the angular momentum loss rate decreases below the value predicted by the Skumanich law in solar-like stars older than the Sun (van Saders et al. 2016; Hall et al. 2021; David et al. 2022). This should keep their XUV at higher values than predicted based on the Skumanich law. Other effects, such as the stalled rotational evolution observed in stars less massive than the Sun between 0.6 and 1.5-2 Gyr, (e.g., Curtis et al. 2020), may also affect the XUV cumulative flux received by their planets, although not significantly compared to the XUV flux received within the first 0.3-0.5 Gyr of their evolution. Second-order effects can be related to the stellar metallicity affecting the rotational evolution (Amard et al. 2020) and the coronal emission of the stars (Poppenhaeger 2022).

Although the XUV flux decreases at least by an order of magnitude during the first few 100 Myr for most M-F stars (e.g. Johnstone et al. 2021a), the $F_{\mathrm{XUV}}$ received by a planet can remain high enough to rapidly erode an atmosphere over longer timescales. As the upper panel of Fig. 13 illustrates for various stellar masses, the $F_{\mathrm{XUV}}$ at the middle of the HZ of lower-mass stars takes significantly longer to drop below a certain threshold than the $F_{\mathrm{XUV}}$ of higher-mass stars. This figure shows the XUV surface flux evolution of so-called moderate rotators (i.e., stars belonging to the 50th percentile of the initial $P_{rot}$-distribution of their stellar mass) calculated with the stellar evolution model Mors Johnstone et al. (2021a) and the stellar isochrones from Spada et al. (2013), respectively. The dashed and dotted grey lines in Fig. 13 show the thermal stability thresholds for CO_2_-dominated (99% CO_2_, 1% N_2_) and N_2_-dominated (90% N_2_, 10% CO_2_) atmospheres hosted by a 1 M_⊕_-planet (Van Looveren et al. 2024). As long as an evolving star lies above one of these thresholds, the respective atmosphere is not thermally stable in its HZ due to XUV heating of the upper atmosphere (Sect. 2.1). Only stars below these thresholds can hence host temperate planets with these certain types of secondary atmospheres (see Sect. 6). The HZ planet Trappist-1 e, for instance, lies above both thresholds (values from Wheatley et al. 2017) although its host star has a mass of ∼0.09 $M_{\odot}$ and is ∼8 Gyr old. It indicates that Trappist-1 may never reach an $F_{\mathrm{XUV}}$ in its HZ that allows for the retention of a secondary atmosphere (as CO_2_-atmosphere is expected to be the most thermally stable among them).

In contrast, another HZ planet included in Fig. 13 – the Earth – is clearly below both thresholds. The Sun is a 4.56 Gyr old quiescent G star that appears anomalously weakly active compared to other solar-type stars (Reinhold et al. 2020). Its XUV is below that of the average G-type star of similar age (Johnstone et al. 2021a), as well as super-flaring rates. Whereas the average Sun-like star produces super-flares with energies $<10^{34}$ erg roughly once per century (Vasilyev et al. 2024) , no such energetic super-flares have been observed at the Sun – with the largest flare observed to date being the Carrington event with estimated energies of $~\sim 5\times 10^{32}$ erg (e.g., Hayakawa et al. 2023). Assuming the Sun can produce super-flares, the occurrence rate is estimated to be $\sim 10^{-3}$ per year (Shibata et al. 2013), about an order of magnitude lower than the average for solar type stars. If the Sun is indeed anomalously weakly active, this has far-reaching implications for habitability as secondary atmospheres are highly susceptible to atmospheric erosion around active stars (Scherf et al. 2024).

Finally, the bottom panel of Fig. 13 shows the same data as the top panel but normalized to the XUV surface flux evolution of a moderately rotating solar-like star. This plot is therefore valid for any orbital separation, for which the respective stars induce the same total incident stellar surface flux, $S_{\mathrm{eff}}$. Figure 13 clearly shows that stellar mass and age are crucial parameters for the retention and loss of planetary atmospheres. See also Scherf et al. (2024) for an in-depth discussion on this issue.

### Stellar Magnetized Winds

Stellar winds interact with planetary atmospheres, especially those not shielded by a magnetosphere (see Sect. 2.2). Unfortunately, we only have detailed information for the solar system and mostly for the present-day Sun close to the plane of the ecliptic. However, a stellar wind is a complex 3D MHD flow that depends on the level of activity of the star and is perturbed by transients produced by flares and CMEs.

Stellar wind mass loss rates (a proxy of the wind’s speed and density) have been measured only for about a dozen stars, based on different indirect techniques. The dependence of the wind mass loss flux on the coronal X-ray flux has been investigated by Vidotto (2021, 2023) providing information to study the wind impact on the evolution of planetary atmospheres. Even less understood is the impact of CMEs associated with major stellar flares on exoplanetary atmospheres because of the difficulties in observing them (cf. Sect. 1 of Xu et al. 2024). An extrapolation based on solar CMEs to more active stars may not be appropriate because the mechanism producing and accelerating CMEs can be less efficient in more active stars than in the Sun (Alvarado-Gómez et al. 2018). However, some works attempt to estimate the CME frequencies at stars of different types through measurable parameters, such as temperatures and stellar spot occurrence (e.g. Herbst et al. 2021).

Though the impact of CMEs is well studied for solar system planets (e.g. Luhmann et al. 2007; Edberg et al. 2010, 2011), no direct observations are available for exoplanets. Theoretical modelling suggests that CMEs can lead to some enhancement of the atmospheric escape (e.g. Lammer et al. 2007; Lynch et al. 2019; Alvarado-Gómez et al. 2022; Hazra et al. 2025) but also to an enhancement of prebiotic atmospheric chemistry (e.g. Kay et al. 2019; Airapetian et al. 2016).

### Tides in Planets and Stars

Tides have been reviewed by Ogilvie (2014), while a number of other works have been published in the context of star-planet interactions, e.g., Van Laerhoven et al. (2014), Blackledge et al. (2020), Lanza (2022). Tidal dissipation inside a planet produces heating in its interior, tends to decrease the eccentricity of its orbit, and synchronizes its rotation with the orbital motion, except when the planet has a permanent quadrupolar deformation as in the case of Mercury, in which case its rotation can be captured in a spin-orbit resonance (e.g., Ch. 5 of Murray and Dermott 1999). On the other hand, thermal atmospheric tides are considered to account for the retrograde and very slow rotation of Venus (Dobrovolskis and Ingersoll 1980; Dobrovolskis 1980; Correia and Laskar 2010). By secularly modifying the star-planet separation, tides in the star influence the amount of flux received by the planet. The rotation of the star can also be affected, if the planet is sufficiently massive, thus affecting the XUV flux received by the planet (Attia et al. 2021).

The dissipation of the tides inside a planet raised by the host star is usually parametrized by the modified tidal quality factor $Q^{\prime}_{p}$ (e.g., Zahn 2008). In most applications, given our uncertainty on tidal dissipation, $Q^{\prime}_{p}$ is assumed to be constant, thus greatly approximating the modelling of tidal effects. The power $P_{\mathrm{tide}}$ dissipated inside a (pseudo)-synchronized planet on a slightly eccentric orbit ($e < 0.2$) can be expressed as (cf. Eq. (4) of Miller et al. 2009)

10 $$ P_{\mathrm{tide}} = \frac{63}{4} \left [ (GM_{s})^{3/2} \left ( \frac{M_{s} R_{\mathrm{pl}}^{5} e^{2}}{Q^{\prime}_{p}} \right ) \right ] a^{-15/2}, $$

where $G$ is the gravitational constant, $M_{s}$ the mass of the star, $R_{\mathrm{pl}}$ the radius of the planet, $e$ the eccentricity of the orbit, and $a$ the orbit’s semimajor axis. For a highly eccentric orbit, the $Q^{\prime}_{p}$ parametrization breaks down and a different approach must be used that allows one to include the effects of non-synchronous rotation (Leconte et al. 2010). For terrestrial planets, typical values of $Q^{\prime}_{p}$ range from a few tens to a few thousands, while for planets with a sizeable gaseous envelope or giant planets, values from a few $10^{4}$ up to $10^{5}-10^{6}$ are usually adopted (cf. Lainey 2016, in the case of the solar system). However, given our limited understanding of tidal dissipation inside planets and stars (Ogilvie and Lin 2007; Ogilvie 2014), predicting an accurate value of $Q^{\prime}_{p}$ for a specific planet is highly challenging. The physical mechanism of tidal heating is discussed in more detail in Lourenço et al. (2026).

Tidal heating in a terrestrial planet can produce relevant volcanic activity (for details see e.g. Lourenço et al. 2026, this collection), thus influencing the composition of its secondary atmosphere (see Sect. 6). It can also lead to the enhancement of thermal and non-thermal escape processes (Sect. 2.3). An average non-zero orbital eccentricity can be maintained by the perturbations of other planets in a compact system, especially in the case of a resonant chain such as in Trappist-1 (e.g., Barr et al. 2018).

### Star-Planet Magnetic Interactions

The interaction between the stellar magnetic field and a close-in planet produces phenomena that can indirectly probe the planet’s magnetic field (e.g., Shkolnik et al. 2008; Cohen et al. 2011; Saur et al. 2013; Cauley et al. 2019). In addition to tides, the angular momentum loss of the star can be affected by the field of a closely orbiting giant planet (Lanza 2010; Tejada Arevalo et al. 2021), thus modifying the evolution of its magnetic activity. In some cases, stellar flare activity could be affected as well (Loyd et al. 2023; Ilin et al. 2024, 2025). The latter can, in turn, increase the planet’s atmosphere evaporation rate (Ilin et al. 2025).

If the planet has no intrinsic magnetic field, its motion through the magnetic field of the star (closed corona or open stellar wind structures) produces an electric induction in its interior and ionosphere, provided that the magnetic flux is time-dependent. The amount of heating in the different layers – ionosphere, salty ocean, or interior – depends critically on their electric conductivity (Kislyakova et al. 2017, 2018; Kislyakova and Noack 2020). This is relevant only for very close-in planets orbiting strongly magnetized stars, such as young late-type stars or late M-type stars such as Proxima Centauri or Trappist-1. The magnetic fields associated with coronal mass ejections may also produce induction heating (Grayver et al. 2022).

The strong and varying magnetic field along the close-in orbit of a planet around an M star additionally induces an electric current in its ionosphere whose dissipation results in strong Joule Heating (JH) of the upper atmosphere. For Trappist-1 e, the JH energy flux is likely larger than the incident XUV surface flux of ∼0.3 W m^−2^ (∼300 erg/s/cm^2^, e.g., Wheatley et al. 2017, see also Fig. 13) and may even approach up to a few percent of the entire stellar energy flux received by the planet in the case that its ionosphere thickness reaches ∼10,000 km (Cohen et al. 2024). Such extended ionospheres can indeed be expected for secondary atmospheres on close-in planets like Trappist-1e (e.g., Cohen et al. 2024; Nakayama et al. 2022), thereby implying intense heating of the upper atmosphere and strongly enhanced atmospheric escape.

When the planet has an intrinsic magnetic field, magnetic reconnection, and particle acceleration occur at the boundary of its magnetosphere (e.g. Lanza 2009, 2012; Buzasi 2013) and energy can be conveyed towards the star and the planetary atmosphere. The maximum power available in the case of a hot Jupiter interacting with a Sun-like star can reach $10^{15}-10^{18}\text{ W}$ (or $10^{22}-10^{25}$ erg/s; Lanza 2009, 2012). A remarkably larger amount of power is predicted when a loop interconnecting the planetary field with the stellar field is formed, reaching a maximum of $10^{20}-10^{21}\text{ W}$ (or $10^{27}-10^{28}$ erg/s; Lanza 2013).

## Role of Planetary Mass (Escape Velocity)

From Sect. 2, one can derive that thermal escape processes, if effective at the planet, are generally expected to dominate planetary atmospheric outflow. Hence, the escape from closer-in planets is expected to be stronger. However, besides the level of incoming stellar radiation, other planetary parameters are at play. To first order the most important one is planetary mass or, for planets subject to inflation (e.g., due to a combination of high internal thermal energy and low mass), the interplay between the mass and the radius of the planet, as discussed in Sect. 2.3.

Even planets located far from their host star, hence less subject to intense thermal escape processes, can lose the entirety of their atmospheres if their mass is too small. In the Solar System, a good example of such a planet is Mars, which is significantly farther from the Sun, but about ten times less massive than Earth.

**Fig. 14:**
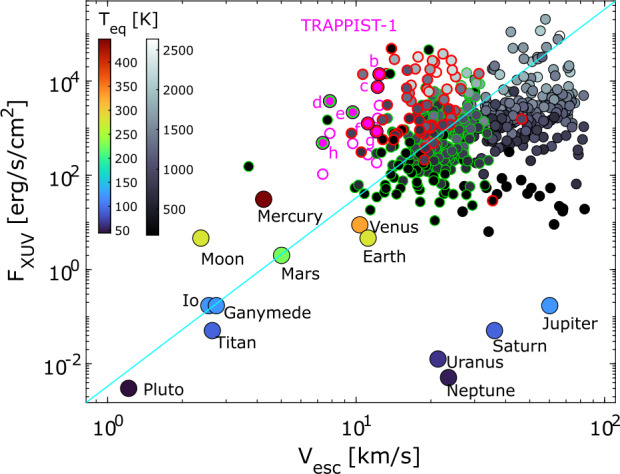
The Cosmic Shoreline following Zahnle and Catling (2017). The XUV flux for a given planetary orbit was estimated by employing Mors stellar evolution code (for planets orbiting stars with masses 0.1-1.2 $M_{\odot }$ Johnstone et al. 2021a) or the analytical approximations by Wright et al. (2011) and Sanz-Forcada et al. (2011) (for lighter and heavier stars). The colour of the dots reflects planetary equilibrium temperature (assuming zero albedo), as indicated by the colourbars in the plot; we additionally highlight the planets in Trappist-1 system with filled magenta markers. For comparison, empty magenta markers show the XUV flux estimates of Trappist-1 system from Wheatley et al. (2017). Green and red outlines denote the exoplanets lighter than 40 $M_{ \oplus}$ with H-He atmosphere fraction larger and smaller than $10^{-5}$ according to the model by Johnstone et al. (2015), respectively

To illustrate the point, in Fig. 14 we present the distribution of the known exoplanets in the escape velocity ($v_{\mathrm{esc}}$) – XUV flux ($F_{\mathrm{XUV}}$) plane following Zahnle and Catling (2017). Here, we use the data from the NASA Exoplanet Archive^3^ and the NASA Planetary Fact Sheet^4^ for the Solar System bodies. We only consider planets with masses smaller than $2\,M_{jup}$, mass uncertainties smaller than 45%, and radius uncertainties smaller than 15%. For exoplanets, we used the following approach. To estimate the $F_{\mathrm{XUV}}$ at planetary orbits consistently for all planets, we employed the Mors stellar evolutionary code (Johnstone et al. 2021a; Spada et al. 2013) to calculate the fluxes according to the mass and age of their host stars, assuming that all stars have evolved as moderate rotators. For the planets orbiting stars outside the applicability limit of the Mors code (which is, $0.1-1.2$ $M_{\odot}$), we used the empirical approximations by Wright et al. (2011) and Sanz-Forcada et al. (2011) as described in Kubyshkina et al. (2019), which are expected to provide average values. If the age of the star was not available in the Exoplanet Archive, we assigned it to the average value of 5 Gyr, except for the Trappist-1 system where the age was set to 7.6 Gyr (Burgasser and Mamajek 2017). The cyan line in Fig. 14 ($F_{\mathrm{XUV}}\,\sim \,v_{\mathrm{esc}}^{4}$) is expected to separate the planets capable of keeping some type of an atmosphere (below the line, where $F_{\mathrm{XUV}}$ is low and $v_{\mathrm{esc}}$ is high) from the bare cores (above the line); following Zahnle and Catling (2017). We set the proportionality coefficient more or less arbitrarily (as in Zahnle and Catling (2017)), assuming that Mars was on the borderline of keeping its atmosphere.

The first thing one notices when analysing Fig. 14 is that the Solar System planets and the exoplanets known to date (at least, those with well-constrained masses and radii) occupy different regions both in the $F_{\mathrm{XUV}}$-$v_{\mathrm{esc}}$ diagram and in terms of equilibrium temperature. Furthermore, the exoplanets’ population does not seem to align with the $F_{\mathrm{XUV}}\,\sim \,v_{\mathrm{esc}}^{4}$ as lower-mass planets of the Solar System. Instead, they follow a shallower slope, in particular at high planetary masses.A few contributors seem to be at play in smoothing the dependence: generally higher equilibrium temperatures (e.g. a larger contribution from interior heat, and infrared heating from the planetary core), tidal forces of the host star, and radius inflation due to star-planet interaction.

If one assumes that, for exoplanets, division between planets with and without atmospheres occurs along a line (power law) in $F_{\mathrm{XUV}}$-$v_{\mathrm{esc}}$ diagram, the shallower dependence implies that, for a given $v_{\mathrm{esc}}$, the transition occurs at lower XUV level that predicted by $F_{\mathrm{XUV}}\,\sim \,v_{\mathrm{esc}}^{4}$ dependence shown in Fig. 14. However, this statement is currently impossible to verify, as we do not know if there is an atmosphere at any given exoplanet, unless it is a substantial H-He dominated atmosphere. Even in the latter case, defining the actual atmospheric mass fraction from observable parameters is tricky, as all the available internal structure models are strongly degenerated, in particular for small atmospheric mass fractions (e.g. Egger et al. 2025, and references therein). To illustrate this point, in Fig. 14, we include predictions on the presence/absence of H-He atmospheres for planets lighter than 40 $M_{ \oplus}$ based on simple structure model from Johnstone et al. (2015) (assuming pure H-He atmospheres and fixed core parameters). Red and green markers’ outlines show planets without and with H-He atmospheres (where the boundary atmospheric mass fraction value is taken at $10^{-5}$). Though the transition between the two groups of planets occurs near $F_{\mathrm{XUV}}\,\sim \,v_{\mathrm{esc}}^{4}$ line, they strongly overlap, not allowing to retrieve a clear boundary.

Besides controlling the critical level of irradiation leading to atmospheric evaporation, planetary mass can affect specific escape channels. This can affect the composition of material leaving the planet. For non-thermally escaping atmospheres, the larger mass means a higher fraction of ion escape compared to neutrals (see Sect. 2.2). For thermally escaping atmospheres, the fraction of ions in the escaping material at high altitudes is also expected to increase: at a given $T_{\mathrm{eq}}$, ionisation fronts are more narrow for more massive planets. Thus, for heavy planets, the atmosphere is fully ionised at the exobase, while for low-mass planets this is not necessarily the case (in particular, if the planet is hot; e.g. Kubyshkina et al. 2018b; Guo 2024).

We highlight the fact that atmospheric escape could exhibit a complex dependence on the planet’s size, through a simplified analysis that parallels Chin et al. (2024). Suppose that we have a planet of radius $R_{\mathrm{pl}}$. As the size (or radius) of the planet is expanded, its cross-sectional area would roughly increase as $R_{\mathrm{pl}}^{2}$, if we assume a purely geometric scaling. In this case, a higher fraction of the electromagnetic radiation (extreme UV and X-rays) and stellar wind emitted by the host star would be intercepted by the planet’s atmosphere. Hence, this would translate to an increase in the atmospheric escape rate. On the other hand, if we continue to increase the planet’s size further (and assume that the atmosphere does not contribute to $R_{\mathrm{pl}}$), we run into the issue that the escape velocity of the planet also increases, which obeys

11 $$ v_{\mathrm{esc}} = \sqrt{\frac{G M}{R_{\mathrm{pl}}}} \propto R_{\mathrm{pl}}^{1.64}, $$

where we have used the mass-radius relationship for rocky planets $M_{\mathrm{pl}}\,\propto \,R_{\mathrm{pl}}^{3.7}$ (Zeng et al. 2016). Hence, as the planet’s size increases, the corresponding rise in the escape velocity implies that fewer particles would possess the requisite energy to escape the planet’s gravitational well.

To summarize the previous discussion, we expect the atmospheric escape rate to exhibit a potentially non-monotonic dependence on the planet size, as the planet transitions from a source-limited regime (influenced by the planet’s cross-sectional area) to the energy-limited regime (modulated by the planet’s escape velocity). As we have not specified what the driver of atmospheric escape is, it may be anticipated to hold true for different types of atmospheric escape, albeit to varying degrees and with different scalings.

In the case of ion escape, the trend delineated in the preceding paragraph appears to be consistent with empirical data from Mars and Venus, which may be source- and energy-limited, respectively (Persson et al. 2020; Ramstad and Barabash 2021). Moreover, the above prediction has been corroborated through state-of-the-art simulations of putative exoplanets performed by Chin et al. (2024), who found that ion escape peaked at the radius of $\sim 0.7\,R_{\oplus}$ for selected stellar wind parameters.

Turning our attention to late-type hydrodynamic escape powered by XUV radiation (considering an energy-limited approximation Erkaev et al. 2007), a similar trade-off may be manifested (e.g. Lingam and Loeb 2021). If we take the size of the exobase to be loosely comparable to the planetary radius – which is not strictly valid even for terrestrial planets (e.g. Lammer 2013) – the escape rate in the energy-limited regime obeys the simple scaling $R_{\mathrm{pl}}^{3}/M_{\mathrm{pl}} \propto R_{\mathrm{pl}}^{-0.7}$ (Lingam and Loeb 2019), where we have used the mass-radius relationship from earlier. In contrast, if we consider the photon-limited regime (analogous to the source-limited regime), the atmospheric escape scales as $R_{\mathrm{pl}}^{2}$ (Owen and Alvarez 2016). Therefore, we can explicitly see how an increase in the radius would permit an increase in the atmospheric escape rate in the photon-limited regime. This is followed by a decrease in the energy-limited regime, mirroring our earlier statements. This trend is compatible with the results of numerical simulations (e.g. Chen and Rogers 2016).

## Role of Atmospheric Composition in Escape

### Known Atmospheric Types

The types of (collision-dominated) atmospheres of terrestrial planets can broadly be divided into (i) primordial atmospheres, (ii) steam atmospheres, (iii) secondary atmospheres, and (iv) silicate atmospheres. In addition, (v) so-called airless bodies host a thin, collision-less atmosphere commonly called an exosphere (Lammer et al. 2022). Primordial atmospheres are the only ones that are not degassed from the planet’s interior but are instead accreted during the planetary formation stage from the nebula surrounding the newly formed star (e.g. Hayashi et al. 1979). Since the stellar nebula is compositionally similar to its host star, pristine primordial atmospheres consist mostly of hydrogen with a smaller amount of helium, and metals (elements heavier than He) being present only in trace amounts. The more massive a planet grows within the protostellar nebula, the more hydrogen and helium it will accrete. The size of the atmosphere is further constrained by the amount of gas available around a forming planet, hence, its position within the disk. For a planet in the HZ of solar-like stars, a mass around 1 $M_{\oplus}$ seems to be the limit below which the entire primordial atmosphere can be lost subsequently (Erkaev et al. 2023; Lammer et al. 2025). For lower-mass host stars and shorter orbits, this mass limit will be higher and vice versa. Furthermore, if the irradiation from the host star and the planet’s mass are at a certain sweet spot, a hydrogen-dominated primordial atmosphere can become helium-dominated through fractionation: most hydrogen is lost into space, while part of the heavier He remains at the planet (e.g., Hu et al. 2015).

Steam atmospheres, on the other hand, which were initially assumed to be purely dominated by H_2_O and minor amounts of CO_2_, form either through (i) degassing from a planet’s solidifying magma ocean (e.g., Elkins-Tanton 2011), (ii) the evaporation of an ocean, or (iii) through migration of an ice- and/or water-rich planet toward its star (e.g., Burn et al. 2024). For case (i), the steam atmosphere can subsequently condense and form oceans if the planet is within the HZ. Closer to the star, the planet can evolve differently. In case of Venus, for instance, it is currently disputed whether the degassed steam atmosphere was initially able to condense to form early oceans (e.g., Hamano et al. 2013; Lebrun et al. 2013; Salvador et al. 2017; Way and Del Genio 2020; Salvador et al. 2023) or the temperature at its orbit was always too hot for condensation (e.g., Hamano et al. 2013; Lebrun et al. 2013; Salvador et al. 2017; Krissansen-Totton et al. 2021; Turbet et al. 2021; Salvador et al. 2023). If it does not condense, a steam atmosphere can also escape into space. In this case, oxygen provided by the dissociation of H_2_O can accumulate at the planet, as it escapes less efficiently than H due to its weight. In addition, steam atmospheres generally escape less efficiently than primordial atmospheres, not only because of their higher molecular weight but also because H_2_O acts as an efficient coolant (e.g. Yoshida et al. 2022). However, recent studies (e.g., Bower et al. 2022; Maurice et al. 2024) have put into question the entire foundation of what the constituents are for such atmospheres. They suggest that steam atmospheres are not viable for some magma ocean redox states and C/H ratios (Earth’s C/H ∼1). In the case that Earth did not have a steam atmosphere over its magma ocean, there are few ideas on exactly how the Earth obtained its initial Hadean era liquid water ocean (e.g., Miyazaki and Korenaga 2022).

Secondary atmospheres may be initially degassed by volcanic activity after a primordial atmosphere was lost and the subsequently degassed hypothesized steam atmosphere either condensed or was also lost into space. These atmospheres can have very different compositions, structures, and densities and are inclined to change substantially over a planet’s history due to various abiotic, and potentially biotic, processes. The present-day Solar System illustrates part of the diversity secondary atmospheres can exhibit. For example, atmospheres have likely ranged from N_2_–CO_2_-dominated (Archean Earth), N_2_–O_2_-dominated (modern Earth) and N_2_–CH_4_-dominated (Titan) toward thick (Venus) and thin (Mars) CO_2_-dominated atmospheres. However, all these bodies likely had a different atmospheric compositions through time. Additional types of secondary atmospheres can be envisioned to exist in the Universe such as CO- (Zahnle et al. 2008) and O_2_-dominated ones (Wordsworth and Pierrehumbert 2014; Luger and Barnes 2015). Crucially, their compositions lead to very different upper atmosphere structures, as different molecules have different molecular weights and interact differently with the incoming XUV flux of the host star. CO_2_, for instance, is a strong infrared coolant whereas N_2_ is not. Together with their different molecular weights, this is an important reason why the upper atmospheres of Earth and Mars/Venus look so different (see, e.g., Way et al. 2023).

Planetary bodies that are close enough to their host star, such as CoRoT-7 b (Léger et al. 2011), may experience surface temperatures high enough to melt and vaporize rock, which then either escapes into space or forms a silicate atmosphere consisting of species such as Na, K, and SiO (e.g., Ito et al. 2015). Although silicate atmospheres have a relatively high molecular weight, only massive planets can prevent their escape, as they are genuinely exposed to very high XUV fluxes. Although recent observations of the Super Earth 55 Cancri e (Hu et al. 2024) demonstrate that it may have an atmosphere dominated by volatiles, not silicates. If a rocky body such as Mercury is far enough from the star for its mantle to solidify but still close and small enough to lose any (collision-dominated) atmosphere, it will still keep an exosphere dominated by, e.g., O, K, and Na. This exosphere is balanced by processes that release particles from its surface (e.g., sputtering, photodesorption, and micrometeorite impacts; see Wurz et al. 2022) and by their escape into space.^5^ For small icy bodies, the exosphere can be dominated by interaction with their UV and particle environments (e.g., Europa, Callisto, and Ganymede) and/or the equilibrium vapour pressure of its constituents at the respective surface temperatures. The latter can be observed at Pluto and Triton whose exospheres are dominated by N_2_ with minor amounts of CH_4_ and CO (e.g., Scherf et al. 2020).

### Primordial (Nebular) Atmosphere

Formation models predict that the majority of planets, including low-mass ones, accrete hydrogen-dominated atmospheres with compositions resembling that of their host stars, while embedded in their protoplanetary discs (e.g. Jin and Mordasini 2018; Morbidelli 2020; Venturini et al. 2020; Burn et al. 2021). For terrestrial-like planets, such atmospheres contribute less than $\sim 1\%$ to the total planetary mass (e.g. Mordasini 2020), and their lifetime after the protoplanetary disk dispersal is short ($\lesssim 1-100$ Myr; e.g. Lammer et al. 2008a; Owen and Wu 2016). However short the lifetimes of primordial atmospheres are, the extreme outflow of hydrogen during this early phase of planetary evolution can have long-term consequences. First, if the atmospheric escape rate exceeds a certain limit, hydrogen outflow can drag along some heavier elements (the critical mass, i.e., the maximum mass of the species that can escape alongside hydrogen, is defined by the outflow parameters). This leads to the fractionation of atmospheric species (e.g. Öpik 1963; Zahnle and Kasting 1986; Hunten et al. 1987; Pepin 1991; Odert et al. 2018; Lammer et al. 2020b). Second, the fractionation of radioactive elements can adjust the planet’s thermal budget (Erkaev et al. 2023). Furthermore, as long as a hydrogen-dominated atmosphere is present, it can interact with the surface and facilitate water production (e.g. Ikoma and Genda 2006; Ikoma et al. 2018; Lammer et al. 2021; Salvador et al. 2023; Rogers et al. 2024) and, hence, alter the interior composition (e.g. Rogers et al. 2024). Besides the direct impact on atmospheric composition, the lifetime of the primordial atmosphere sets the timescale of the formation of the steam and secondary atmospheres dominated by elements heavier than hydrogen. Hence, it can affect whether, when, and under which external conditions the secondary atmosphere is formed.

The early phase (first ∼100 Myr for Sun-like stars and up to ∼1 Gyr for late M-dwarfs) of atmospheric evaporation is expected to occur in the hydrodynamic regime, while the main driving mechanism and duration of this phase for a specific planet can vary depending on primordial planetary parameters. As discussed in Sects. 2.1.3 and 5, the type and strength of the outflow depends strongly on the planet’s mass and irradiation level set by the planetary orbit and properties of the host star. For low-mass (lighter than $\sim 10\,-\,30$ ${M_{\oplus}}$) planets on short orbits, early escape is likely to occur on account of stellar (bolometric) heating, i.e., in the boil-off/core-powered regime. It will later transition into an XUV-driven regime, while for more massive and distant planets, the escape can be XUV-driven from the start (e.g. Guo 2024). This type of escape is particularly sensitive to stellar input: while the bolometric luminosity of a star (especially for Sun-like stars) does not change dramatically over the main sequence, the XUV luminosity at young ages (see Sect. 4.2) can be orders of magnitude above that of Gyr-old stars.

Whether the hydrogen-dominated atmosphere survives this phase of extreme mass loss depends on the planet’s mass and orbit: increase in the planetary mass (increasing escape velocity) allows a low-mass planet to keep its atmosphere with decreasing orbital separation (e.g. Lopez and Fortney 2014; Chen and Rogers 2016; Kubyshkina and Vidotto 2021). The renowned observational confirmation of this dependency is the negative slope of the radius valley (which is a lack of planets with intermediate radii $\sim 1.5$ $R_{\oplus }$ in sub-Neptunes’ population within ∼100 days orbit Fulton et al. 2017) in exoplanets’ distribution in the radius-period plane (e.g. Owen and Wu 2017; Martinez et al. 2019b). The position of the centre of the valley (minimum in $R_{\mathrm{pl}}$ distribution at a given orbital period $P_{\mathrm{orb}}$) changes with orbit as $R_{\mathrm{valley}} \sim (P_{\mathrm{orb}}/10$ days$)^{0.1}$. The origin of the valley is commonly attributed to the presence of H-dominated atmospheres at some planets. At high equilibrium temperatures, structure models (e.g. Stökl et al. 2015) predict that even tiny ($\lesssim 0.1\%$ of $M_{\mathrm{pl}}$) H-dominated atmospheres can lead to the significant inflation of planetary radii. In this context, both core-powered and XUV-driven escape mechanisms can reproduce the valley (e.g. Affolter et al. 2023). With increasing orbital separation, both the radius inflation and the maximum bare core (MBC, which is roughly equivalent to the minimum planetary mass needed to keep the primordial atmosphere at the given orbit) mass decrease, and the radius valley closes. However, the MBC mass remains an important parameter to assess a planet’s evolution. Thus, for the Earth, it was estimated to be slightly below the present-day mass, indicating that the planet reached its final mass after protoplanetary gas disk dispersal (otherwise, it would keep its primordial atmosphere; on the other hand, the mass had to be high enough to accrete some of it, to explain the noble gases inventory; Lammer et al. 2020a; Erkaev et al. 2023; Lammer et al. 2025).

Besides the dependence upon basic planetary parameters, the atmospheric evaporation timescale of H-dominated atmospheres from low-mass planets depends on their primordial atmospheric mass fraction. Models predict that the lifetime of atmospheres maximizes at intermediate initial atmospheric mass fractions, that is typically 1-3% of the planetary mass for the sub-Neptune-mass planets subject to significant atmospheric losses. The specific value increases with increasing planetary mass and orbital separation, and (for a given $T_{\mathrm{eq}}$) with decreasing mass of the host star (e.g. Chen and Rogers 2016; Kubyshkina and Vidotto 2021). The effect is most pronounced for planets with masses near the MBC one for their respective orbit and host star.

The discussion above concerns mainly low-mass and/or close-in planets. In the case of cool giants, the loss of primordial atmospheres is dominated by Jeans-like or non-thermal escape processes, such as charge exchange (e.g. Parkinson 2002; Mauk et al. 2020).

### Venus and Mars-Type CO_2_-Dominated Atmospheres: Implications for Exoplanets

Although Venus is comparable in size and mass to the Earth, the planet’s surface shows no indications of modern Earth-style subductive plate tectonics. The same applies to the Martian surface. Today Venus and Mars are surrounded by CO_2_-dominated atmospheres ($\approx 92\text{ bar}$ and $\approx 6.5$ mbar) that consist of 96.5% CO_2_ with 3.5% N_2_ (Oyama et al. 1980) and $\approx 95.97$% CO_2_ with $\approx 1.89$% N_2_ (Mahaffy et al. 2013), respectively. Weller et al. (2023) modelled secondary atmospheres and compared them with modern Venus’ atmosphere. They found that volcanic outgassing in an early phase of plate-tectonic-like activity during the first billion years, followed by a stagnant-lid-phase that lasts until today, can best explain Venus’ dense CO_2_ atmosphere and especially its present-day high N_2_ content. As addressed in detail in Scherf and Lammer (2021), it is not likely that Mars could build up a dense secondary CO_2_ atmosphere during its first 400 Myr. Afterward, it may have had a denser atmosphere from sporadic asteroid impacts and large volcanic events. These could have resulted in sporadic warmer climate periods $\approx 3-4$ Gyr ago Scherf and Lammer (2021), Lichtenegger et al. (2006), Schmidt et al. (2022), Kamada et al. (2020), Wordsworth (2016), Turbet and Forget (2019), Forget et al. (2013), Guzewich et al. (2021) with a denser CO_2_ or CO-dominated atmosphere. This atmosphere must have been lost later by CO_2_ sequestration into the soil and non-thermal atmospheric escape processes (Lammer et al. 2018; Lichtenegger et al. 2022; Wordsworth 2016).

Because Venus and Mars have a similar atmospheric composition up to the exobase level, one can estimate their average exobase temperatures by a scaling method that can also be applied to exoplanets as long as the atmosphere remains under hydrostatic conditions. From the effective heat production below the exobase level, which is balanced by the divergence of the conductive heat flux of the XUV radiation, one gets the following simplified expression for the exobase temperature $T_{\mathrm{exo}}^{j}$ (Bauer 1971; Gross 1972)

12 $$\begin{aligned} T_{\mathrm{exo}}^{j}\approx \frac{\eta _{j}\; F_{\mathrm{XUV}}\; k_{B} \; \sigma _{{c}_{j}}}{\alpha \; K_{0_{j}} \; m_{j} \; g \; \sigma _{{a}_{j}}}+T_{0}, \end{aligned}$$

where $\sigma _{{c}_{j}}$ and $\sigma _{{a}_{j}}$ are the collision and absorption cross-sections of species $j$, $K_{0_{j}}$ is the thermal conductivity of species $j$ at the reference temperature, $\alpha $ is a factor related to the planetary rotation ($\approx 0.25$ for fast-rotating planets like Earth and $\approx 0.5$ for tidally locked planets). $T_{0}$ is the temperature at the base of the thermosphere, which can also be approximated with the homopause temperature. If the homopause temperature is unknown one can adopt $T_{0}\simeq T_{\mathrm{eq}}$, which has a negligible effect if $T_{\mathrm{exo}}^{j} \gg T_{0}$.

For Earth’s N_2_/O_2_-dominated upper atmosphere with its very low atmospheric CO_2_ abundance, the present Sun’s XUV flux results in a $T_{\mathrm{exo}}\approx \,1000--1200\text{ K}$ (Jacchia et al. 1977; Crowley 1991). Although Venus is closer to the Sun, due to its 96% CO_2_ atmosphere the average dayside $T_{\mathrm{exo}}$ is only $\approx 285\text{ K}$ (von Zahn et al. 1980; Hedin et al. 1983; Müller-Wodarg et al. 2006). A main reason for cooler Venus’ thermosphere-exosphere environment compared to Earth is a very efficient cooling of the upper atmosphere in Infra-Red (IR) emission of the 15 μm CO_2_ band (Gordiets et al. 1982; Gordiets and Kulikov 1985; Müller-Wodarg et al. 2006).

If two planets have similar atmospheres, their planetary parameters are known and the atmospheres of both planets can be characterized by high Jeans escape parameters (with $\lambda \gg 6$), one can use Eq. (12) in the case that $T_{\mathrm{exo}}$ is known for one planet to estimate the unknown exobase temperature of the second. Since Venus and Mars have a similar atmospheric composition $j =\mathrm{CO}_{2}$ in their thermosphere, one obtains the following scaling relation (Bauer 1971),

13 $$\begin{aligned} \frac{\left (T_{\mathrm{exo}}^{\mathrm{CO}_{2}} - T_{0}\right )_{\mathrm{Venus}}}{\left (T_{\mathrm{exo}}^{\mathrm{CO}_{2}}- T_{0}\right )_{\mathrm{Mars}}} = \frac{F_{\mathrm{XUV_{\mathrm{Venus}}}} g_{\mathrm{exo}}^{\mathrm{Mars}}}{F_{\mathrm{XUV}_{\mathrm{Mars}}} g_{\mathrm{exo}}^{\mathrm{Venus}}}. \end{aligned}$$

One can see from Eq. (13) that $T_{\mathrm{exo}}^{\mathrm{CO}_{2}}$ depends on $F_{\mathrm{XUV}}$, which decreases with distance from the star, and on the planet’s gravitational acceleration at the exobase level $g_{\mathrm{exo}}$, which is related to the exobase position $r_{\mathrm{exo}}$ and the planetary mass. Using the average exobase temperature of Venus and the corresponding values for $F_{\mathrm{XUV}}$ at the orbits of Venus and Mars, the corresponding $g$ values for both planets at the exobase level of 200 km and $T_{0}$ of 220 K and 160 K for Venus and Mars, one obtains an average $T_{\mathrm{exo}}^{\mathrm{CO}_{2}}$ for Mars of ≈200 K, which is in agreement with the Martian exobase temperature inferred by MAVEN spacecraft of $\approx 239.2\pm 27.6\text{ K}$ near perihelion and $\approx 162.4 \pm 19.9\text{ K}$ near aphelion (Qin 2020). One can also make the same estimate the other way around by getting the exobase temperature at Venus from $T_{\mathrm{exo}}^{\mathrm{CO}_{2}}$ of Mars. Similarly, the same equation can be utilized to estimate the exobase temperature of exoplanets as long as these are hydrostatic and the exobase level is known, as $g_{\mathrm{exo}}$ can diverge significantly from the gravitational acceleration on the surface of a planet for extended atmospheres. If we, we for instance, take the simulation for a planet with $1.0\,M_{\oplus}$, an atmospheric composition of 10% N_2_ and 90%CO_2_, and an incident XUV flux of $8\,F_{\mathrm{XUV},\oplus}$ (i.e.,8 times the XUV surface flux received by the Earth) by Van Looveren et al. (2024) with an exobase level of ∼240 km, Equation (13) gives an exospheric temperature of $\sim 820\,K$ by comparing it Venus. This is very close to the ∼800 K from their simulation. However, at higher XUV fluxes, Equation (13) deviates from the simulations and yields lower temperatures due to the induced expansion and increasing divergences in the upper atmosphere chemistry resulting from the increased XUV surface flux. Equation (13) should therefore be applied with caution.

**Fig. 15:**
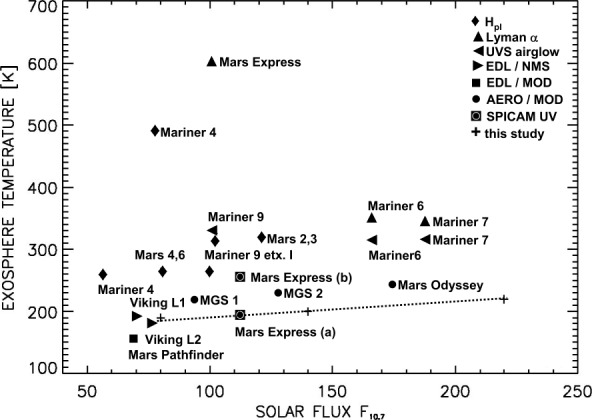
Exosphere daytime temperature estimates based on various methods, data, and space missions during the solar cycle at Mars. The horizontal dashed lines correspond to photochemically produced suprathermal H atoms and CO molecules (adapted from Lichtenegger et al. 2006). The x-axis is the 10.7 cm radio flux (F_10.7_), a commonly used proxy for solar activity

As one can see in Fig. 15, the estimate from Eq. (13) is more accurate than the early exobase temperature estimates inferred from plasma measurements (Mariner 4, Mariner 9, Mars 2, Mars 3, Mars 4, Mars 6), UVS airglow observations (Mariner 6, Mariner 7), and Lyman-$\alpha $ observations of the Martian hydrogen corona by Mariner 6, Mariner 7 and Mars Express (Lichtenegger et al. 2006). The reason for these high exosphere temperatures between ≈ 300 – 600 K obtained by the various methods listed above is related to the photochemically produced suprathermal H, O, and CO populations in the exosphere, while the scaling method above focuses on the thermal neutral atmosphere around the exobase level.

For high XUV fluxes, however, CO_2_ molecules will dissociate, which results in less IR cooling, and a hotter thermosphere. In such cases, the exobase will expand to higher altitudes and $g_{\mathrm{exo}}$ will decrease. Under such conditions, Eq. (12) will underestimate the $T_{\mathrm{exo}}^{\mathrm{CO}_{2}}$ value, if the exobase level, and the resulting $g$ is unknown. Therefore, one will not obtain accurate exobase temperatures if one applies Eq. (13) to exoplanets such as those in the Trappist-1 system.

Van Looveren et al. (2024) applied a thermosphere model including photochemistry to Earth-mass planets in the Trappist-1 system and found that CO_2_ molecules photodissociate below an extended exobase level for XUV fluxes $> 2 \times $ that of the Earth. They do so very efficiently for XUV fluxes $> 10 \times $ that of the Earth, producing atomic O and C, amongst others. These dissociation products are much lighter than CO_2_, and are lost more easily. Additionally, the reduction of IR-cooling in the thermosphere yields the aforementioned expansion of the exobase level to higher altitudes.

**Fig. 16:**
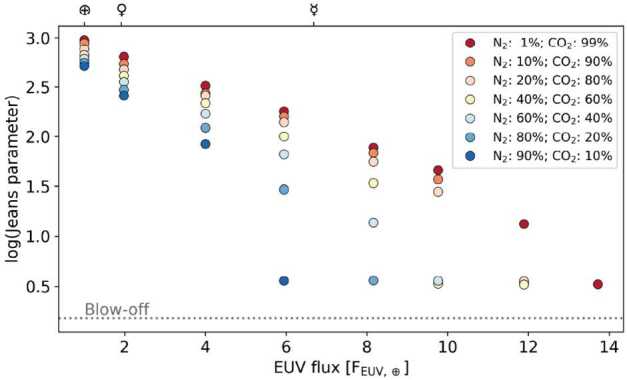
The Jeans parameter $\lambda _{\mathrm{exo}}$ for an Earth-mass planet with various initial atmospheric N_2_/CO_2_ mixing ratios. The horizontal dotted line corresponds to atmospheric blow-off conditions and the x-axis shows the EUV flux in Earth units (adopted from Van Looveren et al. 2024)

As shown in Fig. 16, Van Looveren et al. (2024) calculated the Jeans parameter $\lambda _{\mathrm{exo}}$ (Eq. (3)) at the exobase level for an Earth-mass planet with different atmospheric mixing ratios of N_2_ and CO_2_. Depending on the mixing ratio, one can see that $\lambda _{\mathrm{exo}}$ reaches the conditions for hydrodynamic blow-off (no stable upper atmosphere; see Sect. 2.1.3) for XUV fluxes > 6 times that of today’s Sun at 1 AU. An Earth-mass planet with a 99% CO_2_ atmosphere will reach blow-off escape conditions beyond 14 times present day XUV fluxes at Earth’s orbit (Van Looveren et al. 2024). The findings of Van Looveren et al. (2024) also agree with Tian et al. (2009), who found that early Mars could not build up a dense CO_2_-atmosphere due to the dissociation of CO_2_ molecules at comparably high XUV fluxes (assuming low–moderate outgassing rates). Based on the escape rate evolution from Tian et al. (2009), volcanic degassing cannot counterbalance thermal escape at Mars during the first ∼450 Myr (Scherf and Lammer 2021).

Since the XUV surface fluxes at the planets in the Trappist-1 system^6^ are much higher than the values studied by Van Looveren et al. (2024) stable dense secondary atmospheres could not build up on the Trappist-1 planets (assuming moderate outgassing; see Tian et al. 2009), at least according to their simulations.^7^ This agrees with the tentative non-detections of CO_2_ atmospheres at Trappist-1 b (Greene et al. 2023) and Trappist-1 c (Zieba et al. 2023) and other low mass M-star planets (although issues have been raised about the veracity of these observations; see Fauchez et al. 2025). Note, however, that the existence of atmospheres on the Trappist-1 planets is still disputed (e.g., Lincowski et al. 2023; Ducrot et al. 2025; Gillon et al. 2025); see also Ducrot et al. (2026), this collection, for details. The aforementioned findings indicate that early Venus and Earth may have encountered similar problems beyond 4 Gyr ago.

### Earth-Type Atmospheres

A modern Earth-type atmosphere is a secondary atmosphere composed of N_2_ and O_2_ as the major atmospheric species, with CO_2_ as an important trace molecule and potential additional trace species such as O_3_ and N_2_O (see, e.g., definition of Earth-like atmosphere in Lammer et al. 2024). Earth is the only planet known so far that hosts such an atmosphere, and it is important to note that Titan’s N_2_-dominated atmosphere cannot be categorized as Earth-type, since (i) the processes that led to its formation are completely different, (ii) its upper atmosphere structure diverges significantly, and (iii) O_2_ and CO_2_ are missing in Titan’s atmosphere in Earth-like abundances (e.g., Scherf et al. 2020; Sproß et al. 2021). If one were to put Titan into Earth’s orbit today, its atmosphere would likely escape into space (Sproß et al. 2021), which highlights that planetary mass and the stellar environment strongly matter for the structure and thermal stability of nitrogen-dominated atmospheres of any kind.

**Fig. 17:**
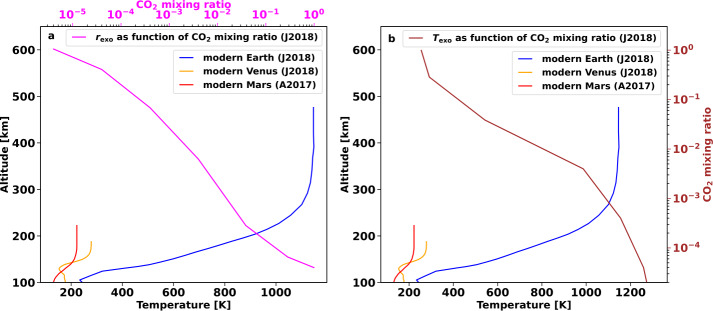
Both panels show the present upper atmosphere temperature profiles of Venus (orange), Earth (blue), and Mars (red), which stop at the exobase for all planets. Panel (a) additionally shows the exobase level, $r_{\mathrm{exo}}$ (magenta), as a function of the CO_2_ to N_2_ mixing ratio for Earth-like conditions (i.e., for the present $F_{\mathrm{XUV}}$ at the Earth’s orbit and its planetary mass of 1.0 M_⊕_). Panel (b) shows the exobase temperature, $T_{\mathrm{exo}}$ (brown), again as a function of CO_2_ to N_2_ mixing ratio for the same Earth-like conditions. One can see how $r_{\mathrm{exo}}$ and $T_{\mathrm{exo}}$ decrease for increasing CO_2_ mixing ratios. Data from Johnstone et al. (2018) and Amerstorfer et al. (2017)

Earth-type atmospheres are generally regarded to be a product of life interacting with the planet as both N_2_ and O_2_ are not simultaneously stable in an atmosphere (e.g., Lovelock and Whitfield 1982; Stüeken et al. 2016; Sproß et al. 2021). A high N_2_ to CO_2_ mixing ratio may be an additional hint of tectonic activity and a working carbon-silicate cycle being present at the respective planet (e.g., Mikhail and Sverjensky 2014; Lammer et al. 2019), which makes the potential detection of such an atmosphere at an exoplanet – in contrast to CO_2_-dominated atmospheres – not only a potential bio- but also a geo-signature. Another important difference between Earth-type and CO_2_-dominated upper atmospheres is their thermal structure since CO_2_ is an IR-coolant and N_2_ is not. While the CO_2_-dominated atmospheres are relatively compact and cool under hydrostatic conditions, Earth-type atmospheres are more extended and exhibit much higher thermospheric temperatures with $r_{\mathrm{exo}}> 400\text{ km}$ and $T_{\mathrm{exo}}>1000\text{ K}$. This is illustrated in Fig. 17, which compares the upper atmosphere thermal structures of Venus, Earth, and Mars. The left panel additionally shows how the exobase radius, $r_{\mathrm{exo}}$, changes as a function of atmospheric CO_2_ mixing ratio whereas the right panel further illustrates the effect of the CO_2_ mixing ratio on the exobase temperature, $T_{\mathrm{exo}}$. It is important to note that these exobase temperatures and radii were modelled by Johnstone et al. (2018) assuming Earth-like conditions, namely, for a planet with a mass of 1.0 M_⊕_, an XUV surface flux identical to today’s surface flux at 1 AU, and an Earth-type atmosphere but with varying CO_2_ mixing ratios. Therefore, $r_{\mathrm{exo}}$ and $T_{\mathrm{exo}}$ for a CO_2_ mixing ratio of ∼0.96 must slightly deviate from the Martian and Venusian values, as conditions at these planets are rather different from this assumption. Equations (12) and (13) illustrate these deviations mathematically as discussed in Sect. 6.3.

**Fig. 18:**
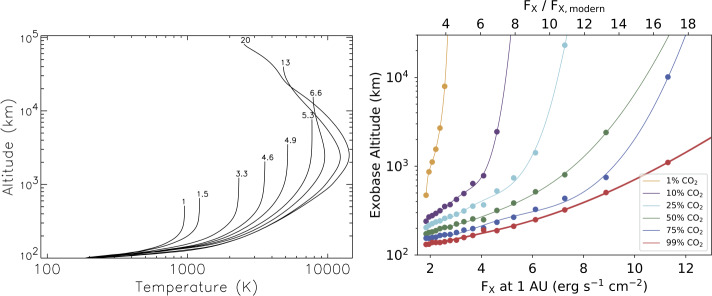
The left panel shows the thermospheric temperature profiles of an Earth-type N_2_-O_2_-dominated atmosphere with 400 ppm CO_2_ for different XUV surface fluxes up to the exobase level, according to the hydrodynamic 1-D model by Tian et al. (2008a). The numbers on top of each profile give the XUV surface flux in present-day values. The right panel illustrates the exobase levels of N_2_-CO_2_-atmospheres as a function of the X-ray surface flux and the CO_2_ mixing ratio simulated with a different upper atmosphere model by Johnstone et al. (2018, 2021b). The various points are simulation results, the lines are fits to the simulations. Left panel adopted from Tian et al. (2008a), right panel adopted from Johnstone et al. (2021b)

If the XUV flux increases, modern Earth-type atmospheres react with an increase in exobase temperature and radius. Tian et al. (2008a) modelled the effect of high XUV fluxes on an Earth-like N_2_-O_2_-dominated atmosphere with 400 ppm CO_2_ and found that $T_{\mathrm{exo}}$ and $r_{\mathrm{exo}}$ increase from ∼900 K to ∼ 8000 K and 500 km to >10 000 km, respectively, for a 6.6-fold increase in the XUV surface flux. The resulting thermospheric temperature profiles from Tian et al. (2008a) are shown in the left panel of Fig. 18. Even if one neglected the atmospheric expansion and the resulting decrease of $g_{\mathrm{exo}}$ in Equation (13), it would still predict a temperature increase to $T_{\mathrm{exo}}\sim 5900\text{ K}$ for the aforementioned 6.6-fold increase in the XUV surface flux. This rough estimate is about 2000 K lower than the modelled value by Tian et al. (2008a) as this also includes the increase of $r_{\mathrm{exo}}$ and the related decrease of $g_{\mathrm{exo}}$. For XUV fluxes above about 6 times the present-day value of $F_{\oplus}$, the Earth-type atmosphere adiabatically expands and escapes hydrodynamically into space. These results indicate that modern Earth-type atmospheres on planets in the HZ of low-mass stars may be strongly disfavoured (see also Fig. 13). However, another recent upper atmosphere model by Nakayama et al. (2022) finds that modern Earth-type atmospheres are much more stable against XUV flux-induced escape due to strong cooling via radiative recombination and enhanced atomic line cooling. It will be an important future collaborative task to intercompare these models, their differences, and different results to understand better the thermal stability of Earth-type atmospheres.

The right panel of Fig. 18 further illustrates the variation of the exobase level of an N_2_-CO_2_-dominated atmosphere as a function of CO_2_ partial pressure and the XUV surface flux as simulated by Johnstone et al. (2021b). One can see that atmospheres with higher CO_2_ mixing ratios are more compact and hence more thermally stable. However, for $F_{\mathrm{XUV}}\gtrapprox \,15\,F_{\oplus}$, even CO_2_-dominated atmospheres experience a strong expansion and significant loss rates as the CO_2_ in the upper atmosphere gets increasingly dissociated. This behaviour also explains the decrease in the Jeans escape parameter, $\lambda _{\mathrm{exo}}$, in Fig. 16; for an increase of the XUV surface flux from 1 to 14 $F_{\oplus}$, $T_{\mathrm{exo}}$ and $r_{\mathrm{exo}}$ increase such that $\lambda _{\mathrm{exo}}$ decreases from 1000 to 0.5 for an atmospheric mixture of 1% N_2_ and 99% CO_2_, indicating hydrodynamic escape of the atmosphere.

Under present-day Earth-like conditions, Earth-type atmospheres are dominated toward the exobase in decreasing order by O, N_2_, and N; the most dominant ions, albeit less abundant than the neutrals, are N^+^, O^+^, and H^+^ (e.g., Picone et al. 2002; Johnstone et al. 2018). Potential biosignature gases (e.g., Seager et al. 2016) such as N_2_O, NH_3_, CH_4_ and even O_2_ and O_3_ are less abundant in the upper atmosphere. Ozone, for instance, is predominantly formed in the stratosphere via the dissociation of O_2_ by UV irradiation where it heats the atmosphere and produces the Earth’s cold trap. However, there is some O_3_ in the lower part of the upper atmosphere (mostly below 100 km), which also contributes to atmospheric heating. Interestingly, O_3_ heating increases over time whereas all other sources of heating in the upper atmosphere decrease with stellar age. This is due to O_3_ absorption at wavelengths >200 nm, a wavelength range that increases with increasing stellar age (Johnstone et al. 2018). For early stellar ages and high XUV fluxes, the abundance of ions in the upper part of modern Earth-type atmospheres will strongly increase. In the model of Nakayama et al. (2022), the ion fraction at the top of the upper atmosphere even reaches 100% for highly irradiated atmospheres. In addition, these authors propose that highly-irradiated, but thermally stable O-rich atmospheres emit strongly in the NUV to optical wavelength range with an intensity of up to $2 \times 10^{20}$ erg s^−1^ This could be detectable during secondary transits in M dwarf systems such as Trappist-1. Current and future exoplanet observations are hence another crucial tool to verify or falsify certain upper atmosphere models and to enhance our knowledge of the stability, evolution, and galactic prevalence of Earth-type atmospheres.

## Role of Planetary Magnetic Fields

There have been many debates on the role of the planetary intrinsic magnetic fields on atmospheric escape (e.g. Lundin et al. 1989; Seki et al. 2001; Ramstad and Barabash 2021; Way et al. 2023). The global magnetic field can deflect most of the stellar wind much further away from the planet and the upper atmosphere is protected by the magnetic barrier. A strong magnetic field results in the formation of a large magnetosphere, which enlarges the area that accumulates stellar wind energy inputs. As introduced in Sect. 2.2, the dominant atmospheric escape mechanisms can change between magnetized and unmagnetized planets. The effects of planetary magnetic fields include those that increase atmospheric escape and those that decrease it, while various factors need to be taken into account to determine whether the net escape rate will increase or decrease. In this section, we review recent progress in our understanding of the role of the planetary intrinsic magnetic fields.

### Insight from Unmagnetized Planets (Mars/Venus) Observations

As mentioned above, there is a lack of consensus on the effects of the intrinsic magnetic field on atmospheric ion escape from terrestrial planets. Although Mars does not have a global intrinsic magnetic field, localized strong crustal magnetic fields exist mainly in its southern hemisphere. Thus, Mars has a built-in control experiment with a localized crustal magnetic field, having both magnetized and unmagnetized regions, and should host various escape processes that occur on both kinds of objects. Observations demonstrate that the induced magnetosphere’s structure is influenced by the crustal magnetic fields, and its effect depends upon the local time of the strong crustal magnetic field region (e.g. Luhmann et al. 2015; Masunaga et al. 2017). Effects of crustal magnetic fields on ion escape from Mars have been investigated by comparing the northern and southern hemispheres.

**Fig. 19:**
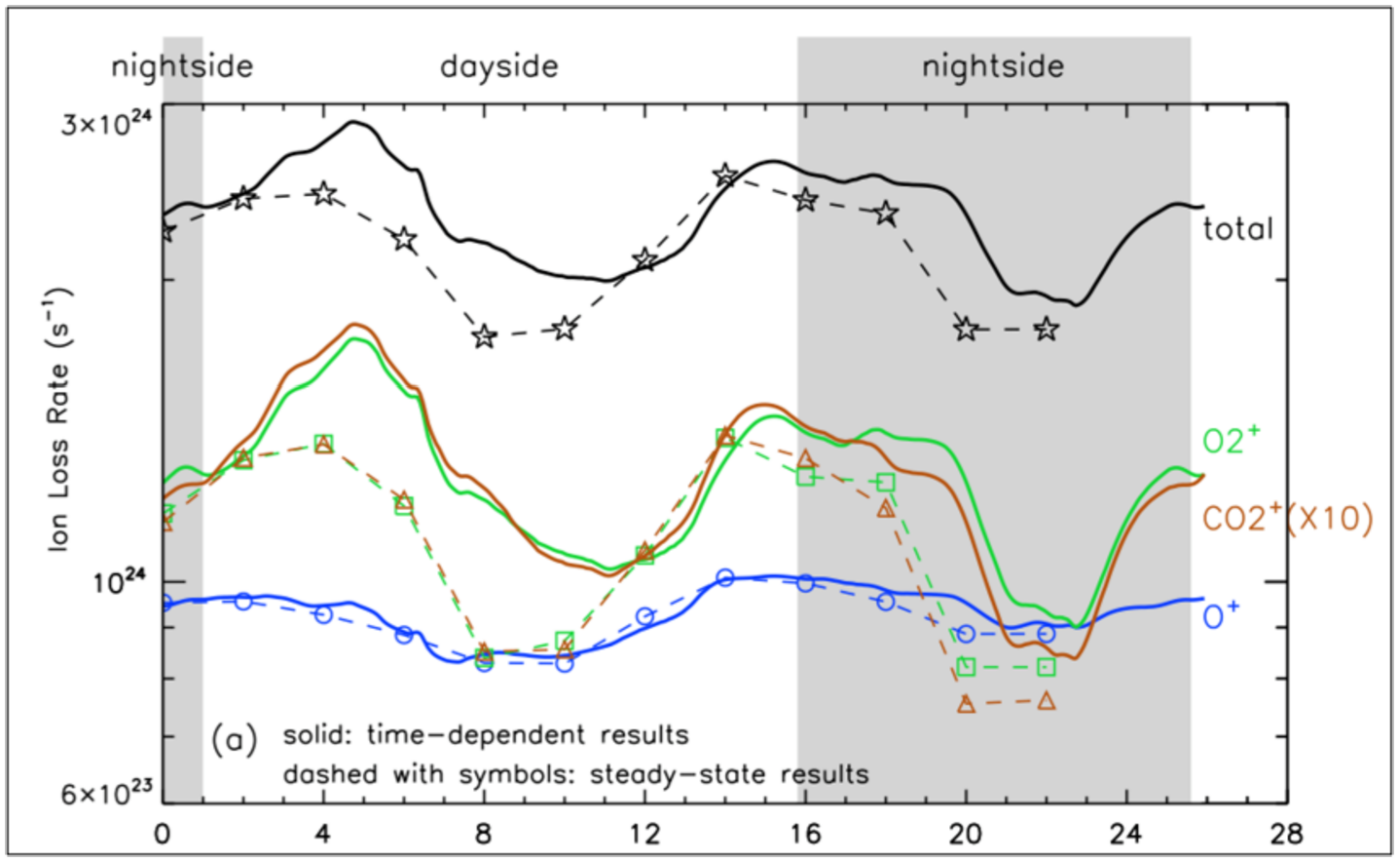
Variation of the ion loss rate from Mars with rotation of the strong crustal magnetic fields region. Image reproduced with permission from Fang et al. (2015), copyright by AGU

Global MHD simulation with the rotating crustal magnetic field on Mars has shown that when simulations include crustal fields, ion loss changes by 0.1-30 times. When simulations rotate crustal fields through a day, ion loss changes by 15-50%, as shown in Fig. 19 (Fang et al. 2015). The simulation results are consistent with observations of the polar plume. Observations also show that solar wind electric field effects are more dominant. Subsolar crustal magnetic fields tend to prevent the formation of the molecular ion plume under low dynamic pressure conditions (Sakakura et al. 2022). As for the tailward escape (see also Sect. 3.1), the escape flux is larger in the -E hemisphere of the MSE coordinate than the +E, and indicates that the solar wind electric field effects are dominant. In the same +E or -E hemisphere, the existence of crustal magnetic fields reduces both the ion escape rate and $\mathrm{O}^{+}/\mathrm{O}_{2}^{+}$ ratio (Inui et al. 2019).

### Insight from Magnetized Planet (Earth) Observations

As introduced in Sect. 2.2 and 3.3, there are different non-thermal escape processes operating in Earth’s magnetosphere (i.e., the polar wind, cusp outflows, auroral outflows, plasma-spheric drainage plumes, and ENA production). The different paths of ion escape depend on the energy, mass, and species, as illustrated in Fig. 11. Even though the Earth’s intrinsic magnetic field reduces the direct access of the solar wind to atmosphere and also traps part of the ion outflow inside the magnetosphere, observational evidences clearly show enhancements in escape for enhanced solar wind energy inputs (Dandouras 2021, and reference therein). These include the dependence of the escape rate of the heavy-ion outflow on solar wind dynamics pressure (Schillings et al. 2019) and geomagnetic activity Slapak et al. (2017) as shown in Fig. 12. Since about half of the incoming solar wind energy input is consumed as Joule Heating in the Earth’s magnetosphere (Tenfjord and Østgaard 2013), it is expected to provide favourable conditions for the escape processes. Magnetospheric dynamics such as magnetospheric convection and substorms/storms due to the enhanced solar wind energy input also increase plasma sheet ion escape from the magnetotail via plasmoid formation and enhance the loss from the dayside magnetopause as observed both for heavy ions (Kronberg et al. 2014, and reference therein) and the cold light ions (Haaland et al. 2012).

During geomagnetic storms, a larger fraction of solar wind energy input is consumed in the ring current (29%, compared to average level of 15% Tenfjord and Østgaard 2013) and contributes to enhanced charge exchange, which is the major loss mechanism, in particular during the late recovery phase of the storm (Keika et al. 2011). Plasmaspheric wind process, on the other hand, is a continuous ion outflow process in equatorial latitude driven by interchange instability of the corotating plasma in the dipole field (André and Lemaire 2006) and was detected under quiet magnetospheric condition (Dandouras 2013). Hence, the relative importance of the different escape processes are expected to vary depending on the state of the magnetosphere, as well as the relative importance of the internal (rotation) and solar wind-driven processes. In the latter context, observations at different solar system planets with different strengths and orientations of the dipole will help predicting possible effects of the magnetospheric processes on the escape at exoplanets. For example, importance of escape due to a loop-like plasmoid in the magnetotail was reported for Uranus (DiBraccio and Gershman 2019), which has a slanted dipole tilt and rotational axis. The observation suggests that both rotation and solar wind are driving the plasma sheet ion escape in the magnetotail, differently from the Earth.

### Insight from Early Earth Studies

Due to the fact that Earth lacks almost any geological record in the Hadean ($\sim 4.6$ to 4 billion years ago), beyond the scarce samples of detrital zircons (Harrison 2009; Lingam and Balbi 2024), it has proven challenging to determine the strength of Earth’s surface magnetic field, or equivalently, its magnetic dipole moment. Hence, it is not surprising that researchers have arrived at distinct, and even opposing, conclusions.

One school of thought holds that Earth has possessed a geomagnetic field strength comparable to that of modern Earth since at least $\sim 4.2$ billion years ago (Ga) based on the analysis of ferromagnetic inclusions in the zircons (Tarduno et al. 2015, 2020), which is best summarized in Fig. 3 of Tarduno et al. (2023). Other groups have contended that, owing to the possibility of these inclusions being incorporated at a later date, it is impossible to establish the existence of the geodynamo prior to 3.5-3.7 Ga (Weiss et al. 2018; Borlina et al. 2020; Taylor et al. 2023). Earth’s paleomagnetic field intensity is better resolved as we move into the Archean (4 to 2.5 Ga), as suggested by Nichols et al. (2024).

However, Earth’s magnetic field has not been constant throughout this period, due to phenomena such as magnetic field reversals. A striking example of this is the evidence for a very weak magnetic field in the Ediacaran Period (defined as 635 to 539 Ma), according to multiple paleomagnetic studies (Bono et al. 2019; Thallner et al. 2021; Domeier et al. 2023). This period is characterised by a magnetic field strength $\sim 30$ times lower than today, and may have lasted for tens of Myr (Huang et al. 2024).

There have been several attempts to try and link the state of Earth’s magnetic field in a particular period to our planet’s habitability by focusing on atmospheric escape rates (e.g. Bono et al. 2019; Huang et al. 2024; Nichols et al. 2024). While there may be potentially significant effects on Earth’s biosphere due to magnetic field changes (cf. Lingam 2019; Pan and Li 2023), the latter’s impact on atmospheric escape may be less important. The reason is that irrespective of the magnitude of Earth’s magnetic field (or dipole moment), the total atmospheric escape rate (in units of kg/s) is predicted to fluctuate only by a factor of $\sim 2$ at most (Gunell et al. 2018). Modelling indicates that these relatively modest variations in the escape rate may not have caused major atmospheric mass or composition fluctuations, thereby mitigating the effects of atmospheric escape on the biosphere in such scenarios (Lingam 2019).

### Predictions from Simulation Studies

**Fig. 20:**
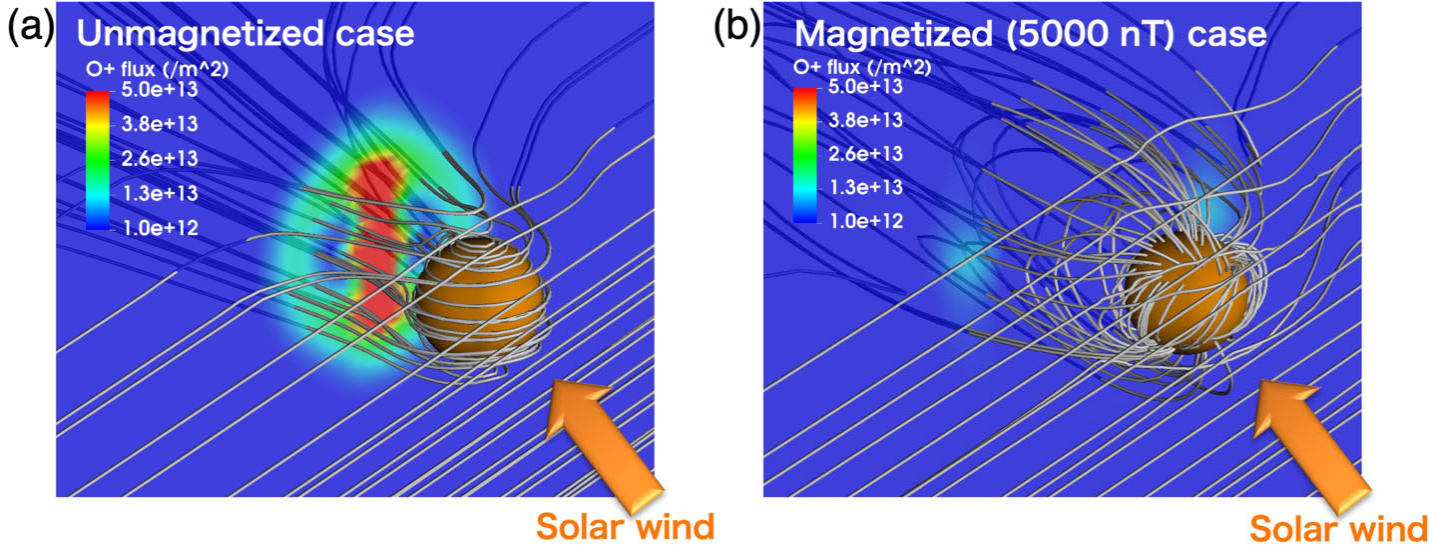
Results of global multi-fluid MHD simulations of the interaction between the solar wind and ancient Mars (a) without and (b) with the global (dipole) intrinsic magnetic field. Lines show the magnetic field lines and the colour contour indicates the tailward escape flux of planetary heavy ions

There have been studies using global hybrid (e.g. Egan et al. 2019), multi-species MHD (e.g. Sakai et al. 2018, 2021, 2023; Sakata et al. 2020, 2022; Dong et al. 2017, 2020; Nishioka et al. 2023), and multi-fluid MHD (Sakata et al. 2024) simulations of the interaction between the stellar wind and magnetized planets. Both hybrid and multi-species MHD simulations indicate that when a planet has a weak magnetic field, it can increase the ion escape rate and the molecular/atomic ion ratio in the ion escape. However, increasing the planetary magnetic field strength leads to decreases in the ion escape rate. This is demonstrated in Fig. 20. Here, the ion escape rate significantly decreases in the strong magnetic field case (i.e., 5000 nT on the equatorial planetary surface, panel b), compared to the unmagnetized case (panel a). The transition of the magnetic field effects on the ion escape rate from increase to decrease depends on the relative strength between the stellar wind dynamic pressure and the magnetic pressure of the planetary dipole field. Namely, a weak intrinsic magnetic field increases ion loss rates when the solar wind dynamic pressure greatly exceeds the magnetic pressure (overpressure). The existence of an intrinsic magnetic field facilitates cusp outflows enabling more escape of molecular ions ($\mathrm{O}_{2}^{+}$, $\mathrm{CO}_{2}^{+}$) (e.g. Sakata et al. 2020). The orientation of the interplanetary magnetic field embedded in the stellar wind can also be an important controlling factor in ion escape (e.g. Sakai et al. 2021, 2023).

**Fig. 21:**
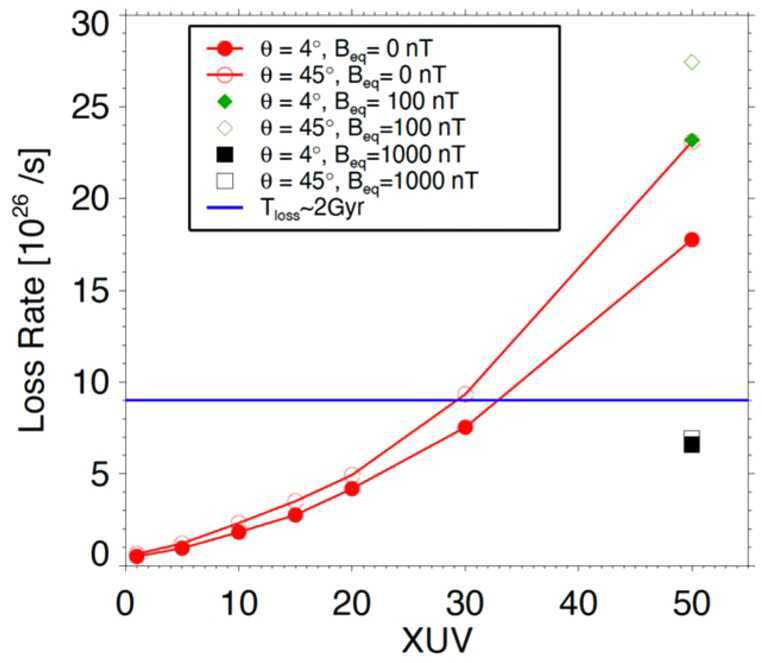
A summary of global MHD simulation results of ion (O^+^) escape from a Venus-like exoplanet at TOI-700 d location. Dependence on stellar XUV radiation is shown for two interplanetary magnetic field orientations (almost radial: $\theta \,=\,4^{\circ}$ and similar to Earth’s location: $\theta \,=\,45^{\circ}$). Green and black symbols show the results of magnetized cases with dipole strength of 100 and 1000 nT at the planetary equatorial surface, respectively (Nishioka et al. 2023)

There have been some studies that simulate ion escape from magnetized exoplanets (e.g. Dong et al. 2017, 2020; Nishioka et al. 2023). A strong intrinsic magnetic field suppresses ion escape, allowing the atmosphere to be retained longer than at unmagnetized planets. For example, as shown with red lines in Fig. 21, stellar XUV flux must be within 30 times that of Earth to retain a modern Venus-like atmosphere for an unmagnetized exoplanet. However, a strong intrinsic magnetic field suppresses ion escape (black symbols in Fig. 21), allowing the atmosphere to be retained even when XUV is 50 times that of Earth (Nishioka et al. 2023).

Similar results were obtained in the MHD simulations of hydrodynamically escaping atmospheres of close-in sub-Neptune-like and giant exoplanets interacting with stellar wind (e.g. Carolan et al. 2021). It was shown that the presence of an intrinsic magnetic field leads to a suppression of the outflow through the magnetotail and the day side while enhancing the outflow through the polar regions. The interplay of those effects leads to an increase in escape rates for weak planetary magnetic fields and the suppression of escape rates for strong ones. It was also shown that for a given planetary magnetic field, the escape increases with the increasing magnetic field strength of the stellar wind (Gupta et al. 2023). These results cannot be directly extrapolated to the escape from young terrestrial planets but suggest that the magnetic field effects during that period (at least, for XUV-driven escape) might be similar to the present day.

## Summary: General Implications for Exoplanetary Systems

Upper atmosphere processes, their relative contribution to volatile losses, and possible consequences for atmospheric evolution depend strongly on planetary parameters such as mass, atmospheric composition, and the presence of a magnetic field, as was at length discussed in Sect. 5, 6, and 7. Local conditions set by the planet’s host star and orbit also play a role (see Sect. 4), in particular, the incident XUV radiation (e.g. Kubyshkina et al. 2018b; Seki et al. 2015), stellar wind properties (e.g. Kislyakova et al. 2014; Curry et al. 2015), and plasma-related phenomena like Coronal Mass Ejections (CMEs; e.g. Hazra et al. 2025) and Stellar Energetic Particles (in particular, for ENA production, e.g. Yue et al. 2019). Observations of the terrestrial planets in the Solar System show that all of these parameters should be considered as an ensemble (see Sect. 3). Effects connected to atmospheric composition, come out differently for planets of different masses and under different irradiation levels (see Sect. 6.3). Meanwhile, many relevant parameters remain poorly constrained for exoplanets and are expected to change drastically throughout their lifetimes (e.g. Emsenhuber et al. 2021). Nevertheless, we can make some qualitative predictions by employing available observations and theoretical modelling, as described below. We note, that these predictions rely on the specific stellar irradiation and atmospheric escape models (i.e., affected by specific model assumptions), and should therefore be considered as an order-of magnitude estimates.

**Fig. 22:**
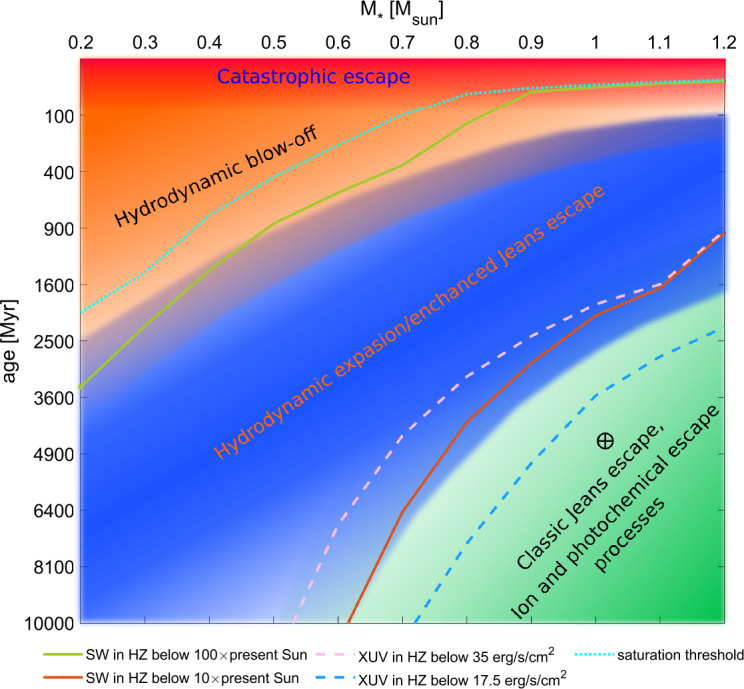
Illustration of thermal and non-thermal atmospheric escape processes as a function of stellar mass and age. Coloured areas indicate the most likely dominant escape processes for the given parameters. The thin cyan dotted line shows the time when the star of a given mass drops out of saturation, while pink and blue dashed lines show the time when XUV radiation in the HZ decreases below the stability threshold of N_2_-dominated atmospheres with a 10% and 1% CO_2_ mixing ratio, respectively (according to Van Looveren et al. 2024). Solid green and brown lines show where the stellar wind flow (density × velocity) decreases below 100 and 10 times that of the present Sun. The “⊕” sign denotes the position of the Earth. All estimates are made assuming the stars to be moderate rotators using the Mors stellar evolution code (Johnstone et al. 2021a) and empirical approximations for the stellar wind strength from Vidotto (2021)

Figure 22 illustrates the expected contribution of the major atmospheric escape processes discussed in this work for planets within the classical liquid water habitable zone where mass versus age is plotted for a given planet’s host star. The highest bulk atmospheric escape rates (red area) are predicted during the stellar activity saturation phase (see 4.2), when the gas disk has just dispersed, and a young (proto)planet is stripped of its surrounding nebula gas. During this phase, the hydrodynamic outflow can be powered by the high internal energy of a newborn planet with inflated atmosphere (e.g. Ginzburg et al. 2018) and extreme stellar heating (e.g. Owen and Wu 2016), with large impacts contributing additional escape (related to the planetary accretion stage that continues after the gas disk dispersal; e.g. Schlichting et al. 2015; Emsenhuber et al. 2021). The escape rates and duration of this phase are mainly constrained by the planet’s mass and size (e.g. Kubyshkina 2022, and references therein). This phase smoothly transitions into the hydrodynamic blow-off phase (orange area). Here, the escape is mostly powered by stellar XUV radiation (e.g. King and Wheatley 2021). The duration of the blow-off phase (∼0.1-2.5 Gyr) depends strongly on the duration of the host star’s saturation phase, and hence on stellar mass and rotation (see discussion in Sect. 4.2). It can spread significantly around the value predicted for a star evolving as moderate rotator shown in Fig. 22. The formation of a stable secondary atmosphere during both of these early phases is unlikely (see e.g. the estimates in Van Looveren et al. 2024).

After hydrodynamic escape begins to cease, the atmosphere of an evolving planet undergoes an efficient thermal escape phase (blue-shaded area in Fig. 22). This is governed by an interplay between a strongly heated and expanded thermosphere-exobase regime, also called enhanced Jeans escape (e.g. Lammer et al. 2008b; Zhu et al. 2014). Whilst the thermosphere expands hydrodynamically upward, adiabatic cooling breaks the upflow before it becomes supersonic, and not all upward-flowing atmospheric atoms reach the escape velocity at the expanded exobase level. At this stage, a significant fractionation of light species is expected. Atmospheres are counterbalanced by outgassing and escape; mass and stellar input still dominate, but the composition of the outgassed atmospheres and magnetic fields become increasingly important (e.g. Lammer 2013; Lammer et al. 2020b).

The transition from enhanced to classical Jeans escape (green area) also depends on the planetary mass and atmospheric composition, controlling adiabatic and IR-cooling in the upper atmosphere (e.g. Johnstone et al. 2018). This transition is illustrated with the pink and blue dashed lines that correspond to the atmospheric stability criteria of atmospheres with N_2_/CO_2_ mixing ratios of 90%/10% and 99%/1% on planets with a mass of 1 M_⊕_ (Van Looveren et al. 2024).

During this latter phase, non-thermal ion and photochemical escape processes can contribute to the total atmospheric mass loss similarly to or more than thermal Jeans escape (e.g. Lammer et al. 2008b). During earlier epochs, only the polar winds on magnetised planets are expected to have escape comparable to the hydrodynamic outflow (e.g. Kislyakova et al. 2020). However, the cumulative effect of the stellar wind-driven non-thermal escape mechanisms (see Table 2) at planets orbiting low-mass stars requires further consideration. The stellar wind flow in the vicinity of such planets may remain more than 10 times the average present-Earth level even after reaching the stability threshold of XUV-driven escape for Earth-like atmospheres (compare pink-dashed and brown solid lines in Fig. 22; Vidotto 2021).

Figure 22 additionally highlights that different atmospheric escape phases generally last longer for stars with lower masses. In particular, HZ planets around stars lighter than ∼0.5 $M_{ \odot}$ may never leave the hydrodynamic/enhanced Jeans escape regime during the main sequence (e.g. Johnstone et al. 2021b). For this reason, atmospheric escape is expected to have a larger impact on the atmospheric evolution of such planets, both in terms of the element fractionation and the stability of the entire atmosphere This may result in the atmospheric composition diversity of M-dwarf planets being heavily shifted towards more stable atmospheres (e.g., CO_2_ or water-rich) instead of those dominated by lighter elements (e.g. Scherf et al. 2024). It can be crucial for planets’ habitability as, for example, nitrogen and oxygen are key elements of amino acids, DNA and RNA. Life processes on Earth are influenced by the atmospheric composition and a change in the relative abundance of these two species can substantially modify biochemical reactions as for example the synthesis of amino acids (Bada 2013; Camprubí et al. 2019). As atmospheric escape shapes the atmospheric composition over time (e.g. Yamauchi and Wahlund 2007; Airapetian et al. 2017), it is essential to evaluate how the composition of the particles that escape responds to changes to the external driving factors, and more specifically how the nitrogen/oxygen escape ratio evolves in response to the solar activity (Yau et al. 1993; Christon et al. 2002; Lin et al. 2024). Nitrogen and oxygen, although having closely-spaced atomic and molecular masses, have very distinct dissociation and ionization energies, and exhibit different responses to varying conditions.

In general, assessing the possible atmospheric compositions of extrasolar planets is strongly limited by our knowledge of the initial volatile budget in the planet’s interior, which can only be loosely constrained using formation models and observations of protoplanetary discs and young systems (e.g. Burn et al. 2024). Thus, even though we can predict atmospheric escape rates, the actual atmospheric lifetime will additionally depend on how long a specific atmosphere can be replenished through outgassing (e.g. Godolt et al. 2019). Furthermore, the initial composition affects the escape rates of specific elements and vice versa. This degeneracy might only be broken with direct observations of planetary atmospheres (e.g. in transmission spectra), which remains challenging even for large planets in close orbits.

Even more problematic is the effect of planetary magnetic fields. While some methods were suggested to measure the magnetic fields of giant exoplanets (e.g. Kavanagh et al. 2024), the prevalence of intrinsic magnetic fields on terrestrial-like exoplanets remains an area of speculation until observational confirmation. As outlined in Sect. 7, the influence of planetary magnetic fields on atmospheric escape is uncertain and depends both on its strength and configuration but also on planetary and stellar parameters. However, even if the effects of the magnetic field on the volatile escape can be negligible on long timescales, its presence is expected to affect potential observational signatures (e.g. Rumenskikh et al. 2025). In particular, planetary magnetospheres’ shape and dynamics can change drastically depending on current stellar wind conditions, stellar periodicities, short-term events, and planetary geomagnetic variability. This can result in highly inhomogeneous outflows.

In this study we do not tackle every factor responsible for atmospheric escape, as such a comprehensive discussion falls outside the scope of the paper. For instance, we do not address the role of the (magnetic) obliquity, which has been shown to exert modest effects on atmospheric escape rates (Dong et al. 2019). We also omit the discussion of planets on highly eccentric or inclined orbits, which can affect star-planet interaction and planetary migration history in various ways (e.g. Emsenhuber et al. 2021). Furthermore, the discussion ignores the presence of atmospheric hazes and dust, which can affect atmospheric opacities, thermal structure (e.g. Lavvas et al. 2019; Adams et al. 2019) and atmospheric pressure gradients (e.g. Schlichting and Young 2022). Our discussion also ignores the role of impacts in atmospheric escape and evolution, which can induce significant losses of primordial atmospheres (e.g., Schlichting et al. 2015; Lammer et al. 2020a; Gillmann et al. 2022) and can be an important factor in the evolution of secondary atmospheres (e.g., Pham et al. 2011; Shorttle et al. 2024). We also omit to discuss the poorly understood effect of electron precipitation; as this heats the lower thermosphere. It could have been highly relevant during the transition period between enhanced and classic Jeans escape (Tian et al. 2008b). Finally, we did not consider in detail the interactions between lower and upper atmospheres (and in particular, the diffusion limit on the volatile supply of upper atmospheres), which are discussed in more detail in Steinmeyer et al. (2026, this collection).

## Appendix: Terms and Abbreviations Used in the Present Study

In Table 3 we summarise the important terms and notations commonly used in the article. For the convenience of the reader, the terms are sorted alphabetically.

**Table 3:** List of terms and notations

Term	Notation	Comment
Astrosphere		The magnetic obstacle formed by the stellar magnetic field’s interaction with the interstellar medium.
Corotation Interaction Region	CIR	The stellar wind plasma structure formed by interaction of the faster wind flow (e.g., originating from coronal holes) with the slower one. Characterised by the forward pressure wave at the leading edge and a reverse pressure wave at the trailing edge (Gosling and Pizzo 1999). It can produce shock waves at large distances from the star.
Energetic neutral atom	ENA	Neutral atmospheric particle with energy sufficient to escape planetary gravitational bound. This energy can be acquired through photochemical reactions or collisions with energetic ions.
Escape velocity	$v_{\mathrm{esc}}$	The minimum speed for an object (atmospheric particle) to escape from a planetary body.
Exobase	_exo_	The atmosphere level where barometric conditions are no longer valid (particle’s mean free path becomes larger than atmospheric scale height).
Exosphere		The uppermost atmospheric layer where no collisions between particles take place.
Extreme Ultraviolet	EUV	Stellar high-energy radiation in 10-91.2 nm wavelength range (most relevant for thermal escape). The upper boundary is set by the atomic hydrogen photoionisation threshold.
Geomagnetic (sub)storm		Short- (order of hours or less) or long-term (up to a few days) perturbations in geomagnetic activity level (defined through Kp, quantifying disturbances in horizontal MF component, AE, auroral electrojet index, and Dst, disturbance storm time), indicating disturuptions in the magnetospheric convection.
Habitable zone	HZ	Here we only consider the definition of the habitable zone as the orbital range where liquid water can be sustained on a planetary surface. For more discussion see, e.g. Scherf et al. (2024).
Heterosphere		The atmospheric layer where molecular diffusion dominates, fractionating lighter species (with a larger scale height, $H_{i}$ from heavier species (with a smaller $H_{i}$). Roughly equivalent to upper atmosphere.
Homosphere		The atmospheric layer where turbulent eddy diffusion dominates, leading to a well mixed bulk gas with a single scale height, *H*. Roughly equivalent to lower atmosphere.
(Magneto)hydrodynamic	(M)HD	The approach describing a given medium (e.g., planetary atmospheres or stellar wind plasma) using fluid dynamic equations. It is applicable as long as the medium remains collisional (mean free path shorter than the local scale height/collisional timescale is much shorter than other timescales in the system).

## Footnotes

1 Fig. 1 is illustrative and combines the data from Zasova et al. (2006) (for Venus), Minzner (1977) (the US Standard Atmosphere, for Earth), Haberle et al. (1999) (for Mars) with the upper atmosphere models from Johnstone et al. (2018) and Amerstorfer et al. (2017).

2 Apart from H, O, C and N, sulphur is the only additional element that could theoretically escape photochemically. However, this can only happen from low-mass bodies with masses below roughly  such as Io. The only photochemically relevant reaction, i.e., the dissociative recombination reaction $\mathrm{SO}^{+} + \mathrm{e} \rightarrow \mathrm{S} + \mathrm{O}$, produces S with an excess energy of 1.66 eV, which is slightly above the escape energy needed for S to escape from Io but already far below the energy needed to escape from Mercury. In addition to S, SO also escapes photochemically from Io via SO_2_^+^ + e → SO + O with an excess energy of 1.66 eV for SO (1.63 eV are needed for SO to escape from Io) (Huang et al. 2023).

3 https://exoplanetarchive.ipac.caltech.edu/

4 https://nssdc.gsfc.nasa.gov/planetary/factsheet/

5 In principle, volcanic degassing could be another atmospheric source. However, volcanic activity on small terrestrial bodies (e.g. Mercury) ceases relatively fast as long as tidal heating can be neglected. Otherwise, volcanic activity can persist as is the case for Io, which hosts an SO_2_-dominated exosphere. For highly active stars even Earth-mass planets can lose their secondary atmospheres and form an exosphere balanced by volcanic degassing and escape into space.

6 The EUV surface fluxes for the Trappist-1 planets derived from the lower X-ray flux values of Wheatley et al. (2017) are 68 (planet h), 120 (planet g), 177 (planet f), 306 (planet e), 529 (planet d), 1050 (planet c), and 1982 (planet b) times the present-day EUV flux, $F_{EUV}$, at the Earth’s orbit with $F_{EUV} = 4.77\text{ erg}\,\mathrm{s}^{-1}\text{ cm}^{-2}$ in the wavelength range 10-121 nm (as calculated by Van Looveren et al. 2024). Here we adopted the “EUV” definition used in Van Looveren et al. (2024), which includes wavelengths up to Ly-$\alpha $ line instead of 91.2 nm.

7 At least for N_2_-O_2_-dominated atmospheres, Nakayama et al. (2022) find much lower thermal escape rates due to the atomic line cooling of O. However, these authors point out that the structure of highly irradiated atmospheres in their model would instead favour increased non-thermal escape rates compared to less strongly irradiated planets. Since non-thermal escape is severe around M dwarfs even without considering expanded atmospheres (e.g., Airapetian et al. 2017; Dong et al. 2018; Garcia-Sage et al. 2017; Rodríguez-Mozos and Moya 2019; Scherf et al. 2024), it can therefore be expected that the existence of dense secondary atmospheres on the Trappist-1 planets remains unlikely even if the simulations by Van Laerhoven et al. (2014) overestimate thermal loss rates. Non-thermal escape can still be critical.

## References

[CR1] Adams D, Gao P, de Pater I, et al. (2019) Aggregate hazes in exoplanet atmospheres. Astrophys J 874(1):61. 10.3847/1538-4357/ab074c. arXiv:1902.05231 [astro-ph.EP]

[CR2] Affolter L, Mordasini C, Oza AV, et al. (2023) Planetary evolution with atmospheric photoevaporation. II. Fitting the slope of the radius valley by combining boil-off and XUV-driven escape. Astron Astrophys 676:A119. 10.1051/0004-6361/202142205. arXiv:2307.02566 [astro-ph.EP]

[CR3] Airapetian VS, Glocer A, Gronoff G, et al. (2016) Prebiotic chemistry and atmospheric warming of early Earth by an active young Sun. Nat Geosci 9(6):452–455. 10.1038/ngeo2719

[CR4] Airapetian VS, Glocer A, Khazanov GV, et al. (2017) How hospitable are space weather affected habitable zones? The role of ion escape. Astrophys J Lett 836(1):L3. 10.3847/2041-8213/836/1/L3

[CR5] Alvarado-Gómez JD, Drake JJ, Cohen O, et al. (2018) Suppression of coronal mass ejections in active stars by an overlying large-scale magnetic field: a numerical study. Astrophys J 862(2):93. 10.3847/1538-4357/aacb7f. arXiv:1806.02828 [astro-ph.SR]

[CR6] Alvarado-Gómez JD, Cohen O, Drake JJ, et al. (2022) Simulating the space weather in the AU mic system: stellar winds and extreme coronal mass ejections. Astrophys J 928(2):147. 10.3847/1538-4357/ac54b8. arXiv:2202.07949 [astro-ph.SR]

[CR7] Amard L, Roquette J, Matt SP (2020) Evidence for metallicity-dependent spin evolution in the Kepler field. Mon Not R Astron Soc 499(3):3481–3493. 10.1093/mnras/staa3038. arXiv:2009.11785 [astro-ph.SR]

[CR8] Amerstorfer UV, Gröller H, Lichtenegger H, et al. (2017) Escape and evolution of Mars’s CO_2_ atmosphere: influence of suprathermal atoms. J Geophys Res Planets 122(6):1321–1337. 10.1002/2016JE005175

[CR9] André N, Lemaire JF (2006) Convective instabilities in the plasmasphere. J Atmos Sol-Terr Phys 68(2):213–227. 10.1016/j.jastp.2005.10.013

[CR10] André M, Crew GB, Peterson WK, et al. (1990) Ion heating by broadband low-frequency waves in the cusp/cleft. J Geophys Res 95(A12):20809–20823. 10.1029/JA095iA12p20809

[CR11] André M, Li K, Eriksson AI (2015) Outflow of low-energy ions and the solar cycle. J Geophys Res Space Phys 120(2):1072–1085. 10.1002/2014JA020714

[CR12] Andreeva VA, Tsyganenko NA (2016) Reconstructing the magnetosphere from data using radial basis functions. J Geophys Res Space Phys 121(3):2249–2263. 10.1002/2015JA022242

[CR13] Arshukova IL, Erkaev NV, Biernat HK, et al. (2004) Interchange instability of the Venusian ionopause. Adv Space Res 33(2):182–186. 10.1016/j.asr.2003.04.015

[CR14] Attia O, Bourrier V, Eggenberger P, et al. (2021) The JADE code: coupling secular exoplanetary dynamics and photo-evaporation. Astron Astrophys 647:A40. 10.1051/0004-6361/202039452. arXiv:2103.02627 [astro-ph.EP]

[CR15] Avice G, Marty B (2020) Perspectives on atmospheric evolution from noble gas and nitrogen isotopes on Earth, Mars & Venus. Space Sci Rev 216(3):36. 10.1007/s11214-020-00655-0. arXiv:2003.11431 [astro-ph.EP]

[CR16] Axford WI (1968) The polar wind and the terrestrial helium budget. J Geophys Res 73(21):6855–6859. 10.1029/JA073i021p06855

[CR17] Bada JL (2013) New insights into prebiotic chemistry from Stanley Miller’s spark discharge experiments. Chem Soc Rev 42(5):2186. 10.1039/c3cs35433d 23340907 10.1039/c3cs35433d

[CR18] Barabash S, Fedorov A, Sauvaud JJ, et al. (2007) The loss of ions from Venus through the plasma wake. Nature 450(7170):650–653. 10.1038/nature06434 18046398 10.1038/nature06434

[CR19] Baraffe I, Selsis F, Chabrier G, et al. (2004) The effect of evaporation on the evolution of close-in giant planets. Astron Astrophys 419:L13–L16. 10.1051/0004-6361:20040129. arXiv:astro-ph/0404101 [astro-ph]

[CR20] Barr AC, Dobos V, Kiss LL (2018) Interior structures and tidal heating in the TRAPPIST-1 planets. Astron Astrophys 613:A37. 10.1051/0004-6361/201731992. arXiv:1712.05641 [astro-ph.EP]

[CR21] Bauer SJ (1971) Solar cycle variation of planetary exospheric temperatures. Nat Phys Sci 232(31):101–102. 10.1038/physci232101a0

[CR22] Bilitza D (2001) International reference ionosphere 2000. Radio Sci 36(2):261–275. 10.1029/2000RS002432

[CR23] Birky J, Barnes R, Fleming DP (2021) Improved constraints for the XUV luminosity evolution of trappist-1. Res Notes Amer Astron Soc 5(5):122. 10.3847/2515-5172/ac034c. arXiv:2105.12562 [astro-ph.EP]

[CR24] Blackledge BW, Green JAM, Barnes R, et al. (2020) Tides on other earths: implications for exoplanet and palaeo-tidal simulations. Geophys Res Lett 47(12):e85746. 10.1029/2019GL085746

[CR25] Bono RK, Tarduno JA, Nimmo F, et al. (2019) Young inner core inferred from Ediacaran ultra-low geomagnetic field intensity. Nat Geosci 12(2):143–147. 10.1038/s41561-018-0288-0

[CR26] Borlina CS, Weiss BP, Lima EA, et al. (2020) Reevaluating the evidence for a hadean-eoarchean dynamo. Sci Adv 6(15):eaav9634. 10.1126/sciadv.aav9634 32284988 10.1126/sciadv.aav9634 PMC7141829

[CR27] Borovsky JE, Denton MH (2008) A statistical look at plasmaspheric drainage plumes. J Geophys Res Space Phys 113(A9):A09221. 10.1029/2007JA012994

[CR28] Borovsky JE, Welling DT, Thomsen MF, et al. (2014) Long-lived plasmaspheric drainage plumes: where does the plasma come from? J Geophys Res Space Phys 119(8):6496–6520. 10.1002/2014JA020228

[CR29] Bower D, Hakim K, Sossi P, et al. (2022) Retention of water in terrestrial magma oceans and carbon-rich early atmospheres. Planet Sci J 3(93):1–28

[CR30] Brace LH, Theis RF, Hoegy WR (1982) Plasma clouds above the ionopause of Venus and their implications. Planet Space Sci 30(1):29–37. 10.1016/0032-0633(82)90069-1

[CR31] Burgasser AJ, Mamajek EE (2017) On the age of the TRAPPIST-1 system. Astrophys J 845(2):110. 10.3847/1538-4357/aa7fea. arXiv:1706.02018 [astro-ph.SR]

[CR32] Burn R, Schlecker M, Mordasini C, et al. (2021) The New Generation Planetary Population Synthesis (NGPPS). IV. Planetary systems around low-mass stars. Astron Astrophys, 656:A72. 10.1051/0004-6361/202140390. arXiv:2105.04596 [astro-ph.EP]

[CR33] Burn R, Mordasini C, Mishra L, et al. (2024) A radius valley between migrated steam worlds and evaporated rocky cores. Nat Astron 8:463–471. 10.1038/s41550-023-02183-7. arXiv:2401.04380 [astro-ph.EP] 38659612 10.1038/s41550-023-02183-7 PMC11035145

[CR34] Buzasi D (2013) Stellar magnetic fields as a heating source for extrasolar giant planets. Astrophys J 765(2):L25. 10.1088/2041-8205/765/2/L25. arXiv:1302.1466 [astro-ph.EP]

[CR35] Caldiroli A, Haardt F, Gallo E, et al. (2021) Irradiation-driven escape of primordial planetary atmospheres. I. The ATES photoionization hydrodynamics code. Astron Astrophys 655:A30. 10.1051/0004-6361/202141497. arXiv:2106.10294 [astro-ph.EP]

[CR36] Camprubí E, de Leeuw JW, House CH, et al. (2019) The emergence of life. Space Sci Rev 215(8):56. 10.1007/s11214-019-0624-8

[CR37] Carolan S, Vidotto AA, Hazra G, et al. (2021) The effects of magnetic fields on observational signatures of atmospheric escape in exoplanets: double tail structures. Mon Not R Astron Soc 508(4):6001–6012. 10.1093/mnras/stab2947. arXiv:2110.05200 [astro-ph.EP]

[CR38] Catling DC, Zahnle KJ (2020) The archean atmosphere. Sci Adv 6(9):eaax1420. 10.1126/sciadv.aax1420. https://www.science.org/doi/pdf/10.1126/sciadv.aax1420 32133393 10.1126/sciadv.aax1420 PMC7043912

[CR39] Cauley PW, Shkolnik EL, Llama J, et al. (2019) Magnetic field strengths of hot Jupiters from signals of star-planet interactions. Nat Astron 3:1128–1134. 10.1038/s41550-019-0840-x. arXiv:1907.09068 [astro-ph.EP]

[CR40] Cecchi-Pestellini C, Ciaravella A, Micela G, et al. (2009) The relative role of EUV radiation and X-rays in the heating of hydrogen-rich exoplanet atmospheres. Astron Astrophys 496(3):863–868. 10.1051/0004-6361/200809955

[CR41] Chaffin MS, Chaufray JY, Deighan J, et al. (2018) Mars H escape rates derived from MAVEN/IUVS Lyman alpha brightness measurements and their dependence on model assumptions. J Geophys Res Planets 123(8):2192–2210. 10.1029/2018JE005574

[CR42] Chamberlain JW (1963) Planetary coronae and atmospheric evaporation. Planet Space Sci 11(8):901–960. 10.1016/0032-0633(63)90122-3

[CR43] Chamberlain JW, Smith GR (1971) Comments on the rate of evaporation of a non-Maxwellian atmosphere. Planet Space Sci 19(7):675–684. 10.1016/0032-0633(71)90025-0

[CR44] Chappell CR (2015) The role of the ionosphere in providing plasma to the terrestrial magnetosphere—an historical overview. Space Sci Rev 192(1–4):5–25. 10.1007/s11214-015-0168-5

[CR45] Charnay B, Le Hir G, Fluteau F, et al. (2017) A warm or a cold early Earth? New insights from a 3-d climate-carbon model. Earth Planet Sci Lett 474:97–109

[CR46] Chen H, Rogers LA (2016) Evolutionary analysis of gaseous sub-Neptune-mass planets with MESA. Astrophys J 831(2):180. 10.3847/0004-637X/831/2/180. arXiv:1603.06596 [astro-ph.EP]

[CR47] Chin L, Dong C, Lingam M (2024) Role of planetary radius on atmospheric escape of rocky exoplanets. Astrophys J 963(1):L20. 10.3847/2041-8213/ad27d8

[CR48] Chirakkil K, Deighan J, Chaffin MS, et al. (2024) EMM EMUS observations of hot oxygen corona at Mars: radial distribution and temporal variability. J Geophys Res Space Phys 129(3):e2023JA032342. 10.1029/2023JA032342

[CR49] Christon SP, Mall U, Eastman TE, et al. (2002) Solar cycle and geomagnetic N^+1^/O^+1^ variation in outer dayside magnetosphere: possible relation to topside ionosphere. Geophys Res Lett 29(5):1058. 10.1029/2001GL013988

[CR50] Cohen O, Kashyap VL, Drake JJ, et al. (2011) The dynamics of stellar coronae harboring hot jupiters. I. A time-dependent magnetohydrodynamic simulation of the interplanetary environment in the HD 189733 planetary system. Astrophys J 733(1):67. 10.1088/0004-637X/733/1/67. arXiv:1101.4825 [astro-ph.SR]

[CR51] Cohen O, Glocer A, Garraffo C, et al. (2024) Heating of the atmospheres of short-orbit exoplanets by their rapid orbital motion through an extreme space environment. Astrophys J 962(2):157. 10.3847/1538-4357/ad206a. arXiv:2401.14459 [astro-ph.EP]

[CR52] Collinson GA, Frahm RA, Glocer A, et al. (2016) The electric wind of Venus: a global and persistent “polar wind”-like ambipolar electric field sufficient for the direct escape of heavy ionospheric ions. Geophys Res Lett 43(12):5926–5934. 10.1002/2016GL068327

[CR53] Correia ACM, Laskar J (2010) Tidal evolution of exoplanets. In: Seager S (ed) Exoplanets. University of Arizona Press, Tucson, pp 239–266. arXiv:1009.1352 [astro-ph.EP]

[CR54] Cravens TE, Gombosi TI, Kozyra J, et al. (1980) Model calculations of the dayside ionosphere of Venus. Energetics 85:7778–7786. 10.1029/JA085iA13p07778

[CR55] Crowley G (1991) Dynamics of the Earth’s thermosphere: a review. Rev Geophys 29:1143–1165

[CR56] Curry SM, Luhmann J, Ma Y, et al. (2015) Comparative pick-up ion distributions at Mars and Venus: consequences for atmospheric deposition and escape. Planet Space Sci 115:35–47. 10.1016/j.pss.2015.03.026

[CR57] Curtis JL, Agüeros MA, Matt SP, et al. (2020) When do stalled stars resume spinning down? Advancing gyrochronology with Ruprecht 147. Astrophys J 904(2):140. 10.3847/1538-4357/abbf58. arXiv:2010.02272 [astro-ph.SR]

[CR58] Dandouras I (2013) Detection of a plasmaspheric wind in the Earth’s magnetosphere by the Cluster spacecraft. Ann Geophys 31(7):1143–1153. 10.5194/angeo-31-1143-2013

[CR59] Dandouras I (2021) Ion outflow and escape in the terrestrial magnetosphere: cluster advances. J Geophys Res Space Phys 126(10):e29753. 10.1029/2021JA029753

[CR60] Dandouras I, Blanc M, Fossati L, et al. (2020) Future missions related to the determination of the elemental and isotopic composition of Earth, Moon and the terrestrial planets. Space Sci Rev 216(8):121. 10.1007/s11214-020-00736-0

[CR61] David TJ, Angus R, Curtis JL, et al. (2022) Further evidence of modified spin-down in sun-like stars: pileups in the temperature-period distribution. Astrophys J 933(1):114. 10.3847/1538-4357/ac6dd3. arXiv:2203.08920 [astro-ph.SR]

[CR62] Dayhoff MO, Eck RV, Lippincott ER, et al. (1967) Venus: atmospheric evolution. Science 155(3762):556–558. 10.1126/science.155.3762.556 17737405 10.1126/science.155.3762.556

[CR63] Delcourt DC, Sauvaud JA, Moore TE (1993) Polar wind ion dynamics in magnetotail. J Geophys Res 98(A6):9155–9170. 10.1029/93JA00301

[CR64] DiBraccio GA, Gershman DJ (2019) Voyager 2 constraints on plasmoid-based transport at Uranus. Geophys Res Lett 46(19):10,710–10,718. 10.1029/2019GL083909

[CR65] do Amaral LNR, Barnes R, Segura A, et al. (2022) The contribution of M-dwarf flares to the thermal escape of potentially habitable planet atmospheres. Astrophys J 928(1):12. 10.3847/1538-4357/ac53af. arXiv:2203.10127 [astro-ph.EP]

[CR66] Dobrovolskis AR (1980) Atmospheric tides and the rotation of Venus II. Spin evolution. Icarus 41(1):18–35

[CR67] Dobrovolskis AR, Ingersoll AP (1980) Atmospheric tides and the rotation of Venus I. Tidal theory and the balance of torques. Icarus 41(1):1–17

[CR68] Domeier M, Robert B, Meert JG, et al. (2023) The enduring Ediacaran paleomagnetic enigma. Earth-Sci Rev 242:104444. 10.1016/j.earscirev.2023.104444

[CR69] Dong Y, Fang X, Brain DA, et al. (2015) Strong plume fluxes at Mars observed by MAVEN: an important planetary ion escape channel. Geophys Res Lett 42(21):8942–8950. 10.1002/2015GL065346

[CR70] Dong Y, Fang X, Brain DA, et al. (2017) Seasonal variability of Martian ion escape through the plume and tail from MAVEN observations. J Geophys Res Space Phys 122(4):4009–4022. 10.1002/2016JA023517

[CR71] Dong C, Lee Y, Ma Y, et al. (2018) Modeling Martian atmospheric losses over time: implications for exoplanetary climate evolution and habitability. Astrophys J 859(1):L14. 10.3847/2041-8213/aac489. arXiv:1805.05016 [astro-ph.EP]

[CR72] Dong C, Huang Z, Lingam M (2019) Role of planetary obliquity in regulating atmospheric escape: G-dwarf versus M-dwarf Earth-like exoplanets. Astrophys J Lett 882(2):L16. 10.3847/2041-8213/ab372c. arXiv:1907.07459 [astro-ph.EP]

[CR73] Dong C, Jin M, Lingam M (2020) Atmospheric escape from TOI-700 d: Venus versus Earth analogs. Astrophys J 896(2):L24. 10.3847/2041-8213/ab982f. arXiv:2005.13190 [astro-ph.EP]

[CR74] Ducrot E, Lagage PO, Min M, et al. (2025) Combined analysis of the 12.8 and 15 μm JWST/MIRI eclipse observations of TRAPPIST-1 b. Nat Astron 9:358–369. 10.1038/s41550-024-02428-z. arXiv:2412.11627 [astro-ph.EP]

[CR75] Ducrot E, Bolmont E, Noack L, et al (2026) Trappist-1: a natural laboratory for the study of temperate rocky exoplanets. Space Sci Rev

[CR76] Ebihara Y, Yamada M, Watanabe S, et al. (2006) Fate of outflowing suprathermal oxygen ions that originate in the polar ionosphere. J Geophys Res Space Phys 111(A4):A04219. 10.1029/2005JA011403

[CR77] Edberg NJT, Nilsson H, Williams AO, et al. (2010) Pumping out the atmosphere of Mars through solar wind pressure pulses. Geophys Res Lett 37(3):L03107. 10.1029/2009GL041814

[CR78] Edberg NJT, Nilsson H, Futaana Y, et al. (2011) Atmospheric erosion of Venus during stormy space weather. J Geophys Res 116:A09308

[CR79] Egan H, Jarvinen R, Ma Y, et al. (2019) Planetary magnetic field control of ion escape from weakly magnetized planets. Mon Not R Astron Soc 488(2):2108–2120. 10.1093/mnras/stz1819. arXiv:1907.02978 [astro-ph.EP]

[CR80] Egger JA, Kubyshkina D, Alibert Y, et al. (2025) Searching for hot water world candidates with CHEOPS: refining the radii and analysing the internal structures and atmospheric lifetimes of TOI-238 b and TOI-1685 b. Astron Astrophys 696:A28. 10.1051/0004-6361/202453325. arXiv:2502.07887 [astro-ph.EP]

[CR81] Elkins-Tanton LT (2011) Formation of early water oceans on rocky planets. Astrophys Space Sci 332(2):359–364. 10.1007/s10509-010-0535-3. arXiv:1011.2710 [astro-ph.EP]

[CR82] Emsenhuber A, Mordasini C, Burn R, et al. (2021) The New Generation Planetary Population Synthesis (NGPPS). I. Bern global model of planet formation and evolution, model tests, and emerging planetary systems. Astron Astrophys 656:A69. 10.1051/0004-6361/202038553. arXiv:2007.05561 [astro-ph.EP]

[CR83] Engwall E, Eriksson AI, Cully CM, et al. (2009) Earth’s ionospheric outflow dominated by hidden cold plasma. Nat Geosci 2(1):24–27. 10.1038/ngeo387

[CR84] Erkaev NV, Kulikov YN, Lammer H, et al. (2007) Roche lobe effects on the atmospheric loss from “hot jupiters”. Astron Astrophys 472(1):329–334. 10.1051/0004-6361:20066929. arXiv:astro-ph/0612729 [astro-ph]

[CR85] Erkaev NV, Lammer H, Odert P, et al. (2015) Extreme hydrodynamic atmospheric loss near the critical thermal escape regime. Mon Not R Astron Soc 448(2):1916–1921. 10.1093/mnras/stv130. arXiv:1506.06592 [astro-ph.EP]

[CR86] Erkaev NV, Lammer H, Odert P, et al. (2016) EUV-driven mass-loss of protoplanetary cores with hydrogen-dominated atmospheres: the influences of ionization and orbital distance. Mon Not R Astron Soc 460(2):1300–1309. 10.1093/mnras/stw935. arXiv:1601.00452 [astro-ph.EP]

[CR87] Erkaev NV, Scherf M, Herbort O, et al. (2023) Modification of the radioactive heat budget of Earth-like exoplanets by the loss of primordial atmospheres. Mon Not R Astron Soc 518(3):3703–3721. 10.1093/mnras/stac3168. arXiv:2209.14691 [astro-ph.EP]

[CR88] Fang X, Ma Y, Brain D, et al. (2015) Control of Mars global atmospheric loss by the continuous rotation of the crustal magnetic field: a time-dependent MHD study. J Geophys Res Space Phys 120(12):10,926–10,944. 10.1002/2015JA021605

[CR89] Fauchez TJ, Rackham BV, Ducrot E, et al. (2025) Stellar models also limit exoplanet atmosphere studies in emission. Astrophys J 989:170. 10.3847/1538-4357/adf068. arXiv:2502.19585

[CR90] Forget F, Wordsworth R, Millour E, et al. (2013) 3d modelling of the early Martian climate under a denser co2 atmosphere: temperatures and co2 ice clouds. Icarus 222(1):81–99

[CR91] Fossati L, Erkaev NV, Lammer H, et al. (2017) Aeronomical constraints to the minimum mass and maximum radius of hot low-mass planets. Astron Astrophys 598:A90. 10.1051/0004-6361/201629716. arXiv:1612.05624 [astro-ph.EP]

[CR92] Fossati L, Young ME, Shulyak D, et al. (2021) Non-local thermodynamic equilibrium effects determine the upper atmospheric temperature structure of the ultra-hot Jupiter KELT-9b. Astron Astrophys 653:A52. 10.1051/0004-6361/202140813. arXiv:2106.11263 [astro-ph.EP]

[CR93] Fowler CM, Hanley KG, McFadden J, et al. (2022) A MAVEN case study of radial IMF at Mars: impacts on the dayside ionosphere. J Geophys Res Space Phys 127(12):e2022JA030726. 10.1029/2022JA030726. https://onlinelibrary.wiley.com/doi/pdf/10.1029/2022JA030726

[CR94] Fox JL, Hać AB (2009) Photochemical escape of oxygen from Mars: a comparison of the exobase approximation to a Monte Carlo method. Icarus 204(2):527–544. 10.1016/j.icarus.2009.07.005

[CR95] Fox JL, Sung KY (2001) Solar activity variations of the Venus thermosphere/ionosphere. J Geophys Res 106(A10):21305–21336. 10.1029/2001JA000069

[CR96] France K, Arulanantham N, Fossati L, et al. (2018) Far-ultraviolet activity levels of F, G, K, and M Dwarf exoplanet host stars. Astrophys J Suppl Ser 239(1):16. 10.3847/1538-4365/aae1a3. arXiv:1809.07342 [astro-ph.SR]

[CR97] Fränz M, Dubinin E, Andrews D, et al. (2015) Cold ion escape from the Martian ionosphere. Planet Space Sci 119:92–102. 10.1016/j.pss.2015.07.012

[CR98] Fulton BJ, Petigura EA (2018) The California-Kepler survey. VII. Precise planet radii leveraging Gaia DR2 reveal the stellar mass dependence of the planet radius gap. Astron J 156(6):264. 10.3847/1538-3881/aae828. arXiv:1805.01453 [astro-ph.EP]

[CR99] Fulton BJ, Petigura EA, Howard AW, et al. (2017) The California-Kepler survey. III. A gap in the radius distribution of small planets. Astron J 154(3):109. 10.3847/1538-3881/aa80eb. arXiv:1703.10375 [astro-ph.EP]

[CR100] Futaana Y, Stenberg Wieser G, Barabash S, et al. (2017) Solar wind interaction and impact on the Venus atmosphere. Space Sci Rev 212(3–4):1453–1509. 10.1007/s11214-017-0362-8

[CR101] García Muñoz A (2007) Physical and chemical aeronomy of HD 209458b. Planet Space Sci 55(10):1426–1455. 10.1016/j.pss.2007.03.007

[CR102] García Muñoz A (2023) Heating and ionization by non-thermal electrons in the upper atmospheres of water-rich exoplanets. Astron Astrophys 672:A77. 10.1051/0004-6361/202245766. arXiv:2308.06026 [astro-ph.EP]

[CR103] García Muñoz A, Schneider PC (2019) Rapid escape of ultra-hot exoplanet atmospheres driven by hydrogen Balmer absorption. Astrophys J 884(2):L43. 10.3847/2041-8213/ab498d. arXiv:1910.00267 [astro-ph.EP]

[CR104] Garcia-Sage K, Glocer A, Drake JJ, et al. (2017) On the magnetic protection of the atmosphere of Proxima Centauri b. Astrophys J 844(1):L13. 10.3847/2041-8213/aa7eca

[CR105] Gillmann C, Way MJ, Avice G, et al. (2022) The long-term evolution of the atmosphere of Venus: processes and feedback mechanisms. Space Sci Rev 218(7):56. 10.1007/s11214-022-00924-0

[CR106] Gillon M, Ducrot E, Bell TJ, et al (2025) First JWST thermal phase curves of temperate terrestrial exoplanets reveal no thick atmosphere around TRAPPIST-1 b and C. arXiv e-prints. 10.48550/arXiv.2509.02128. arXiv:2509.02128 [astro-ph.EP]

[CR107] Ginzburg S, Schlichting HE, Sari R (2018) Core-powered mass-loss and the radius distribution of small exoplanets. Mon Not R Astron Soc 476(1):759–765. 10.1093/mnras/sty290. arXiv:1708.01621 [astro-ph.EP] 10.1093/mnras/stz1230 PMC6750033 31534280

[CR108] Glocer A, Kitamura N, Toth G, et al. (2012) Modeling solar zenith angle effects on the polar wind. J Geophys Res Space Phys 117(A4):A04318. 10.1029/2011JA017136

[CR109] Glocer A, Welling D, Chappell CR, et al. (2020) A case study on the origin of near-Earth plasma. J Geophys Res Space Phys 125(11):e28205. 10.1029/2020JA028205

[CR110] Godolt M, Tosi N, Stracke B, et al. (2019) The habitability of stagnant-lid Earths around dwarf stars. Astron Astrophys 625:A12. 10.1051/0004-6361/201834658. arXiv:1903.07298 [astro-ph.EP]

[CR111] Gordiets BF, Kulikov YN (1985) On the mechanisms of cooling of the nightside thermosphere of Venus. Adv Space Res 5(9):113–117. 10.1016/0273-1177(85)90278-9

[CR112] Gordiets BF, Markov MN, Kulikov IN, et al. (1982) Numerical modelling of the thermospheric heat budget. J Geophys Res 87(A6):4504–4514. 10.1029/JA087iA06p04504

[CR113] Gosling JT, Pizzo VJ (1999) Formation and evolution of corotating interaction regions and their three dimensional structure. Space Sci Rev 89:21–52. 10.1023/A:1005291711900

[CR114] Grayver A, Bower DJ, Saur J, et al. (2022) Interior heating of rocky exoplanets from stellar flares with application to TRAPPIST-1. Astrophys J 941(1):L7. 10.3847/2041-8213/aca287. arXiv:2211.06140 [astro-ph.EP]

[CR115] Greene TP, Bell TJ, Ducrot E, et al. (2023) Thermal emission from the Earth-sized exoplanet TRAPPIST-1 b using JWST. Nature 618(7963):39–42. 10.1038/s41586-023-05951-7 36972683 10.1038/s41586-023-05951-7

[CR116] Gregory BS, Chaffin MS, Elliott RD, et al. (2023) Nonthermal hydrogen loss at Mars: contributions of photochemical mechanisms to escape and identification of key processes. J Geophys Res Planets 128(8):e2023JE007802. 10.1029/2023JE007802. arXiv:2308.13105 [astro-ph.EP]

[CR117] Gronoff G, Arras P, Baraka S, et al. (2020) Atmospheric escape processes and planetary atmospheric evolution. J Geophys Res Space Phys 125(8):e27639. 10.1029/2019JA027639. arXiv:2003.03231 [astro-ph.EP]

[CR118] Gross SH (1972) On the exospheric temperature of hydrogen-dominated planetary atmospheres. J Atmos Sci 29:214–218. 10.1175/1520-0469(1972)029<0214:OTETOH>2.0.CO;2

[CR119] Gu PG, Chen H (2023) Deuterium escape on photoevaporating sub-Neptunes. Astrophys J 953(2):L27. 10.3847/2041-8213/acee01. arXiv:2308.05057 [astro-ph.EP]

[CR120] Güdel M (2020) The Sun through time. Space Sci Rev 216(8):143. 10.1007/s11214-020-00773-9 33328695 10.1007/s11214-020-00773-9 PMC7724955

[CR121] Gunell H, Maggiolo R, Nilsson H, et al. (2018) Why an intrinsic magnetic field does not protect a planet against atmospheric escape. Astron Astrophys 614:L3. 10.1051/0004-6361/201832934

[CR122] Guo JH (2024) Characterization of the regimes of hydrodynamic escape from low-mass exoplanets. Nat Astron 8:920–928. 10.1038/s41550-024-02269-w. arXiv:2405.13283 [astro-ph.EP]

[CR123] Gupta A, Schlichting HE (2019) Sculpting the valley in the radius distribution of small exoplanets as a by-product of planet formation: the core-powered mass-loss mechanism. Mon Not R Astron Soc 487(1):24–33. 10.1093/mnras/stz1230. arXiv:1811.03202 [astro-ph.EP] 31534280 10.1093/mnras/stz1230 PMC6750033

[CR124] Gupta S, Basak A, Nandy D (2023) Impact of changing stellar and planetary magnetic fields on (exo)planetary environments and atmospheric mass loss. Astrophys J 953(1):70. 10.3847/1538-4357/acd93b. arXiv:2303.04770 [astro-ph.EP]

[CR125] Guzewich SD, Way MJ, Aleinov I, et al. (2021) 3d simulations of the early Martian hydrological cycle mediated by a h2-co2 greenhouse. J Geophys Res Planets 126(7):e2021JE006825

[CR126] Haaland S, Eriksson A, Engwall E, et al. (2012) Estimating the capture and loss of cold plasma from ionospheric outflow. J Geophys Res Space Phys 117(A7):A07311. 10.1029/2012JA017679

[CR127] Haberle RM, Joshi MM, Murphy JR, et al. (1999) General circulation model simulations of the Mars Pathfinder atmospheric structure investigation/meteorology data. J Geophys Res 104(E4):8957–8974. 10.1029/1998JE900040

[CR128] Halekas JS (2017) Seasonal variability of the hydrogen exosphere of Mars. J Geophys Res Planets 122(5):901–911. 10.1002/2017JE005306

[CR129] Hall OJ, Davies GR, van Saders J, et al. (2021) Weakened magnetic braking supported by asteroseismic rotation rates of Kepler dwarfs. Nat Astron 5:707–714. 10.1038/s41550-021-01335-x. arXiv:2104.10919 [astro-ph.SR]

[CR130] Hamano K, Abe Y, Genda H (2013) Emergence of two types of terrestrial planet on solidification of magma ocean. Nature 497:607–610. 10.1038/nature12163 23719462 10.1038/nature12163

[CR131] Hara T, Seki K, Futaana Y, et al. (2013) Statistical properties of planetary heavy-ion precipitations toward the Martian ionosphere obtained from Mars Express. J Geophys Res Space Phys 118(8):5348–5357. 10.1002/jgra.50494

[CR132] Hara T, Luhmann JG, Leblanc F, et al. (2017) MAVEN observations on a hemispheric asymmetry of precipitating ions toward the Martian upper atmosphere according to the upstream solar wind electric field. J Geophys Res Space Phys 122(1):1083–1101. 10.1002/2016JA023348

[CR133] Hara T, Luhmann JG, Leblanc F, et al. (2018) Evidence for crustal magnetic field control of ions precipitating into the upper atmosphere of Mars. J Geophys Res Space Phys 123(10):8572–8586. 10.1029/2017JA024798

[CR134] Harrison TM (2009) The Hadean crust: evidence from >4 Ga zircons. Annu Rev Earth Planet Sci 37:479–505. 10.1146/annurev.earth.031208.100151

[CR135] Hartle RE, Grebowsky JM (1990) Upward ion flow in ionospheric holes on Venus. J Geophys Res 95(A1):31–37. 10.1029/JA095iA01p00031

[CR136] Hayakawa H, Bechet S, Clette F, et al. (2023) Magnitude estimates for the Carrington flare in 1859 September: as seen from the original records. Astrophys J 954(1):L3. 10.3847/2041-8213/acd853

[CR137] Hayashi C, Nakazawa K, Mizuno H (1979) Earth’s melting due to the blanketing effect of the primordial dense atmosphere. Earth Planet Sci Lett 43(1):22–28

[CR138] Hazra G, Vidotto AA, Carolan S, et al. (2025) Magnetic interaction of stellar coronal mass ejections with close-in exoplanets: implication on planetary mass-loss and Ly transits. Mon Not R Astron Soc 536(2):1089–1103. 10.1093/mnras/stae2559. arXiv:2411.06283 [astro-ph.SR]

[CR139] Hedin AE, Niemann HB, Kasprzak WT, et al. (1983) Global empirical model of the Venus thermosphere. J Geophys Res 88(A1):73–84. 10.1029/JA088iA01p00073

[CR140] Herbst K, Papaioannou A, Airapetian VS, et al. (2021) From starspots to stellar coronal mass ejections—revisiting empirical stellar relations. Astrophys J 907(2):89. 10.3847/1538-4357/abcc04. arXiv:2011.03761 [astro-ph.SR]

[CR141] Hu R, Seager S, Yung YL (2015) Helium atmospheres on warm Neptune- and sub-Neptune-sized exoplanets and applications to GJ 436b. Astrophys J 807(1):8. 10.1088/0004-637X/807/1/8. arXiv:1505.02221 [astro-ph.EP]

[CR142] Hu R, Bello-Arufe A, Zhang M, et al. (2024) A secondary atmosphere on the rocky exoplanet 55 cancri e. Nature 630(8017):609–612 38718834 10.1038/s41586-024-07432-x

[CR143] Huang X, Gu H, Cui J, et al. (2023) Non-thermal escape of sulfur and oxygen on io driven by photochemistry. J Geophys Res Planets 128(9):e2023JE007811. 10.1029/2023JE007811

[CR144] Huang W, Tarduno JA, Zhou T, et al. (2024) Near-collapse of the geomagnetic field may have contributed to atmospheric oxygenation and animal radiation in the Ediacaran Period. Commun Earth Environ 5(1):207. 10.1038/s43247-024-01360-4

[CR145] Hull AJ, Bonnell JW, Mozer FS, et al. (2003) A statistical study of large-amplitude parallel electric fields in the upward current region of the auroral acceleration region. J Geophys Res Space Phys 108(A1):1007. 10.1029/2001JA007540

[CR146] Hunten DM, Pepin RO, Walker JCG (1987) Mass fractionation in hydrodynamic escape. Icarus 69(3):532–549. 10.1016/0019-1035(87)90022-4

[CR147] Ikoma M, Genda H (2006) Constraints on the mass of a habitable planet with water of nebular origin. Astrophys J 648(1):696–706. 10.1086/505780. arXiv:astro-ph/0606117 [astro-ph]

[CR148] Ikoma M, Elkins-Tanton L, Hamano K, et al. (2018) Water partitioning in planetary embryos and protoplanets with magma oceans. Space Sci Rev 214(4):76. 10.1007/s11214-018-0508-3. arXiv:1804.09294 [astro-ph.EP]

[CR149] Ilie R, Skoug RM, Funsten HO, et al. (2013) The impact of geocoronal density on ring current development. J Atmos Sol-Terr Phys 99:92–103. 10.1016/j.jastp.2012.03.010

[CR150] Ilin E, Poppenhäger K, Chebly J, et al. (2024) Planetary perturbers: flaring star-planet interactions in Kepler and TESS. Mon Not R Astron Soc 527(2):3395–3417. 10.1093/mnras/stad3398. arXiv:2311.04316 [astro-ph.SR]

[CR151] Ilin E, Vedantham HK, Poppenhäger K, et al. (2025) Close-in planet induces flares on its host star. Nature 643(8072):645–648. 10.1038/s41586-025-09236-z. arXiv:2507.00791 [astro-ph.EP] 40604272 10.1038/s41586-025-09236-z

[CR152] Ingersoll AP (1969) The runaway greenhouse: a history of water on Venus. J Atmos Sci 26(6):1191–1198. 10.1175/1520-0469(1969)026<1191:TRGAHO>2.0.CO;2

[CR153] Inui S, Seki K, Namekawa T, et al. (2018) Cold dense ion outflow observed in the Martian-induced magnetotail by MAVEN. Geophys Res Lett 45(11):5283–5289. 10.1029/2018GL077584

[CR154] Inui S, Seki K, Sakai S, et al. (2019) Statistical study of heavy ion outflows from Mars observed in the Martian-induced magnetotail by MAVEN. J Geophys Res Space Phys 124(7):5482–5497. 10.1029/2018JA026452

[CR155] Ito Y, Ikoma M, Kawahara H, et al. (2015) Theoretical emission spectra of atmospheres of hot rocky super-earths. Astrophys J 801(2):144. 10.1088/0004-637X/801/2/144. arXiv:1501.05393 [astro-ph.EP]

[CR156] Jacchia LG, von Slowey JW, Zahn U (1977) Temperature, density, and composition in the disturbed thermosphere from Esro 4 gas analyzer measurements: a global model. J Geophys Res 82(4):684. 10.1029/JA082i004p00684

[CR157] Jackson AP, Davis TA, Wheatley PJ (2012) The coronal X-ray-age relation and its implications for the evaporation of exoplanets. Mon Not R Astron Soc 422(3):2024–2043. 10.1111/j.1365-2966.2012.20657.x. arXiv:1111.0031 [astro-ph.EP]

[CR158] Jakosky BM, Pepin RO, Johnson RE, et al. (1994) Mars atmospheric loss and isotopic fractionation by solar-wind-induced sputtering and photochemical escape. Icarus 111(2):271–288. 10.1006/icar.1994.1145

[CR159] Jakosky BM, Slipski M, Benna M, et al. (2017) Mars’ atmospheric history derived from upper-atmosphere measurements of ^38^Ar/^36^Ar. Science 355(6332):1408–1410. 10.1126/science.aai7721 28360326 10.1126/science.aai7721

[CR160] Jakosky BM, Brain D, Chaffin M, et al. (2018) Loss of the Martian atmosphere to space: present-day loss rates determined from MAVEN observations and integrated loss through time. Icarus 315:146–157. 10.1016/j.icarus.2018.05.030

[CR161] Jarvinen R, Brain DA, Luhmann JG (2016) Dynamics of planetary ions in the induced magnetospheres of Venus and Mars. Planet Space Sci 127:1–14. 10.1016/j.pss.2015.08.012

[CR162] Jarvinen R, Brain DA, Modolo R, et al. (2018) Oxygen ion energization at Mars: comparison of MAVEN and Mars Express observations to global hybrid simulation. J Geophys Res Space Phys 123(2):1678–1689. 10.1002/2017JA024884

[CR163] Jeans JH (1955) The dynamical theory of gases

[CR164] Jin S, Mordasini C (2018) Compositional imprints in density-distance-time: a rocky composition for close-in low-mass exoplanets from the location of the valley of evaporation. Astrophys J 853(2):163. 10.3847/1538-4357/aa9f1e. arXiv:1706.00251 [astro-ph.EP]

[CR165] Johnstone CP, Güdel M, Stökl A, et al. (2015) The evolution of stellar rotation and the hydrogen atmospheres of habitable-zone terrestrial planets. Astrophys J 815(1):L12. 10.1088/2041-8205/815/1/L12. arXiv:1511.03647 [astro-ph.EP]

[CR166] Johnstone CP, Güdel M, Lammer H, et al. (2018) Upper atmospheres of terrestrial planets: carbon dioxide cooling and the Earth’s thermospheric evolution. Astron Astrophys 617:A107. 10.1051/0004-6361/201832776

[CR167] Johnstone CP, Bartel M, Güdel M (2021a) The active lives of stars: a complete description of the rotation and XUV evolution of F, G, K, and M dwarfs. Astron Astrophys 649:A96. 10.1051/0004-6361/202038407. arXiv:2009.07695 [astro-ph.SR]

[CR168] Johnstone CP, Lammer H, Kislyakova KG, et al. (2021b) The young Sun’s XUV-activity as a constraint for lower CO_2_-limits in the Earth’s Archean atmosphere. Earth Planet Sci Lett 576:117197. 10.1016/j.epsl.2021.117197. arXiv:2109.01604 [astro-ph.EP]

[CR169] Kamada A, Kuroda T, Kasaba Y, et al. (2020) A coupled atmosphere–hydrosphere global climate model of early Mars: a ‘cool and wet’ scenario for the formation of water channels. Icarus 338:113567

[CR170] Kamland Collaboration, Gando A, Gando Y, et al. (2011) Partial radiogenic heat model for Earth revealed by geoneutrino measurements. Nat Geosci 4(9):647–651. 10.1038/ngeo1205

[CR171] Kasting JF, Whitmire DP, Reynolds RT (1993) Habitable zones around main sequence stars. Icarus 101(1):108–128. 10.1006/icar.1993.1010 11536936 10.1006/icar.1993.1010

[CR172] Kavanagh RD, Vedantham HK, Rose K, et al. (2024) Unravelling sub-stellar magnetospheres. Astron Astrophys 692:A66. 10.1051/0004-6361/202452094. arXiv:2410.18073 [astro-ph.EP]

[CR173] Kawamura Y, Yoshida T, Terada N, et al. (2024) Reduced water loss due to photochemistry on terrestrial planets in the runaway greenhouse phase around pre-main-sequence M dwarfs. Astrophys J 967(2):95. 10.3847/1538-4357/ad3e7e

[CR174] Kay C, Airapetian VS, Lüftinger T, et al. (2019) Frequency of coronal mass ejection impacts with early terrestrial planets and exoplanets around active solar-like stars. Astrophys J 886(2):L37. 10.3847/2041-8213/ab551f. arXiv:1911.02701 [astro-ph.SR]

[CR175] Kegerreis JA, Eke VR, Massey RJ, et al. (2020) Atmospheric erosion by giant impacts onto terrestrial planets. Astrophys J 897(2):161. 10.3847/1538-4357/ab9810. arXiv:2002.02977 [astro-ph.EP]

[CR176] Keika K, Nosé M, Brandt PC, et al. (2006) Contribution of charge exchange loss to the storm time ring current decay: IMAGE/HENA observations. J Geophys Res Space Phys 111(A11):A11S12. 10.1029/2006JA011789

[CR177] Keika K, Brandt PC, Nosé M, et al. (2011) Evolution of ring current ion energy spectra during the storm recovery phase: implication for dominant ion loss processes. J Geophys Res Space Phys 116:A00J20. 10.1029/2010JA015628

[CR178] King GW, Wheatley PJ (2021) EUV irradiation of exoplanet atmospheres occurs on Gyr time-scales. Mon Not R Astron Soc 501(1):L28–L32. 10.1093/mnrasl/slaa186. arXiv:2007.13731 [astro-ph.EP]

[CR179] King GW, Wheatley PJ, Salz M, et al. (2018) The XUV environments of exoplanets from Jupiter-size to super-Earth. Mon Not R Astron Soc 478(1):1193–1208. 10.1093/mnras/sty1110. arXiv:1804.11124 [astro-ph.EP]

[CR180] Kislyakova K, Noack L (2020) Electromagnetic induction heating as a driver of volcanic activity on massive rocky planets. Astron Astrophys 636:L10. 10.1051/0004-6361/202037924. arXiv:2004.14041 [astro-ph.EP]

[CR181] Kislyakova KG, Johnstone CP, Odert P, et al. (2014) Stellar wind interaction and pick-up ion escape of the Kepler-11 “super-Earths”. Astron Astrophys 562:A116. 10.1051/0004-6361/201322933. arXiv:1312.4721 [astro-ph.EP]

[CR182] Kislyakova KG, Noack L, Johnstone CP, et al. (2017) Magma oceans and enhanced volcanism on TRAPPIST-1 planets due to induction heating. Nat Astron 1:878–885. 10.1038/s41550-017-0284-0. arXiv:1710.08761 [astro-ph.EP]

[CR183] Kislyakova KG, Fossati L, Johnstone CP, et al. (2018) Effective induction heating around strongly magnetized stars. Astrophys J 858(2):105. 10.3847/1538-4357/aabae4. arXiv:1804.06346 [astro-ph.EP]

[CR184] Kislyakova KG, Johnstone CP, Scherf M, et al. (2020) Evolution of the Earth’s polar outflow from mid-archean to present. J Geophys Res Space Phys 125(8):e27837. 10.1029/2020JA027837. arXiv:2008.10337 [astro-ph.EP]

[CR185] Kistler LM, Mouikis CG, Klecker B, et al. (2010) Cusp as a source for oxygen in the plasma sheet during geomagnetic storms. J Geophys Res Space Phys 115(A3):A03209. 10.1029/2009JA014838

[CR186] Kitamura N, Seki K, Nishimura Y, et al. (2015) Limited impact of escaping photoelectrons on the terrestrial polar wind flux in the polar cap. Geophys Res Lett 42(9):3106–3113. 10.1002/2015GL063452

[CR187] Kopparapu RK, Ramirez R, Kasting JF, et al. (2013) Habitable zones around main-sequence stars: new estimates. Astrophys J 765(2):131

[CR188] Koskinen TT, Lavvas P, Huang C, et al. (2022) Mass loss by atmospheric escape from extremely close-in planets. Astrophys J 929(1):52. 10.3847/1538-4357/ac4f45. arXiv:2203.06302 [astro-ph.EP]

[CR189] Krissansen-Totton J, Fortney JJ, Nimmo F (2021) Was Venus ever habitable? Constraints from a coupled interior-atmosphere-redox evolution model. Planet Sci J 2(5):216. 10.3847/PSJ/ac2580. arXiv:2111.00033 [astro-ph.EP]

[CR190] Kronberg EA, Ashour-Abdalla M, Dandouras I, et al. (2014) Circulation of heavy ions and their dynamical effects in the magnetosphere: recent observations and models. Space Sci Rev 184(1–4):173–235. 10.1007/s11214-014-0104-0

[CR191] Kubyshkina D (2022) Stellar rotation and its connection to the evolution of hydrogen-dominated atmospheres of exoplanets. Astron Nachr 343(4):e10077. 10.1002/asna.20210077. arXiv:2111.09653 [astro-ph.EP]

[CR192] Kubyshkina D, Vidotto AA (2021) How does the mass and activity history of the host star affect the population of low-mass planets? Mon Not R Astron Soc 504(2):2034–2050. 10.1093/mnras/stab897. arXiv:2103.13117 [astro-ph.EP]

[CR193] Kubyshkina D, Fossati L, Erkaev NV, et al. (2018a) Overcoming the limitations of the energy-limited approximation for planet atmospheric escape. Astrophys J 866(2):L18. 10.3847/2041-8213/aae586. arXiv:1810.06920 [astro-ph.EP]

[CR194] Kubyshkina D, Fossati L, Erkaev NV, et al. (2018b) Grid of upper atmosphere models for 1-40 M_⊕_ planets: application to CoRoT-7 b and HD 219134 b,c. Astron Astrophys 619:A151. 10.1051/0004-6361/201833737. arXiv:1809.06645 [astro-ph.EP]

[CR195] Kubyshkina D, Fossati L, Mustill AJ, et al. (2019) The Kepler-11 system: evolution of the stellar high-energy emission and initial planetary atmospheric mass fractions. Astron Astrophys 632:A65. 10.1051/0004-6361/201936581. arXiv:1910.09877 [astro-ph.EP]

[CR196] Kubyshkina D, Vidotto AA, Fossati L, et al. (2020) Coupling thermal evolution of planets and hydrodynamic atmospheric escape in MESA. Mon Not R Astron Soc 499(1):77–88. 10.1093/mnras/staa2815. arXiv:2009.04948 [astro-ph.EP]

[CR197] Kubyshkina D, Vidotto AA, Villarreal D’Angelo C, et al. (2022) Atmospheric mass loss and stellar wind effects in young and old systems - II. Is TOI-942 the past of TOI-421 system? Mon Not R Astron Soc 510(2):3039–3045. 10.1093/mnras/stab3620. arXiv:2112.04832 [astro-ph.EP]

[CR198] Kubyshkina D, Fossati L, Erkaev NV (2024) Precise photoionisation treatment and hydrodynamic effects in atmospheric modelling of warm and hot Neptunes. Astron Astrophys 684:A26. 10.1051/0004-6361/202347837. arXiv:2312.07236 [astro-ph.EP]

[CR199] Kulikov YN, Lammer H, Lichtenegger HIM, et al. (2006) Atmospheric and water loss from early Venus. Planet Space Sci 54(13):1425–1444. 10.1016/j.pss.2006.04.021

[CR200] Lainey V (2016) Quantification of tidal parameters from Solar System data. Celest Mech Dyn Astron 126(1-3):145–156. 10.1007/s10569-016-9695-y. arXiv:1604.04184 [astro-ph.EP]

[CR201] Lammer H (2013) Origin and evolution of planetary atmospheres. Springer, Berlin. 10.1007/978-3-642-32087-3

[CR202] Lammer H, Selsis F, Ribas I, et al. (2003) Atmospheric loss of exoplanets resulting from stellar X-ray and extreme-ultraviolet heating. Astrophys J 598(2):L121–L124. 10.1086/380815

[CR203] Lammer H, Lichtenegger HIM, Biernat HK, et al. (2006) Loss of hydrogen and oxygen from the upper atmosphere of Venus. Planet Space Sci 54(13–14):1445–1456. 10.1016/j.pss.2006.04.022

[CR204] Lammer H, Lichtenegger HIM, Kulikov YN, et al. (2007) Coronal Mass Ejection (CME) activity of low mass M stars as an important factor for the habitability of terrestrial exoplanets. II. CME-induced ion pick up of Earth-like exoplanets in close-in habitable zones. Astrobiology 7(1):185–207. 10.1089/ast.2006.0128 17407407 10.1089/ast.2006.0128

[CR205] Lammer H, Kasting JF, Chassefière E, et al. (2008a) Atmospheric escape and evolution of terrestrial planets and satellites. Space Sci Rev 139(1–4):399–436. 10.1007/s11214-008-9413-5

[CR206] Lammer H, Kasting JF, Chassefière E, et al. (2008b) Atmospheric escape and evolution of terrestrial planets and satellites. In: Nagy AF, Balogh A, Cravens TE, et al. (eds) Comparative aeronomy. Space sciences series of ISSI. Springer, New York, pp 399–436. 10.1007/978-0-387-87825-6_11

[CR207] Lammer H, Zerkle AL, Gebauer S, et al. (2018) Origin and evolution of the atmospheres of early Venus, Earth and Mars. Astron Astrophys Rev 26(1):2. 10.1007/s00159-018-0108-y

[CR208] Lammer H, Sproß L, Grenfell JL, et al. (2019) The role of N_2_ as a geo-biosignature for the detection and characterization of Earth-like habitats. Astrobiology 19(7):927–950. 10.1089/ast.2018.1914. arXiv:1904.11716 [astro-ph.EP] 31314591 10.1089/ast.2018.1914

[CR209] Lammer H, Leitzinger M, Scherf M, et al. (2020a) Constraining the early evolution of Venus and Earth through atmospheric Ar, Ne isotope and bulk K/U ratios. Icarus 339:113551. 10.1016/j.icarus.2019.113551

[CR210] Lammer H, Scherf M, Kurokawa H, et al. (2020b) Loss and fractionation of noble gas isotopes and moderately volatile elements from planetary embryos and early Venus, Earth and Mars. Space Sci Rev 216(4):74. 10.1007/s11214-020-00701-x. arXiv:2011.01064 [astro-ph.EP]

[CR211] Lammer H, Brasser R, Johansen A, et al. (2021) Formation of Venus, Earth and Mars: constrained by isotopes. Space Sci Rev 217(1):7. 10.1007/s11214-020-00778-4. arXiv:2102.06173 [astro-ph.EP]

[CR212] Lammer H, Scherf M, Ito Y, et al. (2022) The exosphere as a boundary: origin and evolution of airless bodies in the inner Solar System and beyond including planets with silicate atmospheres. Space Sci Rev 218(3):15. 10.1007/s11214-022-00876-5. arXiv:2203.01656 [astro-ph.EP]

[CR213] Lammer H, Scherf M, Sproß L (2024) Eta-Earth revisited I: a formula for estimating the maximum number of Earth-like habitats. Astrobiology 24(10):897–915. 10.1089/ast.2023.0075 39481024 10.1089/ast.2023.0075

[CR214] Lammer H, Scherf M, Erkaev NV, et al. (2025) Earth-mass planets with He atmospheres in the habitable zone of Sun-like stars. Nat Astron 9:1022–1030. 10.1038/s41550-025-02550-6 40687773 10.1038/s41550-025-02550-6 PMC12274132

[CR215] Lanza AF (2009) Stellar coronal magnetic fields and star-planet interaction. Astron Astrophys 505(1):339–350. 10.1051/0004-6361/200912367. arXiv:0906.1738 [astro-ph.SR]

[CR216] Lanza AF (2010) Hot Jupiters and the evolution of stellar angular momentum. Astron Astrophys 512:A77. 10.1051/0004-6361/200912789. arXiv:0912.4585 [astro-ph.SR]

[CR217] Lanza AF (2012) Star-planet magnetic interaction and activity in late-type stars with close-in planets. Astron Astrophys 544:A23. 10.1051/0004-6361/201219002. arXiv:1206.5893 [astro-ph.EP]

[CR218] Lanza AF (2013) Star-planet magnetic interaction and evaporation of planetary atmospheres. Astron Astrophys 557:A31. 10.1051/0004-6361/201321790. arXiv:1307.2341 [astro-ph.EP]

[CR219] Lanza AF (2022) The role of interactions between stars and their planets. In: Biazzo K, Bozza V, Mancini L, et al. (eds) Demographics of exoplanetary systems. Lecture notes of the 3rd advanced school on exoplanetary science, pp 85–140. 10.1007/978-3-030-88124-5_2

[CR220] Lavvas P, Koskinen T, Steinrueck ME, et al. (2019) Photochemical hazes in sub-Neptunian atmospheres with a focus on GJ 1214b. Astrophys J 878(2):118. 10.3847/1538-4357/ab204e. arXiv:1905.02976 [astro-ph.EP]

[CR221] Leblanc F, Martinez A, Chaufray JY, et al. (2018) On Mars’s atmospheric sputtering after MAVEN’s first Martian year of measurements. Geophys Res Lett 45(10):4685–4691. 10.1002/2018GL077199

[CR222] Lebrun T, Massol H, ChassefièRe E, et al. (2013) Thermal evolution of an early magma ocean in interaction with the atmosphere. J Geophys Res Planets 118:1155–1176. 10.1002/jgre.20068

[CR223] Lecavelier des Etangs A, Vidal-Madjar A, McConnell JC, et al. (2004) Atmospheric escape from hot Jupiters. Astron Astrophys 418:L1–L4. 10.1051/0004-6361:20040106. arXiv:astro-ph/0403369 [astro-ph]

[CR224] Leconte J, Chabrier G, Baraffe I, et al. (2010) Is tidal heating sufficient to explain bloated exoplanets? Consistent calculations accounting for finite initial eccentricity. Astron Astrophys 516:A64. 10.1051/0004-6361/201014337. arXiv:1004.0463 [astro-ph.EP]

[CR225] Léger A, Grasset O, Fegley B, et al. (2011) The extreme physical properties of the CoRoT-7b super-Earth. Icarus 213(1):1–11. 10.1016/j.icarus.2011.02.004. arXiv:1102.1629 [astro-ph.EP]

[CR226] Lemaire JF (2001) The formation of the light-ion trough and peeling off the plasmasphere. J Atmos Sol-Terr Phys 63(11):1285–1291. 10.1016/S1364-6826(00)00232-7

[CR227] Lemaire JF, Peterson WK, Chang T, et al. (2007) History of kinetic polar wind models and early observations. J Atmos Sol-Terr Phys 69(16):1901–1935. 10.1016/j.jastp.2007.08.011

[CR228] Li K, Wei Y, André M, et al. (2017) Cold ion outflow modulated by the solar wind energy input and tilt of the geomagnetic dipole. J Geophys Res Space Phys 122(10):10,658–10,668. 10.1002/2017JA024642

[CR229] Lichtenegger HIM, Lammer H, Kulikov YN, et al. (2006) Effects of low energetic neutral atoms on Martian and Venusian dayside exospheric temperature estimations. Space Sci Rev 126(1–4):469–501. 10.1007/s11214-006-9082-1

[CR230] Lichtenegger HIM, Dyadechkin S, Scherf M, et al. (2022) Non-thermal escape of the Martian CO_2_ atmosphere over time: constrained by Ar isotopes. Icarus 382:115009. 10.1016/j.icarus.2022.115009. arXiv:2105.09789 [astro-ph.EP]

[CR231] Lillis RJ, Deighan J, Fox JL, et al. (2017) Photochemical escape of oxygen from Mars: first results from MAVEN in situ data. J Geophys Res Space Phys 122(3):3815–3836. 10.1002/2016JA023525

[CR232] Limaye SS, Lebonnois S, Mahieux A, et al. (2017) The thermal structure of the Venus atmosphere: intercomparison of Venus Express and ground based observations of vertical temperature and density profiles. Icarus 294:124–155. 10.1016/j.icarus.2017.04.020

[CR233] Lin MY, Cucho-Padin G, Oliveira P, et al. (2024) Variability of Earth’s ionospheric outflow in response to the dynamic terrestrial exosphere. Front Astron Space Sci 11:1462957. 10.3389/fspas.2024.1462957

[CR234] Lincowski AP, Meadows VS, Zieba S, et al. (2023) Potential atmospheric compositions of TRAPPIST-1 C constrained by JWST/MIRI observations at 15 μm. Astrophys J 955(1):L7. 10.3847/2041-8213/acee02. arXiv:2308.05899 [astro-ph.EP]

[CR235] Lingam M (2019) Revisiting the biological ramifications of variations in Earth’s Magnetic Field. Astrophys J 874(2):L28. 10.3847/2041-8213/ab12eb. arXiv:1904.03353 [astro-ph.EP]

[CR236] Lingam M, Balbi A (2024) From stars to life: a quantitative approach to astrobiology. Cambridge University Press, Cambridge

[CR237] Lingam M, Loeb A (2019) Colloquium: physical constraints for the evolution of life on exoplanets. Rev Mod Phys 91(2):021002. 10.1103/RevModPhys.91.021002. arXiv:1810.02007 [astro-ph.EP]

[CR238] Lingam M, Loeb A (2021) Life in the cosmos: from biosignatures to technosignatures. Harvard University Press, Cambridge

[CR239] Linsky J (2019) Host stars and their effects on exoplanet atmospheres. Lecture Notes in Physics, vol. 955. Springer, Cham. 10.1007/978-3-030-11452-7

[CR240] Linsky JL, Redfield S (2024) Inferring intrinsic stellar EUV and Lyman-alpha fluxes and their effects on exoplanet atmospheres. Space Sci Rev 220(3):32. 10.1007/s11214-024-01064-3

[CR241] Lopez ED, Fortney JJ (2014) Understanding the mass-radius relation for sub-neptunes: radius as a proxy for composition. Astrophys J 792(1):1. 10.1088/0004-637X/792/1/1. arXiv:1311.0329 [astro-ph.EP]

[CR242] Lopez ED, Fortney JJ, Miller N (2012) How thermal evolution and mass-loss sculpt populations of super-earths and sub-neptunes: application to the Kepler-11 system and beyond. Astrophys J 761(1):59. 10.1088/0004-637X/761/1/59. arXiv:1205.0010 [astro-ph.EP]

[CR243] Lourenço DL, Breuer D, Arnould M, et al (2026) Rocky planets as heat engines. Space Sci Rev 222 10.1007/s11214-026-01317-3 PMC13503420 42643347

[CR244] Lovelock JE, Whitfield M (1982) Life span of the biosphere. Nature 296:561–563. 10.1038/296561a0

[CR245] Loyd ROP, Schneider PC, Jackman JAG, et al. (2023) Flares, rotation, activity cycles, and a magnetic star-planet interaction hypothesis for the far-ultraviolet emission of GJ 436. Astron J 165(4):146. 10.3847/1538-3881/acbbc8. arXiv:2302.10259 [astro-ph.SR]

[CR246] Luger R, Barnes R (2015) Extreme water loss and abiotic O2 buildup on planets throughout the habitable zones of M dwarfs. Astrobiology 15(2):119–143. 10.1089/ast.2014.1231. arXiv:1411.7412 [astro-ph.EP] 25629240 10.1089/ast.2014.1231 PMC4323125

[CR247] Luhmann JG, Kozyra JU (1991) Dayside pickup oxygen ion precipitation at Venus and Mars: spatial distributions, energy deposition and consequences. J Geophys Res 96(A4):5457–5467. 10.1029/90JA01753.

[CR248] Luhmann JG, Ledvina SA, Lyon JG, et al. (2006) Venus O^+^ pickup ions: collected PVO results and expectations for Venus Express. Planet Space Sci 54(13–14):1457–1471. 10.1016/j.pss.2005.10.009

[CR249] Luhmann JG, Kasprzak WT, Russell CT (2007) Space weather at Venus and its potential consequences for atmosphere evolution. J Geophys Res Planets 112(E4). 10.1029/2006JE002820

[CR250] Luhmann JG, Dong C, Ma Y, et al. (2015) Implications of MAVEN Mars near-wake measurements and models. Geophys Res Lett 42(21):9087–9094. 10.1002/2015GL066122

[CR251] Lundin R, Zakharov A, Pellinen R, et al. (1989) First measurements of the ionospheric plasma escape from Mars. Nature 341:609–612. 10.1038/341609a0

[CR252] Lundin R, Lammer H, Ribas I (2007) Planetary magnetic fields and solar forcing: implications for atmospheric evolution. Space Sci Rev 129(1–3):245–278. 10.1007/s11214-007-9176-4

[CR253] Lynch BJ, Airapetian VS, DeVore CR, et al. (2019) Modeling a Carrington-scale stellar superflare and coronal mass ejection from ^1^Cet. Astrophys J 880(2):97. 10.3847/1538-4357/ab287e. arXiv:1906.03189 [astro-ph.SR] 32214410 10.3847/1538-4357/ab287e PMC7094772

[CR254] Maggiolo R, Maes L, Cessateur G, et al. (2022) The Earth’s magnetic field enhances solar energy deposition in the upper atmosphere. J Geophys Res Space Phys 127(12):e2022JA030899. 10.1029/2022JA030899

[CR255] Mahaffy PR, Webster CR, Atreya SK, et al. (2013) Abundance and isotopic composition of gases in the Martian atmosphere from the Curiosity rover. Science 341(6143):263–266. 10.1126/science.1237966 23869014 10.1126/science.1237966

[CR256] Mamajek EE (2009) Initial conditions of planet formation: lifetimes of primordial disks. In: Usuda T, Tamura M, Ishii M (eds) Exoplanets and disks: their formation and diversity. American institute of physics conference series, vol 1158. AIP, New York, pp 3–10. 10.1063/1.3215910. arXiv:0906.5011

[CR257] Martinez CF, Cunha K, Ghezzi L, et al. (2019b) A spectroscopic analysis of the California-Kepler survey sample. I. Stellar parameters, planetary radii, and a slope in the radius gap. Astrophys J 875(1):29. 10.3847/1538-4357/ab0d93. arXiv:1903.00174 [astro-ph.EP]

[CR258] Martinez A, Leblanc F, Chaufray JY, et al. (2019a) Influence of extreme ultraviolet irradiance variations on the precipitating ion flux from MAVEN observations. Geophys Res Lett 46(13):7761–7768. 10.1029/2019GL083595

[CR259] Masunaga K, Seki K, Brain DA, et al. (2017) Statistical analysis of the reflection of incident O^+^ pickup ions at Mars: MAVEN observations. J Geophys Res Space Phys 122(4):4089–4101. 10.1002/2016JA023516

[CR260] Masunaga K, Futaana Y, Persson M, et al. (2019) Effects of the solar wind and the solar EUV flux on O+ escape rates from Venus. Icarus 321:379–387. 10.1016/j.icarus.2018.11.017. https://linkinghub.elsevier.com/retrieve/pii/S0019103518304433

[CR261] Masunaga K, Terada N, Leblanc F, et al. (2024) A technique for retrieving the exospheric number density distribution from pickup ion ring distributions. Planet Sci J 5(8):180. 10.3847/PSJ/ad65d4

[CR262] Mauk BH, Allegrini F, Bagenal F, et al. (2020) Energetic neutral atoms from Jupiter’s polar regions. J Geophys Res Space Phys 125(12):e28697. 10.1029/2020JA028697

[CR263] Maurice M, Dasgupta R, Hassanzadeh P (2024) Volatile atmospheres of lava worlds. Astron Astrophys 688:A47. 10.1051/0004-6361/202347749. arXiv:2405.09284

[CR264] McElroy MB, Prather MJ, Rodriguez JM (1982) Escape of hydrogen from Venus. Science 215(4540):1614–1615. 10.1126/science.215.4540.1614 17788486 10.1126/science.215.4540.1614

[CR265] McEnulty TR, Luhmann JG, de Pater I, et al. (2010) Interplanetary coronal mass ejection influence on high energy pick-up ions at Venus. Planet Space Sci 58(14):1784–1791. 10.1016/j.pss.2010.07.019

[CR266] Merryfield WJ, Shizgal BD (1994) Discrete velocity model for an escaping single-component atmosphere. Planet Space Sci 42(5):409–419. 10.1016/0032-0633(94)90130-9

[CR267] Mihalas D, Mihalas BW (1984) Foundations of radiation hydrodynamics. Oxford University Press, New York

[CR268] Mikhail S, Sverjensky DA (2014) Nitrogen speciation in upper mantle fluids and the origin of Earth’s nitrogen-rich atmosphere. Nat Geosci 7(11):816–819. 10.1038/ngeo2271

[CR269] Miller N, Fortney JJ, Jackson B (2009) Inflating and deflating hot jupiters: coupled tidal and thermal evolution of known transiting planets. Astrophys J 702(2):1413–1427. 10.1088/0004-637X/702/2/1413. arXiv:0907.1268 [astro-ph.EP]

[CR270] Minzner RA (1977) The 1976 standard atmosphere and its relationship to earlier standards. Rev Geophys Space Phys 15:375–384. 10.1029/RG015i003p00375

[CR271] Miyake W, Mukai T, Kaya N (1996) On the origins of the upward shift of elevated (bimodal) ion conics in velocity space. J Geophys Res 101(A12):26961–26970. 10.1029/96JA02601

[CR272] Miyazaki Y, Korenaga J (2022) A wet heterogeneous mantle creates a habitable world in the hadean. Nature 603(7899):86–90 35236974 10.1038/s41586-021-04371-9

[CR273] Modi A, Estrela R, Valio A (2023) Impact of M-dwarf stellar wind and photoevaporation on the atmospheric evolution of small planets. Mon Not R Astron Soc 525(4):5168–5179. 10.1093/mnras/stad2557. arXiv:2309.10942 [astro-ph.EP]

[CR274] Morbidelli A (2020) Planet formation by pebble accretion in ringed disks. Astron Astrophys 638:A1. 10.1051/0004-6361/202037983. arXiv:2004.04942 [astro-ph.EP]

[CR275] Mordasini C (2020) Planetary evolution with atmospheric photoevaporation. I. Analytical derivation and numerical study of the evaporation valley and transition from super-Earths to sub-Neptunes. Astron Astrophys 638:A52. 10.1051/0004-6361/201935541. arXiv:2002.02455 [astro-ph.EP]

[CR276] Mordasini C, Alibert Y, Georgy C, et al. (2012) Characterization of exoplanets from their formation. II. The planetary mass-radius relationship. Astron Astrophys 547:A112. 10.1051/0004-6361/201118464. arXiv:1206.3303 [astro-ph.EP]

[CR277] Müller-Wodarg ICF, Forbes JM, Keating GM (2006) The thermosphere of Venus and its exploration by a Venus Express Accelerometer Experiment. Planet Space Sci 54(13–14):1415–1424. 10.1016/j.pss.2006.04.029

[CR278] Murray CD, Dermott SF (1999) Solar System Dynamics. 10.1017/CBO9781139174817

[CR279] Murray-Clay RA, Chiang EI, Murray N (2009) Atmospheric escape from hot Jupiters. Astrophys J 693(1):23–42. 10.1088/0004-637X/693/1/23. arXiv:0811.0006 [astro-ph]

[CR280] Nakamura Y, Leblanc F, Terada N, et al. (2023) Numerical prediction of changes in atmospheric chemical compositions during a solar energetic particle event on Mars. J Geophys Res Space Phys 128(12):e2022JA031250. 10.1029/2022JA031250

[CR281] Nakayama A, Ikoma M, Terada N (2022) Survival of terrestrial N_2_-O_2_ atmospheres in violent XUV environments through efficient atomic line radiative cooling. Astrophys J 937(2):72. 10.3847/1538-4357/ac86ca. arXiv:2210.01460 [astro-ph.EP]

[CR282] Newell PT, Sotirelis T, Wing S (2009) Diffuse, monoenergetic, and broadband aurora: the global precipitation budget. J Geophys Res Space Phys 114(A9):A09207. 10.1029/2009JA014326

[CR283] Nichols CIO, Weiss BP, Eyster A, et al. (2024) Possible eoarchean records of the geomagnetic field preserved in the isua supracrustal belt, southern West Greenland. J Geophys Res, Solid Earth 129(4):e2023JB027706. 10.1029/2023JB02770610.31223/x5sx0v

[CR284] Nilsson H, Barghouthi IA, Slapak R, et al. (2012) Hot and cold ion outflow: spatial distribution of ion heating. J Geophys Res Space Phys 117(A11):A11201. 10.1029/2012JA017974

[CR285] Nilsson H, Zhang Q, Stenberg Wieser G, et al. (2023) Solar cycle variation of ion escape from Mars. Icarus 393:114610. 10.1016/j.icarus.2021.114610

[CR286] Nishioka T, Seki K, Sakata R, et al. (2023) Study of atmospheric ion escape from exoplanet TOI-700 d: Venus analogs. J Geophys Res Space Phys 128(8):e2023JA031405. 10.1029/2023JA031405

[CR287] Odert P, Lammer H, Erkaev NV, et al. (2018) Escape and fractionation of volatiles and noble gases from Mars-sized planetary embryos and growing protoplanets. Icarus 307:327–346. 10.1016/j.icarus.2017.10.031. arXiv:1706.06988 [astro-ph.EP]

[CR288] Ogawa Y, Seki K, Hirahara M, et al. (2008) Coordinated EISCAT Svalbard radar and Reimei satellite observations of ion upflows and suprathermal ions. J Geophys Res Space Phys 113(A5):A05306. 10.1029/2007JA012791

[CR289] Ogilvie GI (2014) Tidal dissipation in stars and giant planets. Annu Rev Astron Astrophys 52:171–210. 10.1146/annurev-astro-081913-035941. arXiv:1406.2207 [astro-ph.SR]

[CR290] Ogilvie GI, Lin DNC (2007) Tidal dissipation in rotating solar-type stars. Astrophys J 661(2):1180–1191. 10.1086/515435. arXiv:astro-ph/0702492 [astro-ph]

[CR291] Öpik EJ (1963) Selective escape of gases? Geophys J 7(4):490–506. 10.1111/j.1365-246X.1963.tb07091.x

[CR292] Orell-Miquel J, Murgas F, Pallé E, et al. (2024) The MOPYS project: a survey of 70 planets in search of extended He I and H atmospheres: no evidence of enhanced evaporation in young planets. Astron Astrophys 689:A179. 10.1051/0004-6361/202449411. arXiv:2404.16732 [astro-ph.EP]

[CR293] O’Rourke JG, Gillmann C, Tackley P (2018) Prospects for an ancient dynamo and modern crustal remanent magnetism on Venus. Earth Planet Sci Lett 502:46–56. 10.1016/j.epsl.2018.08.055

[CR294] Owen JE, Alvarez MA (2016) UV driven evaporation of close-in planets: energy-limited, recombination-limited, and photon-limited flows. Astrophys J 816(1):34. 10.3847/0004-637X/816/1/34. arXiv:1504.07170 [astro-ph.EP]

[CR295] Owen JE, Jackson AP (2012) Planetary evaporation by UV & X-ray radiation: basic hydrodynamics. Mon Not R Astron Soc 425(4):2931–2947. 10.1111/j.1365-2966.2012.21481.x. arXiv:1206.2367 [astro-ph.EP]

[CR296] Owen JE, Schlichting HE (2024) Mapping out the parameter space for photoevaporation and core-powered mass-loss. Mon Not R Astron Soc 528(2):1615–1629. 10.1093/mnras/stad3972. arXiv:2308.00020 [astro-ph.EP]

[CR297] Owen JE, Wu Y (2016) Atmospheres of low-mass planets: the “Boil-off”. Astrophys J 817(2):107. 10.3847/0004-637X/817/2/107. arXiv:1506.02049 [astro-ph.EP]

[CR298] Owen JE, Wu Y (2017) The evaporation valley in the Kepler planets. Astrophys J 847(1):29. 10.3847/1538-4357/aa890a. arXiv:1705.10810 [astro-ph.EP]

[CR299] Oyama VI, Carle GC, Woeller F, et al. (1980) Pioneer Venus gas chromatography of the lower atmosphere of Venus. J Geophys Res 85:7891–7902. 10.1029/JA085iA13p07891

[CR300] Pan Y, Li J (2023) On the biospheric effects of geomagnetic reversals. Natl Sci Rev 10(6):nwad070. 10.1093/nsr/nwad070 37181087 10.1093/nsr/nwad070 PMC10171621

[CR301] Parkinson CD (2002) Photochemistry and radiative transfer studies in the atmospheres of Jupiter and Saturn. PhD thesis, York University, Ontario

[CR302] Penz T, Erkaev NV, Biernat HK, et al. (2004) Ion loss on Mars caused by the Kelvin Helmholtz instability. Planet Space Sci 52(13):1157–1167. 10.1016/j.pss.2004.06.001

[CR303] Penz T, Micela G, Lammer H (2008) Influence of the evolving stellar X-ray luminosity distribution on exoplanetary mass loss. Astron Astrophys 477(1):309–314. 10.1051/0004-6361:20078364

[CR304] Pepin RO (1991) On the origin and early evolution of terrestrial planet atmospheres and meteoritic volatiles. Icarus 92(1):2–79. 10.1016/0019-1035(91)90036-S

[CR305] Persson M, Futaana Y, Fedorov A, et al (2018) H+/O+ Escape Rate Ratio in the Venus Magnetotail and its Dependence on the Solar Cycle. Geophys Res Lett 45(20). 10.1029/2018GL079454. https://onlinelibrary.wiley.com/doi/10.1029/2018GL079454

[CR306] Persson M, Futaana Y, Ramstad R, et al (2020) The Venusian Atmospheric Oxygen Ion Escape: Extrapolation to the Early Solar System. J Geophys Res Planets 125(3). 10.1029/2019JE006336

[CR307] Persson M, Futaana Y, Ramstad R, et al (2021) Global Venus-Solar Wind Coupling and Oxygen Ion Escape. Geophys Res Lett 48(3). 10.1029/2020GL091213

[CR308] Pezzotti C, Attia O, Eggenberger P, et al. (2021) The key impact of the host star’s rotational history on the evolution of TOI-849b. Astron Astrophys 654:L5. 10.1051/0004-6361/202141734. arXiv:2110.09364 [astro-ph.EP]

[CR309] Pham LBS, Karatekin O, Dehant V (2011) Effects of impacts on the atmospheric evolution: comparison between Mars, Earth, and Venus. Planet Space Sci 59:1087–1092. 10.1016/j.pss.2010.11.010

[CR310] Picone JM, Hedin AE, Drob DP, et al. (2002) NRLMSISE-00 empirical model of the atmosphere: statistical comparisons and scientific issues. J Geophys Res Space Phys 107(A12):1468. 10.1029/2002JA009430

[CR311] Poppenhaeger K (2022) Helium absorption in exoplanet atmospheres is connected to stellar coronal abundances. Mon Not R Astron Soc 512(2):1751–1764. 10.1093/mnras/stac507. arXiv:2202.08838 [astro-ph.EP]

[CR312] Qin J (2020) Mars upper atmospheric temperature and atomic oxygen density derived from the O I 130.4 nm emission observed by NASA’s MAVEN mission. Astrophys J 159(5):206. 10.3847/1538-3881/ab7fae

[CR313] Ramstad R, Barabash S (2021) Do intrinsic magnetic fields protect planetary atmospheres from stellar winds? Space Sci Rev 217(2):36. 10.1007/s11214-021-00791-1

[CR314] Reinhold T, Shapiro AI, Solanki SK, et al. (2020) The Sun is less active than other solar-like stars. Science 368(6490):518–521. 10.1126/science.aay3821. arXiv:2005.01401 [astro-ph.SR] 32355029 10.1126/science.aay3821

[CR315] Rème H, Aoustin C, Bosqued JM, et al. (2001) First multispacecraft ion measurements in and near the Earth’s magnetosphere with the identical Cluster ion spectrometry (CIS) experiment. Ann Geophys 19:1303–1354. 10.5194/angeo-19-1303-2001

[CR316] Reza A, Kubyshkina D, Fossati L, et al. (2025) Grid-based exoplanet atmospheric mass-loss predictions via neural networks. Astron Astrophys 694:A88. 10.1051/0004-6361/202452379. arXiv:2502.01510 [astro-ph.EP]

[CR317] Rodríguez-Mozos JM, Moya A (2019) Erosion of an exoplanetary atmosphere caused by stellar winds. Astron Astrophys 630:A52. 10.1051/0004-6361/201935543. arXiv:1908.06695 [astro-ph.EP]

[CR318] Rogers JG, Schlichting HE, Young ED (2024) Fleeting but not forgotten: the imprint of escaping hydrogen atmospheres on super-Earth interiors. Astrophys J 970(1):47. 10.3847/1538-4357/ad5287. arXiv:2402.14072 [astro-ph.EP]

[CR319] Rumenskikh M, Taichenachev AV, Shaikhislamov IF, et al. (2025) Probing exoplanetary magnetism via atomic alignment effect. Mon. Not R Astron Soc 541(4):2872–2883. 10.1093/mnras/staf1038. arXiv:2501.01122 [astro-ph.EP]

[CR320] Sakai S, Seki K, Terada N, et al. (2018) Effects of a weak intrinsic magnetic field on atmospheric escape from Mars. Geophys Res Lett 45(18):9336–9343. 10.1029/2018GL079972

[CR321] Sakai S, Seki K, Terada N, et al. (2021) Effects of the IMF direction on atmospheric escape from a Mars like planet under weak intrinsic magnetic field conditions. J Geophys Res Space Phys 126(3):e28485. 10.1029/2020JA028485

[CR322] Sakai S, Seki K, Terada N, et al. (2023) Enhanced ion escape rate during IMF rotation under weak intrinsic magnetic field conditions on a Mars-like planet. J Geophys Res Space Phys 128(3):e2022JA030510. 10.1029/2022JA030510

[CR323] Sakakura K, Seki K, Sakai S, et al. (2022) Formation mechanisms of the molecular ion polar plume and its contribution to ion escape from Mars. J Geophys Res Space Phys 127(6):e29750. 10.1029/2021JA029750

[CR324] Sakata R, Seki K, Sakai S, et al. (2020) Effects of an intrinsic magnetic field on ion loss from ancient Mars based on multispecies MHD simulations. J Geophys Res Space Phys 125(2):e26945. 10.1029/2019JA026945

[CR325] Sakata R, Seki K, Sakai S, et al. (2022) Multispecies MHD study of ion escape at ancient Mars: effects of an intrinsic magnetic field and solar XUV radiation. J Geophys Res Space Phys 127(7):e30427. 10.1029/2022JA030427

[CR326] Sakata R, Seki K, Terada N, et al. (2024) Effects of an intrinsic magnetic field on ion escape from ancient Mars based on MAESTRO multifluid MHD simulations. J Geophys Res Space Phys 129(5):e2023JA032320. 10.1029/2023JA032320

[CR327] Salvador A, Massol H, Davaille A, et al. (2017) The relative influence of h2o and co2 on the primitive surface conditions and evolution of rocky planets. J Geophys Res Planets 122(7):1458–1486

[CR328] Salvador A, Avice G, Breuer D, et al. (2023) Magma ocean, water, and the early atmosphere of Venus. Space Sci Rev 219(7):51

[CR329] Sanz-Forcada J, Micela G, Ribas I, et al. (2011) Estimation of the XUV radiation onto close planets and their evaporation. Astron Astrophys 532:A6. 10.1051/0004-6361/201116594. arXiv:1105.0550 [astro-ph.EP]

[CR330] Saur J, Grambusch T, Duling S, et al. (2013) Magnetic energy fluxes in sub-Alfvénic planet star and moon planet interactions. Astron Astrophys 552:A119. 10.1051/0004-6361/201118179

[CR331] Scherf M, Lammer H (2021) Did Mars possess a dense atmosphere during the irst 400 million years? Space Sci Rev 217(1):2. 10.1007/s11214-020-00779-3

[CR332] Scherf M, Lammer H, Erkaev NV, et al. (2020) Nitrogen atmospheres of the icy bodies in the Solar System. Space Sci Rev 216(8):123. 10.1007/s11214-020-00752-0. arXiv:2011.00973 [astro-ph.EP]

[CR333] Scherf M, Lammer H, Spross L (2024) Eta-Earth revisited II: deriving a maximum number of Earth-like habitats in the galactic disk. Astrobiology 24(10):e916–e1061. 10.1089/ast.2023.0076. arXiv:2412.05002 [astro-ph.EP] 39481023 10.1089/ast.2023.0076

[CR334] Scherf M, Krauss S, Tsurikov G, et al (2025) The impact of electron precipitation on Earth’s thermospheric no production and the drag of Leo satellites. EGUsphere preprint. 10.5194/egusphere-2025-4119

[CR335] Schillings A, Slapak R, Nilsson H, et al. (2019) Earth atmospheric loss through the plasma mantle and its dependence on solar wind parameters. Earth Planets Space 71(1):70. 10.1186/s40623-019-1048-0

[CR336] Schlichting HE, Young ED (2022) Chemical equilibrium between cores, mantles, and atmospheres of super-earths and sub-neptunes and implications for their compositions, interiors, and evolution. Planet Sci J 3(5):127. 10.3847/PSJ/ac68e6. arXiv:2107.10405 [astro-ph.EP]

[CR337] Schlichting HE, Sari R, Yalinewich A (2015) Atmospheric mass loss during planet formation: the importance of planetesimal impacts. Icarus 247:81–94. 10.1016/j.icarus.2014.09.053. arXiv:1406.6435 [astro-ph.EP]

[CR338] Schmidt F, Way MJ, Costard F, et al. (2022) Circumpolar ocean stability on Mars 3 gy ago. Proc Natl Acad Sci USA 119(4):e2112930118 35042794 10.1073/pnas.2112930118 PMC8795497

[CR339] Schrijver K, Bagenal F, Bastian T, et al (2019) Principles of Heliophysics: a textbook on the universal processes behind planetary habitability. 10.48550/arXiv.1910.14022. arXiv e-prints arXiv:1910.14022 [astro-ph.SR]

[CR340] Schulik M, Booth RA (2023) AIOLOS - a multipurpose 1D hydrodynamics code for planetary atmospheres. Mon Not R Astron Soc 523(1):286–304. 10.1093/mnras/stad1251. arXiv:2207.07144 [astro-ph.EP]

[CR341] Schunk RW (2000) Theoretical developments on the causes of ionospheric outflow. J Atmos Sol-Terr Phys 62(6):399–420. 10.1016/S1364-6826(00)00017-1

[CR342] Seager S, Bains W, Petkowski JJ (2016) Toward a list of molecules as potential biosignature gases for the search for life on exoplanets and applications to terrestrial biochemistry. Astrobiology 16(6):465–485. 10.1089/ast.2015.1404 27096351 10.1089/ast.2015.1404

[CR343] Seki K, Elphic RC, Hirahara M, et al. (2001) On atmospheric loss of oxygen ions from Earth through magnetospheric processes. Science 291(5510):1939–1941. 10.1126/science.1058913 11239148 10.1126/science.1058913

[CR344] Seki K, Nagy A, Jackman CM, et al. (2015) A review of general physical and chemical processes related to plasma sources and losses for Solar System magnetospheres. Space Sci Rev 192(1–4):27–89. 10.1007/s11214-015-0170-y

[CR345] Sekiya M, Nakazawa K, Hayashi C (1980) Dissipation of the primordial terrestrial atmosphere due to irradiation of the solar EUV. Prog Theor Phys 64(6):1968–1985. 10.1143/PTP.64.1968

[CR346] Shematovich VI, Ionov DE, Lammer H (2014) Heating efficiency in hydrogen-dominated upper atmospheres. Astron Astrophys 571:A94. 10.1051/0004-6361/201423573. arXiv:1409.0730 [astro-ph.EP]

[CR347] Shibata K, Isobe H, Hillier A, et al. (2013) Can superflares occur on our Sun? Publ Astron Soc Jpn 65(3):49. 10.1093/pasj/65.3.49. arXiv:1212.1361 [astro-ph.SR]

[CR348] Shkolnik E, Bohlender DA, Walker GAH, et al. (2008) The on/off nature of star-planet interactions. Astrophys J 676(1):628–638. 10.1086/527351. arXiv:0712.0004 [astro-ph]

[CR349] Shorttle O, Saeidfirozeh H, Rimmer PB, et al. (2024) Impact sculpting of the early Martian atmosphere. Sci Adv 10(37):eadm9921. 10.1126/sciadv.adm9921 39259790 10.1126/sciadv.adm9921 PMC11639144

[CR350] Skumanich A (1972) Time scales for Ca II emission decay, rotational braking, and lithium depletion. Astrophys J 171:565. 10.1086/151310

[CR351] Slapak R, Nilsson H (2018) The oxygen ion circulation in the outer terrestrial magnetosphere and its dependence on geomagnetic activity. Geophys Res Lett 45(23):12,669–12,676. 10.1029/2018GL079816

[CR352] Slapak R, Schillings A, Nilsson H, et al. (2017) Atmospheric loss from the dayside open polar region and its dependence on geomagnetic activity: implications for atmospheric escape on evolutionary timescales. Ann Geophys 35(3):721–731. 10.5194/angeo-35-721-2017

[CR353] Spada F, Demarque P, Kim YC, et al. (2013) The radius discrepancy in low-mass stars: single versus binaries. Astrophys J 776(2):87. 10.1088/0004-637X/776/2/87. arXiv:1308.5558 [astro-ph.SR]

[CR354] Sproß L, Scherf M, Shematovich VI, et al. (2021) Life as the only reason for the existence of N_2_-O_2_-dominated atmospheres. Astron Rep 65(4):275–296. 10.1134/S1063772921040077. arXiv:2103.09264 [astro-ph.EP]

[CR355] Steinmeyer ML, Noack L, Baumeister P, et al (2026) Evolution and observable properties of rocky planet atmospheres. Space Sci Rev 222 10.1007/s11214-026-01308-4 PMC13350138 42434139

[CR356] Stökl A, Dorfi E, Lammer H (2015) Hydrodynamic simulations of captured protoatmospheres around Earth-like planets. Astron Astrophys 576:A87. 10.1051/0004-6361/201423638. arXiv:1505.06859 [astro-ph.EP]

[CR357] Stüeken EE, Kipp MA, Koehler MC, et al. (2016) Modeling pN_2_ through geological time: implications for planetary climates and atmospheric biosignatures. Astrobiology 16(12):949–963. 10.1089/ast.2016.1537. arXiv:1612.02865 [astro-ph.EP] 27905827 10.1089/ast.2016.1537

[CR358] Stüeken EE, Som SM, Claire M, et al. (2020) Mission to planet Earth: the first two billion years. Space Sci Rev 216(2):31. 10.1007/s11214-020-00652-3

[CR359] Takada M, Seki K, Ogawa Y, et al. (2021) Low altitude ion upflow observed by EISCAT and its effects on supply of molecular ions in the ring current detected by Arase (ERG). J Geophys Res Space Phys 126(5):e28951. 10.1029/2020JA028951

[CR360] Tam SWY, Chang T, Pierrard V (2007) Kinetic modeling of the polar wind. J Atmos Sol-Terr Phys 69(16):1984–2027. 10.1016/j.jastp.2007.08.006

[CR361] Tarduno JA, Cottrell RD, Davis WJ, et al. (2015) A Hadean to Paleoarchean geodynamo recorded by single zircon crystals. Science 349(6247):521–524. 10.1126/science.aaa9114 26228145 10.1126/science.aaa9114

[CR362] Tarduno JA, Cottrell RD, Bono RK, et al. (2020) Paleomagnetism indicates that primary magnetite in zircon records a strong Hadean geodynamo. Proc Natl Acad Sci USA 117(5):2309–2318. 10.1073/pnas.1916553117 31964848 10.1073/pnas.1916553117 PMC7007582

[CR363] Tarduno JA, Cottrell RD, Bono RK, et al. (2023) Hadaean to Palaeoarchaean stagnant-lid tectonics revealed by zircon magnetism. Nature 618:531–536. 10.1038/s41586-023-06024-5 37316722 10.1038/s41586-023-06024-5 PMC10266976

[CR364] Taylor RJ, Reddy SM, Saxey DW, et al. (2023) Direct age constraints on the magnetism of Jack hills zircon. Sci Adv 9(1):eadd1511 36608136 10.1126/sciadv.add1511 PMC9821853

[CR365] Tejada Arevalo RA, Winn JN, Anderson KR (2021) Further evidence for tidal spin-up of hot Jupiter host stars. Astrophys J 919(2):138. 10.3847/1538-4357/ac1429. arXiv:2107.05759 [astro-ph.EP]

[CR366] Tenfjord P, Østgaard N (2013) Energy transfer and flow in the solar wind-magnetosphere-ionosphere system: a new coupling function. J Geophys Res Space Phys 118(9):5659–5672. 10.1002/jgra.50545

[CR367] Thallner D, Biggin AJ, Halls HC (2021) An extended period of extremely weak geomagnetic field suggested by palaeointensities from the Ediacaran Grenville dykes. Earth Planet. Sci. Lett. 568:117025. 10.1016/j.epsl.2021.117025

[CR368] Tian F, Toon OB, Pavlov AA, et al. (2005) A hydrogen-rich early Earth atmosphere. Science 308(5724):1014–1017. 10.1126/science.1106983 15817816 10.1126/science.1106983

[CR369] Tian F, Kasting JF, Liu HL, et al (2008a) Hydrodynamic planetary thermosphere model: 1. Response of the Earth’s thermosphere to extreme solar EUV conditions and the significance of adiabatic cooling. J Geophys Res Planets 113(E5). 10.1029/2007JE002946. https://onlinelibrary.wiley.com/doi/pdf/10.1029/2007JE002946

[CR370] Tian F, Solomon SC, Qian L, et al. (2008b) Hydrodynamic planetary thermosphere model: 2. Coupling of an electron transport/energy deposition model. J Geophys Res Planets 113(E7):E07005. 10.1029/2007JE003043

[CR371] Tian F, Kasting JF, Solomon SC (2009) Thermal escape of carbon from the early Martian atmosphere. Geophys Res Lett 36(2):L02205. 10.1029/2008GL036513

[CR372] Tu L, Johnstone CP, Güdel M, et al. (2015) The extreme ultraviolet and X-ray Sun in time: high-energy evolutionary tracks of a solar-like star. Astron Astrophys 577:L3. 10.1051/0004-6361/201526146. arXiv:1504.04546 [astro-ph.SR]

[CR373] Turbet M, Forget F (2019) The paradoxes of the late hesperian Mars ocean. Sci Rep 9(1):5717 30952959 10.1038/s41598-019-42030-2 PMC6450935

[CR374] Turbet M, Bolmont E, Chaverot G, et al. (2021) Day–night cloud asymmetry prevents early oceans on Venus but not on Earth. Nature 598(7880):276–280 34645997 10.1038/s41586-021-03873-w

[CR375] Van Laerhoven C, Barnes R, Greenberg R (2014) Tides, planetary companions, and habitability: habitability in the habitable zone of low-mass stars. Mon Not R Astron Soc 441(3):1888–1898. 10.1093/mnras/stu685. arXiv:1401.7221 [astro-ph.EP]

[CR376] Van Looveren G, Güdel M, Boro Saikia S, et al. (2024) Airy worlds or barren rocks? On the survivability of secondary atmospheres around the TRAPPIST-1 planets. Astron Astrophys 683:A153. 10.1051/0004-6361/202348079. arXiv:2401.16490 [astro-ph.EP]

[CR377] van Saders JL, Ceillier T, Metcalfe TS, et al. (2016) Weakened magnetic braking as the origin of anomalously rapid rotation in old field stars. Nature 529(7585): 181–184. 10.1038/nature16168. arXiv:1601.02631 [astro-ph.SR] 26727162 10.1038/nature16168

[CR378] Vasilyev V, Reinhold T, Shapiro AI, et al. (2024) Sun-like stars produce superflares roughly once per century. Science 386(6727):1301–1305. 10.1126/science.adl5441. arXiv:2412.12265 [astro-ph.SR] 39666785 10.1126/science.adl5441

[CR379] Venturini J, Guilera OM, Haldemann J, et al. (2020) The nature of the radius valley. Hints from formation and evolution models. Astron Astrophys 643:L1. 10.1051/0004-6361/202039141. arXiv:2008.05513 [astro-ph.EP]

[CR380] Vidotto AA (2021) The evolution of the solar wind. Living Rev Sol Phys 18(1):3. 10.1007/s41116-021-00029-w. arXiv:2103.15748 [astro-ph.SR] 34722865 10.1007/s41116-021-00029-w PMC8550356

[CR381] Vidotto AA (2023) How has the solar wind evolved to become what it is today? In: Cauzzi G, Tritschler A (eds) The era of multi-messenger solar physics, pp 103–109. 10.1017/S1743921322004756. arXiv:2211.15400

[CR382] Volkov AN, Johnson RE, Tucker OJ, et al. (2011) Thermally driven atmospheric escape: transition from hydrodynamic to Jeans Escape. Astrophys J 729(2):L24. 10.1088/2041-8205/729/2/L24. arXiv:1009.5110 [astro-ph.EP]

[CR383] von Zahn U, Fricke KH, Hunten DM, et al. (1980) The upper atmosphere of Venus during morning conditions. J Geophys Res 85:7829–7840. 10.1029/JA085iA13p07829

[CR384] Watson AJ, Donahue TM, Walker JCG (1981) The dynamics of a rapidly escaping atmosphere: applications to the evolution of Earth and Venus. Icarus 48(2):150–166. 10.1016/0019-1035(81)90101-9

[CR385] Way MJ Del Genio AD (2020) Venusian habitable climate scenarios: modeling Venus through time and applications to slowly rotating Venus-like exoplanets. J Geophys Res Planets 125(5):e06276. 10.1029/2019JE00627610.1002/essoar.10501118.3

[CR386] Way MJ, Ostberg C, Foley BJ, et al. (2023) Synergies between Venus & exoplanetary observations. Space Sci Rev 219(1):13. 10.1007/s11214-023-00953-3. arXiv:2302.05718 [astro-ph.EP] 36785654 10.1007/s11214-023-00953-3 PMC9911515

[CR387] Weiss BP, Fu RR, Einsle JF, et al. (2018) Secondary magnetic inclusions in detrital zircons from the Jack Hills, Western Australia, and implications for the origin of the geodynamo. Geology 46(5):427–430. 10.1130/G39938.1

[CR388] Weller MB, Evans AJ, Ibarra DE, et al. (2023) Venus’ atmospheric nitrogen explained by ancient plate tectonics. Nat Astron 7:1436–1444. 10.1038/s41550-023-02102-w

[CR389] Wheatley PJ, Louden T, Bourrier V, et al. (2017) Strong XUV irradiation of the Earth-sized exoplanets orbiting the ultracool dwarf TRAPPIST-1. Mon Not R Astron Soc 465(1):L74–L78. 10.1093/mnrasl/slw192. arXiv:1605.01564 [astro-ph.EP]

[CR390] Wolff RS, Goldstein BE, Yeates CM (1980) The onset and development of Kelvin-Helmholtz instability at the Venus ionopause. J Geophys Res 85:7697–7707. 10.1029/JA085iA13p07697

[CR391] Wordsworth RD (2016) The climate of early Mars. Annu Rev Earth Planet Sci 44(1):381–408

[CR392] Wordsworth R, Pierrehumbert R (2014) Abiotic oxygen-dominated atmospheres on terrestrial habitable zone planets. Astrophys J 785(2):L20. 10.1088/2041-8205/785/2/L20. arXiv:1403.2713 [astro-ph.EP]

[CR393] Wright NJ, Drake JJ, Mamajek EE, et al. (2011) The stellar-activity-rotation relationship and the evolution of stellar dynamos. Astrophys J 743(1):48. 10.1088/0004-637X/743/1/48. arXiv:1109.4634 [astro-ph.SR]

[CR394] Wurz P, Fatemi S, Galli A, et al. (2022) Particles and photons as drivers for particle release from the surfaces of the Moon and Mercury. Space Sci Rev 218(3):10. 10.1007/s11214-022-00875-6

[CR395] Xu Y, Alvarado-Gómez JD, Tian H, et al (2024) Simulated Coronal Mass Ejections on a young Solar-Type Star and the Associated Instantaneous Angular Momentum Loss. 10.48550/arXiv.2406.08194. arXiv:2406.08194 [astro-ph.SR]

[CR396] Yamakawa T, Seki K, Amano T, et al. (2023) Effects of cold plasma on the excitation of internally driven ULF waves by ring current ions based on the magnetosphere-ionosphere coupled model. J Geophys Res Space Phys 128(9):e2023JA031638. 10.1029/2023JA031638

[CR397] Yamauchi M (2019) Terrestrial ion escape and relevant circulation in space. Ann Geophys 37(6):1197–1222. 10.5194/angeo-37-1197-2019

[CR398] Yamauchi M, Wahlund JE (2007) Role of the ionosphere for the atmospheric evolution of planets. Astrobiology 7(5):783–800. 10.1089/ast.2007.0140 17963477 10.1089/ast.2007.0140

[CR399] Yamauchi M, Christon S, Dandouras I, et al. (2024) Heavy molecular and metallic ions in the magnetosphere. Space Sci Rev 220(8):82. 10.1007/s11214-024-01114-w

[CR400] Yau AW, Andre M (1997) Sources of ion outflow in the high latitude ionosphere. Space Sci Rev 80:1–25. 10.1023/A:1004947203046

[CR401] Yau AW, Whalen BA, Goodenough C, et al. (1993) EXOS D (Akebono) observations of molecular NO^+^ and upflowing ions in the high-altitude auroral ionosphere. J Geophys Res 98(A7):11205–11224. 10.1029/92JA02019

[CR402] Yau AW, Abe T, Peterson WK (2007) The polar wind: recent observations. J Atmos Sol-Terr Phys 69(16):1936–1983. 10.1016/j.jastp.2007.08.010

[CR403] Yau AW, Abe T, André M, et al. (2021) Ionospheric ion acceleration and transport. In: Maggiolo R, André N, Hasegawa H, et al. (eds) Magnetospheres in the Solar System. AGU/Wiley, Hoboken, NJ, pp 207–217. 10.1002/9781119815624.ch14

[CR404] Yelle RV (2004) Aeronomy of extra-solar giant planets at small orbital distances. Icarus 170(1):167–179. 10.1016/j.icarus.2004.02.008

[CR405] Yoshida T, Terada N, Ikoma M, et al. (2022) Less effective hydrodynamic escape of H_2_-H_2_O atmospheres on terrestrial planets orbiting pre-main-sequence M dwarfs. Astrophys J 934(2):137. 10.3847/1538-4357/ac7be7. arXiv:2207.06570 [astro-ph.EP]

[CR406] Young DT, Burch JL, Gomez RG, et al. (2016) Hot plasma composition analyzer for the magnetospheric multiscale mission. Space Sci Rev 199(1–4):407–470. 10.1007/s11214-014-0119-6

[CR407] Yue C, Bortnik J, Li W, et al. (2019) Oxygen ion dynamics in the Earth’s ring current: Van Allen Probes observations. J Geophys Res Space Phys 124(10):7786–7798. 10.1029/2019JA026801

[CR408] Zahn JP (2008) Tidal dissipation in binary systems. In: Goupil MJ, Zahn JP (eds) EAS Publications Series, pp 67–90. 10.1051/eas:0829002. arXiv:0807.4870

[CR409] Zahnle KJ, Catling DC (2017) The cosmic shoreline: the evidence that escape determines which planets have atmospheres, and what this may mean for Proxima Centauri B. Astrophys J 843(2):122. 10.3847/1538-4357/aa7846. arXiv:1702.03386 [astro-ph.EP]

[CR410] Zahnle KJ, Kasting JF (1986) Mass fractionation during transonic escape and implications for loss of water from Mars and Venus. Icarus 68(3):462–480. 10.1016/0019-1035(86)90051-5

[CR411] Zahnle K, Haberle RM, Catling DC, et al. (2008) Photochemical instability of the ancient Martian atmosphere. J Geophys Res Planets 113(E11):E11004. 10.1029/2008JE003160

[CR412] Zasova LV, Moroz VI, Linkin VM, et al. (2006) Structure of the Venusian atmosphere from surface up to 100 km. Cosm Res 44(4):364–383. 10.1134/S0010952506040095

[CR413] Zeng L, Sasselov DD, Jacobsen SB (2016) Mass-radius relation for rocky planets based on PREM. Astrophys J 819(2):127. 10.3847/0004-637X/819/2/127. arXiv:1512.08827 [astro-ph.EP]

[CR414] Zhang Q, Barabash S, Holmstrom M, et al (2024) Mars’s induced magnetosphere can degenerate. Nature 1–3. 10.1038/s41586-024-07959-z 10.1038/s41586-024-07959-z PMC11446820 39294383

[CR415] Zhao J, Tian F (2015) Photochemical escape of oxygen from early Mars. Icarus 250:477–481. 10.1016/j.icarus.2014.12.032. arXiv:1501.04423 [astro-ph.EP]

[CR416] Zhao J, Tian F, Ni Y, et al. (2017) DR-induced escape of O and C from early Mars. Icarus 284:305–313. 10.1016/j.icarus.2016.11.021

[CR417] Zhu X, Strobel DF, Erwin JT (2014) The density and thermal structure of Pluto’s atmosphere and associated escape processes and rates. Icarus 228:301–314. 10.1016/j.icarus.2013.10.011

[CR418] Zieba S, Kreidberg L, Ducrot E, et al. (2023) No thick carbon dioxide atmosphere on the rocky exoplanet TRAPPIST-1 c. Nature 620(7975):746–749. 10.1038/s41586-023-06232-z 37337068 10.1038/s41586-023-06232-z PMC10447244

